# Novel Triazole–Diquinothiazine Hybrids Modulating Apoptosis and Stress Pathways in Colorectal Cancer Cells

**DOI:** 10.3390/ph19081133

**Published:** 2026-07-23

**Authors:** Klaudia Giercuszkiewicz-Haśnik, Magdalena Skonieczna, Beata Morak-Młodawska, Małgorzata Jeleń

**Affiliations:** 1 Department of Systems Biology and Engineering, Silesian University of Technology, Akademicka Street 16, 44-100 Gliwice, Poland; magdalena.skonieczna@polsl.pl; 2 Centre of Biotechnology, Silesian University of Technology, Krzywoustego Street 8, 44-100 Gliwice, Poland; 3 Faculty of Medical Sciences in Katowice, Medical University of Silesia, 40-752 Katowice, Poland; 4 Department of Organic Chemistry, Faculty of Pharmaceutical Sciences in Sosnowiec, Medical University of Silesia in Katowice, Jagiellońska Street 4, 41-200 Sosnowiec, Poland; bmlodawska@sum.edu.pl

**Keywords:** diquinothiazines, 1,2,3-triazoles, phenothiazines, colorectal cancer, apoptosis regulation, *MDM2*-p53 interaction

## Abstract

**Background/Objectives:** Colorectal cancer is one of the most common malignancies worldwide and is frequently associated with dysregulation of apoptosis, oxidative stress, and the p53 regulatory axis. The development of novel compounds capable of modulating these pathways remains an important challenge in anticancer drug discovery. This study aimed to evaluate the biological activity of newly synthesized triazole–diquinothiazine derivatives in colorectal cancer cells and to identify the most promising lead compound for further development. **Methods:** The synthesized derivatives were evaluated in HCT116 colorectal cancer cells harboring wild-type TP53 and in normal BEAS-2B cells. Cytotoxicity, apoptosis, cell cycle distribution, intracellular reactive oxygen species (ROS) generation, and the expression of genes associated with apoptosis, oxidative stress, and inflammatory response were analyzed. **Results:** The investigated compounds exhibited diverse biological effects, including modulation of apoptosis, cell cycle progression, and oxidative stress. Among the tested derivatives, compound **B5** demonstrated the most favorable biological profile, characterized by a more pronounced biological response in HCT116 cells than in BEAS-2B cells, increased ROS generation, reduced MDM2 expression, and a marked induction of early apoptosis. **Conclusions:** The obtained results indicate that triazole–diquinothiazine derivatives represent promising lead structures for further optimization. In particular, compound **B5** warrants additional mechanistic and preclinical studies to evaluate its potential as a candidate for colorectal cancer therapy.

## 1. Introduction

Azole heterocycles have been one of the fundamental pillars of medicinal chemistry for many decades, and the 1,2,3-triazole ring occupies a special position among them due to its high chemical and metabolic stability, predictable geometry, and significant structural tolerance to various substituents. An additional advantage of this structural unit is the possibility of its efficient synthesis using click chemistry methods, especially through the CuAAC (Copper-Catalysed Azide–Alkyne Cycloaddition) reaction, which enables the rapid construction of libraries of compounds with high structural diversity and high purity. As a result, 1,2,3-triazoles have become a key motif in the design of new bioactive molecules, including compounds with anticancer and immunomodulatory potential and those targeting specific molecular targets [1,2,3,4,5,6]. The 1,2,3-triazole ring is widely used as a bioisostere, particularly in relation to amide groups and other polar fragments. This allows key interactions with the molecular target to be maintained while improving metabolic stability and ADME parameters. The triazole structure promotes the formation of hydrogen bonds, π-π and dipole–dipole interactions, which facilitates the modulation of ligand–protein interactions. In addition, this unit often acts as a linker connecting different pharmacophores with different physicochemical properties, providing a stable and conformationally favourable structural ‘bridge’ [7,8]. Numerous reports describe 1,2,3-triazole hybrids conjugated with systems such as quinazolines, naphthoquinones, indoles, coumarins, quinolinones, rhodanines and phenothiazines, exhibiting a broad spectrum of biological activity—from anticancer and antibacterial to neuroprotective effects [9,10,11,12,13]. In recent years, 1,2,3-triazole derivatives have been developed as inhibitors of key cancer-related pathways. Stilbene– and chalcone–triazole conjugates targeting the colchicine site of β-tubulin inhibit microtubule polymerisation and arrest the cell cycle [14], while 4-anilinoquinazoline and PI3Kγ-selective triazole derivatives demonstrate strong cytotoxic and submicromolar activity [15]. The translational potential of this scaffold is exemplified by the KIT pan-inhibitor AZD3229 [16], as well as dual Topo/HDAC hybrids with nanomolar potency and proapoptotic effects [17,18]. Chromene–triazole and oxadiazole-linked hybrids show promising EGFR inhibition [17,18] and systematic analyses confirm broad EGFR-targeting potential [19]. Additionally, acridine–triazole derivatives exhibit strong activity in the NCI-60 panel [20], while triazole sulfonamides selectively inhibit CA IX under hypoxic conditions [21,22].

1,2,3-Triazole derivatives have been investigated for immunomodulatory activity, including regulation of cytokine secretion and key immune enzymes. 1,4-Disubstituted bis-triazoles inhibit pro-inflammatory cytokines (TNF-α, IL-6, GM-CSF, IL-12p40) in LPS-activated macrophages via PI3K pathway modulation, suggesting potential in autoimmune and chronic inflammatory diseases [23]. Additionally, triazole-based IDO1 inhibitors block tryptophan metabolism, relieve immunosuppression, and restore T cell proliferation with nanomolar potency [24].

The 1,2,3-triazole ring has also been used to modify phenothiazine systems. Phenothiazine–1,2,3-triazole hybrids exhibit strong anticancer activity against gastric cancer. Liu et al. [25] identified a derivative with high efficacy against MGC-803 (IC_50_ = 1.2 μM) that inhibits migration (modulation of EMT markers), suppresses the Wnt/β-catenin pathway, and inhibits tubulin polymerisation; in an in vivo model, it limited xenograft growth without significant toxicity. Furthermore, 1,2,3-triazolo-phenothiazines exhibited in vitro activity against various cancer cell lines, including MGC-803, EC-109, PC-3, MCF-7, and HepG-2, with IC_50_ values ranging from 0.5 to 27.3 μM. The introduction of a fluorine atom into the benzene ring of the substituent further increased the biological activity of the tested derivatives [26]. In contrast, Zhang et al. [27] demonstrated that the 1,2,3-triazole moiety determines the anticancer activity of phenothiazine hybrids against breast cancer cell lines (MDA-MB-231, MDA-MB-468, MCF-7); its removal resulted in a loss of activity (IC_50_ > 100 μM). The most active derivative (IC_50_ 0.8–1.7 μM) induced apoptosis of MCF-7 cells by modulating BCL2 family proteins, PARP and DR5.

**Figure 1:**
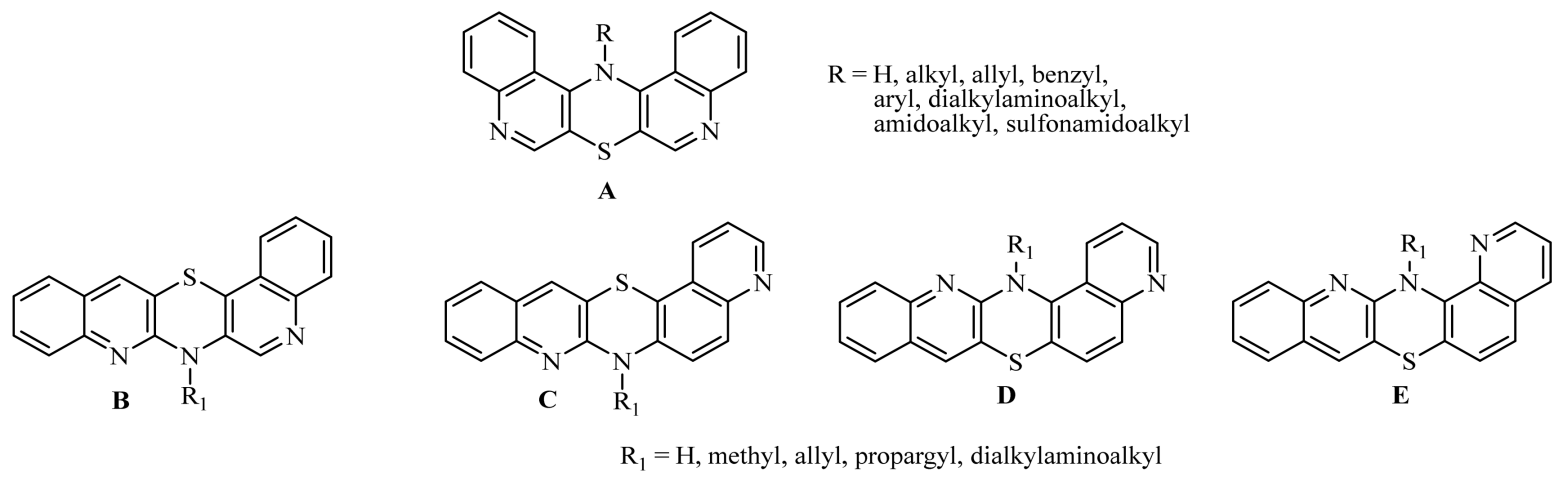
Angularly fused diquinothiazines [28,29,30].

Diquinothiazines are modified phenothiazine derivatives from the azaphenothiazine group, in which benzene rings have been replaced with quinoline systems, resulting in isomeric, pentacyclic structures with angular or linear condensation. The differences concern the manner in which the quinoline ring is coupled with the 1,4-thiazine system. The first structure of this class described initiated further research on 14*H*-1,4-thiazino[2,3-c;6,5-c′]diquinoline derivatives with various substituents at the nitrogen atom of the thiazine ring (Figure 1) [28,29,30]. The double angularly condensed diquinothiazines with aminoalkyl, amidoalkyl, sulfonamidoalkyl and phenyl substituents tested showed anticancer activity against the HCT116, SH-SY5Y, A549 and H1299 cell lines with low cytotoxicity towards normal BEAS-2B cells, often surpassing etoposide in terms of efficacy and selectivity. The most active compound was 14-(methylthiophenyl)diquinothiazine (IC_50_ 2.3–2.7 µM for most lines), while the H1299 line showed the lowest sensitivity. The compounds induced apoptosis and S-phase cell cycle arrest, and SAR analysis confirmed the key role of the substituent type at the nitrogen atom and cell line specificity in determining the biological effect [31].

Angularly condensed diquinothiazines **A**–**E** (Figure 1) also exhibit significant anticancer activity. Most N-alkyl and N-dialkylaminoalkyl derivatives achieved very high cytotoxicity (IC_50_ < 3 μM) against at least one cell line (SNB-19, Caco-2, MDA-MB-231, A549), often surpassing cisplatin, with the strongest effect observed against A549 (IC_50_ up to 0.3 μM). As a rule, dialkylaminoalkyl analogues were more active. Compounds substituted at position 7 (series **B** and **D**; exo-orientation) exceeded the activity of analogues at position 14 (**C** and **E**; endo orientation), which emphasises the importance of condensation topology and substituent accessibility for interaction with the biological target. The most active derivatives exhibited several times lower cytotoxicity towards NHDF fibroblasts, than toward cancer cells. Gene expression analysis confirmed proliferation inhibition (H3) and mitochondrial apoptosis activation (BAX/BCL2) [32], and for **B** with dimethylaminopropyl substituent—selective activity against A549 and induction of necrotic phases of the cell cycle [33].

**Figure 2:**
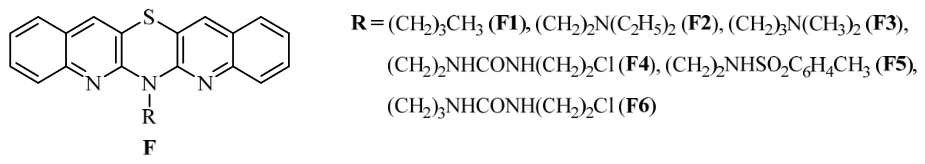
Linearly fused diquinothiazines.

Linearly condensed diquino[3,2-b;2′,3′-e]diquinothiazines[1,4]thiazines (Figure 2) also showed significant anticancer activity. The butyl derivative **F1** strongly inhibited the growth of breast cancer cells (MCF-7, T-47D) and kidney cancer cells (UO-31), but the highest activity and selectivity were observed for compounds from the **F2**–**F6** series. The strongest effect was demonstrated by compound **F4** with a chloroethylureidoethyl substituent (GI_50_ = 87 nM against SK-MEL-5 melanoma) [34,35].

Diquinothiazine containing the chloroureidoethyl substituent **F4** has also been shown to have immunosuppressive effects in in vivo models of delayed hypersensitivity, contact reactions and experimental psoriasis [36,37,38]. Furthermore, inhibition of IFNβ expression and IFNβ-dependent genes and proteins involved in the pathogenesis of autoimmune diseases has been demonstrated [39].

Molecular hybridization represents a rational strategy in medicinal chemistry aimed at integrating complementary pharmacophoric elements from distinct bioactive scaffolds into a single multifunctional entity. Such an approach seeks not only to combine or potentiate pharmacodynamic effects, but also to optimize pharmacokinetic properties and mitigate resistance or toxicity issues [40,41,42]. Within the paradigm of polypharmacology, hybrid molecules are particularly attractive in oncology, where simultaneous modulation of multiple signaling pathways may enhance therapeutic efficacy [43].

In our previous studies, we demonstrated that hybrid quinobenzothiazine derivatives containing a 1,2,3-triazole moiety represent a promising structural platform showing differential biological activity in cancer and non-cancer cell models [44]. The triazole unit was incorporated at the thiazine nitrogen using Huisgen 1,3-dipolar cycloaddition (“click chemistry”), providing efficient access to structurally defined hybrid architectures [45,46,47]. The structure–activity relationship (SAR) analysis revealed the key role of both the triazole moiety and the nature of the benzyl substituent in modulating cytotoxicity, apoptosis induction and the impact on cell survival pathways. These results confirmed the validity of further targeted optimisation of the molecular architecture to increase biological efficacy and selectivity. In this paper, we present the next generation of analogues designed based on a rational scaffold refinement strategy. Key modifications included replacing the benzyl fragment with a phenyl ring within the 1,2,3-triazole system, which allows for precise modulation of electronic parameters and π-π interaction topology, and the replacement of the quinobenzothiazine core with a more complex and conformationally stiffened diquinoinothiazine system. We assumed that increased planarisation and expansion of the system of fused heteroaromatic rings would translate into enhanced interactions with molecular targets and an improved biological activity profile.

## 2. Results

Based on the molecular hybridization strategy and our previous structure-activity relationship studies on triazole-containing quinobenzothiazine derivatives, a new series of hybrid compounds was designed and synthesized. The structural modifications introduced in the present work were aimed at further optimization of the pharmacophoric framework through replacement of the benzyl substituent in the 1,2,3-triazole fragment with a phenyl ring and incorporation of a conformationally more rigid diquinothiazine scaffold. The target compounds were obtained using a synthetic approach based on the Cu(I)-catalyzed azide–alkyne cycloaddition (CuAAC) reaction, which enabled efficient construction of the 1,2,3-triazole linker and provided access to structurally well-defined molecular hybrids. The synthetic route, together with the characterization of the obtained derivatives and evaluation of their biological activity, is discussed below.

### 2.1. Synthesis of 1,2,3-Triazolo-Diquinothiazines

**Scheme 1:**
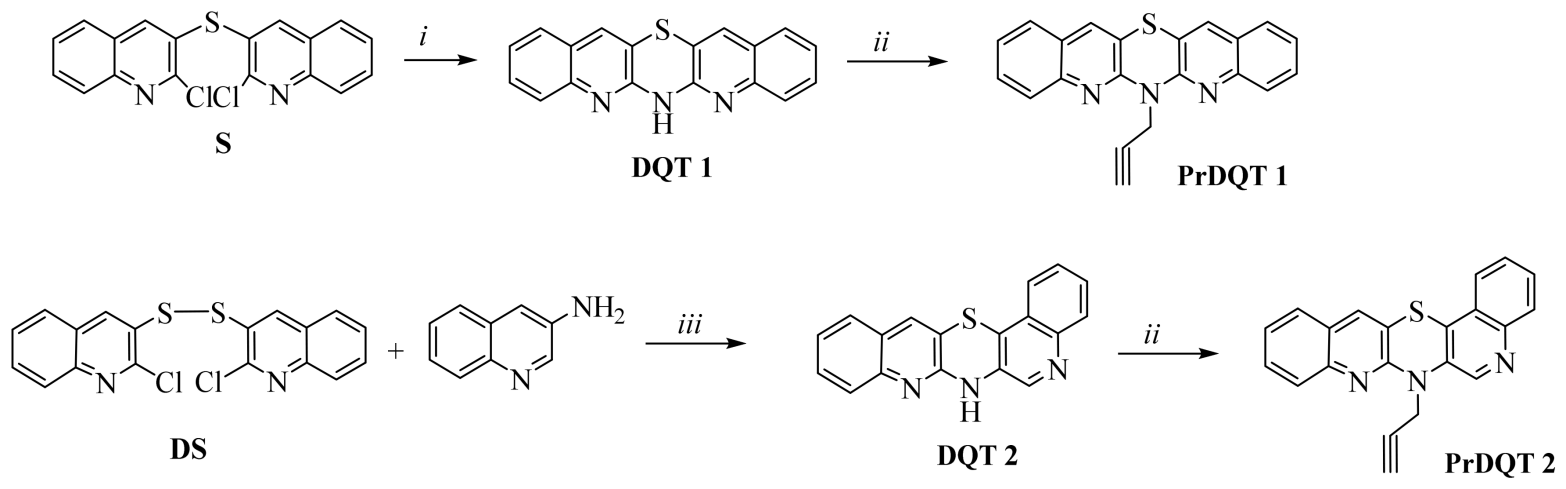
Synthesis of propargyldiquinothiazines **PrDQT 1** and **PrDQT 2**. Reagents and reaction conditions: (i) CH_3_CONH_2_, K_2_CO_3_, 180 °C [35]; (ii) DMF, NaH, ambient temperature, 24 h [32,48]; (iii) MEDG, reflux, 3 h [32].

We began our research with the synthesis of the appropriate diquinothiazines **DQT1** and **DQT2**, the basic substrates for the subsequent stages of the three-step synthesis of the title 1,2,3-triazolo-diquinothiazines **1**–**10**. We obtained linearly condensed diquinothiazine **DQT1** according to a previously developed and described synthesis based on 2,2′-dichloro-3,3′-diquinolyl sulphide **S** in acetamide in the presence of K_2_CO_3_ [35]. Angularly condensed diquinothiazine **DQT2** was obtained by the reaction of 2,2′-dichloro-3,3′-diquinolyl disulphide **DS** with 3-aminoquinoline in MEDG [32]. In the next step, we converted N-unsubstituted diquinothiazines into N-propynyl derivatives in an alkynylation reaction with 2-propynyl bromide against sodium hydride in DMF (Scheme 1) [32,48].

**Scheme 2:**
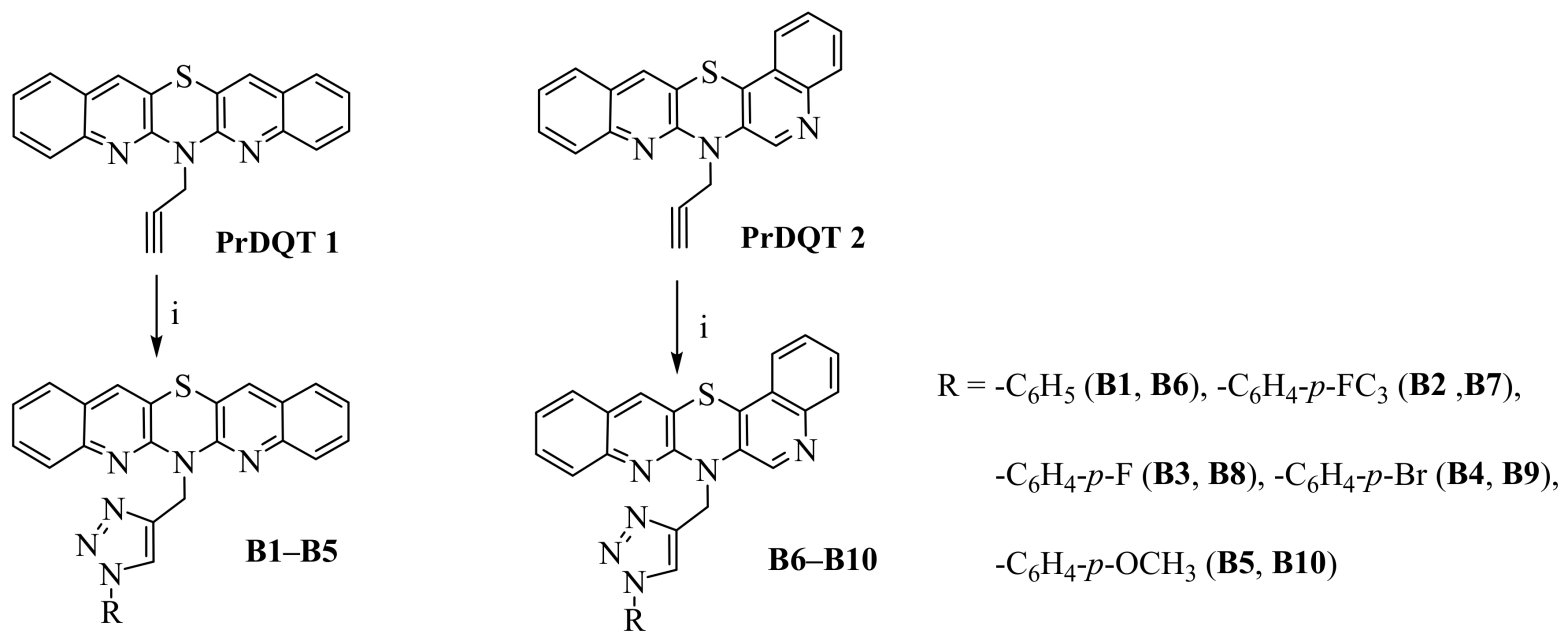
Synthesis of 1,2,3-triazolodiquinothiazines **B1**–**B10**. Reagents and reaction conditions: (i) toluene, copper iodide(I), organic azide, 70 °C, 72 h.

In the final stage of the synthetic sequence, the obtained intermediates were subjected to a 1,3-dipolar cycloaddition reaction (Cu(I)-catalyzed azide–alkyne cycloaddition, CuAAC; “click” chemistry) with selected organic azides: phenyl azide, p-trifluoromethylphenyl azide, p-fluorophenyl azide, p-bromophenyl azide, and p-methoxyphenyl azide. The reactions were carried out in toluene in the presence of CuI as the catalyst, affording the corresponding substituted 1,2,3-triazole derivatives **B1**–**B10** (Scheme 2, Table 1). The crude products were purified by column chromatography to yield pure compounds. Structural elucidation was accomplished by ^1^H and ^13^C NMR spectroscopy, and the molecular compositions were further confirmed by high-resolution mass spectrometry (HRMS). All spectroscopic data are provided in the Supplementary Information.

### 2.2. Research on Biological Activity

The biological activity of the synthesized compounds was evaluated using in vitro methods. The conducted studies included microscopic analysis of cell morphology, assessment of cytotoxic activity and IC_50_ values, evaluation of intracellular reactive oxygen species (ROS) levels, cell cycle analysis, apoptosis assessment, and analysis of the expression of genes associated with apoptosis, oxidative stress, and inflammatory responses.

#### 2.2.1. Microscopic Assessment of Cell Morphology

**Figure 3:**
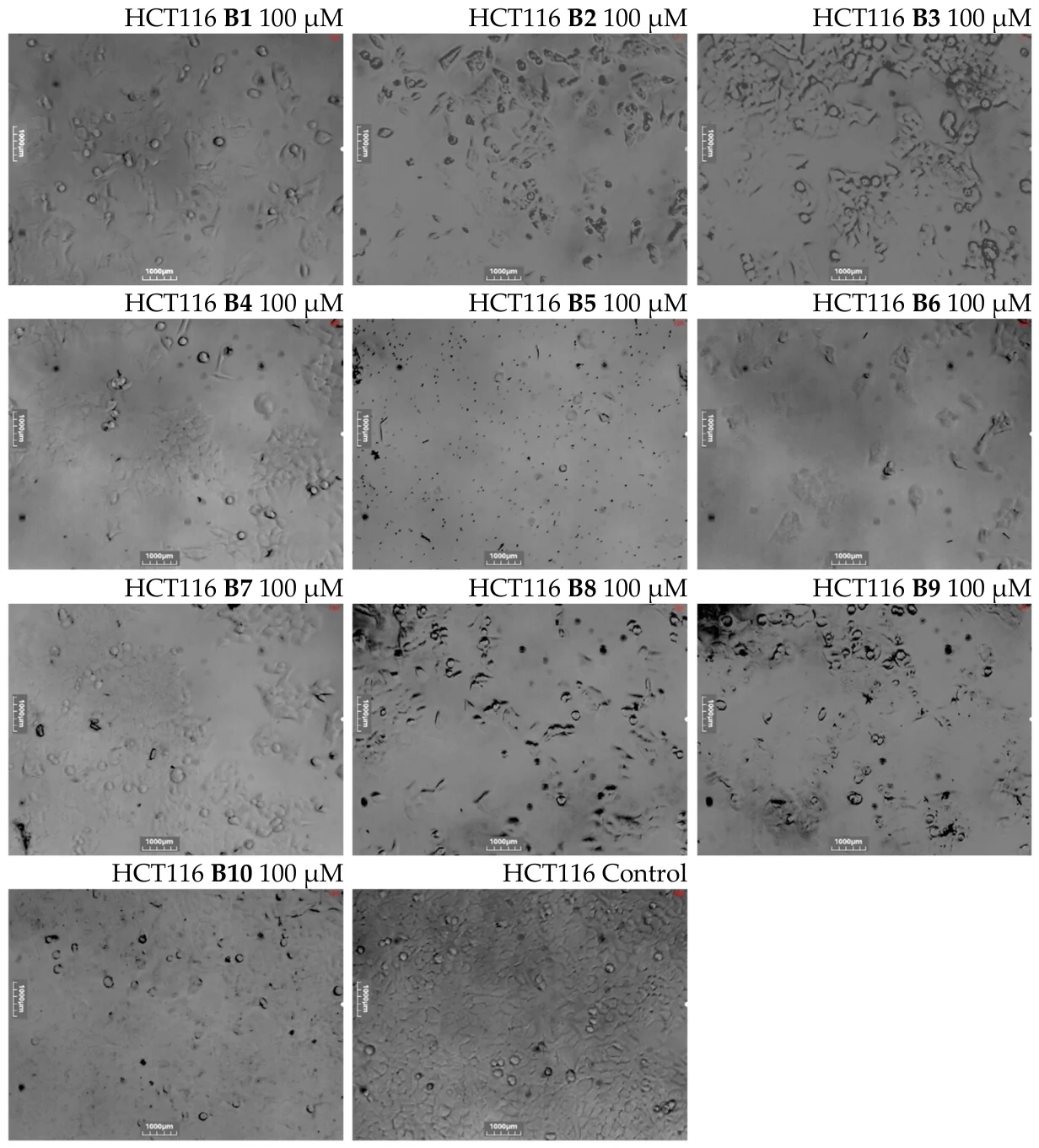
Microscopic images of HCT116 cells after 24 h incubation with compounds **B1**–**B10** at a concentration of 100 µM under standard culture conditions (37 °C, 5% CO_2_). Images were acquired at 10× magnification.

**Figure 4:**
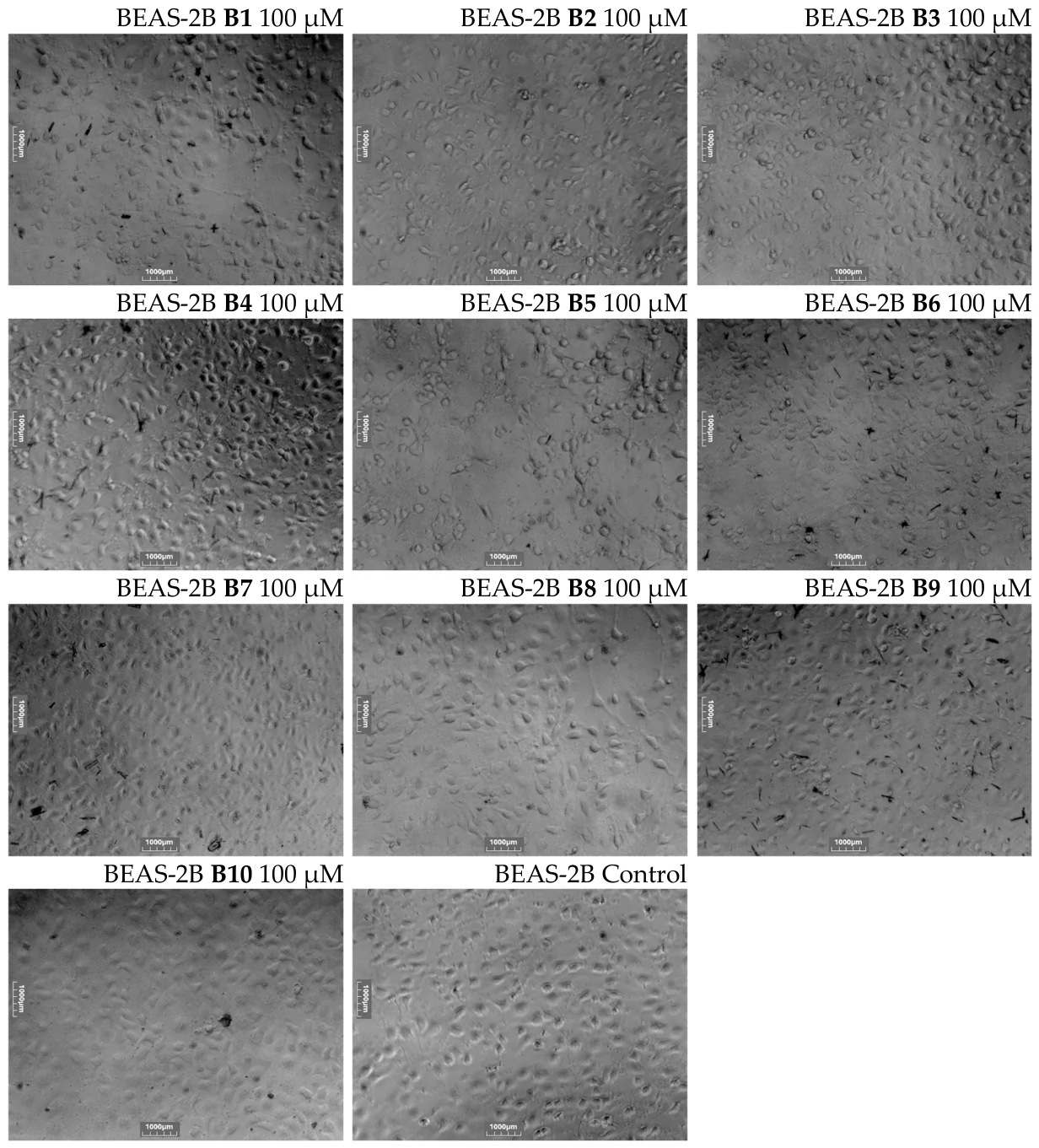
Microscopic images of BEAS-2B cells after 24 h incubation with compounds **B1**–**B10** at a concentration of 100 µM under standard culture conditions (37 °C, 5% CO_2_). Images were acquired at 10× magnification.

Microscopic analysis was performed using HCT116 cells with wild-type p53 status and BEAS-2B cell lines following 24 h incubation with compounds **B1**–**B10** at a concentration of 100 µM under standard culture conditions (37 °C, 5% CO_2_). The obtained photographs are presented in Figure 3 for HCT116 cells and in Figure 4 for BEAS-2B cells.

Microscopic evaluation revealed moderate morphological alterations in HCT116 colorectal cancer cells following 24 h incubation with the analyzed compounds at a concentration of 100 µM. In comparison with the control group, selected compounds, particularly **B2**, **B5**, **B6**, **B7**, **B8**, and **B9**, were associated with slight reductions in cell density, the presence of rounded cells, and partial disturbances in normal cellular morphology. Although the observed changes were not extensive, they may suggest the induction of cellular stress and reduced proliferative activity in HCT116 cells. Among the analyzed compounds, **B5** was particularly notable, as treated HCT116 cells exhibited visible detachment from the culture surface, which may indicate reduced cell adhesion and enhanced induction of cell death-related processes.

In contrast, BEAS-2B cells generally retained their characteristic epithelial morphology and confluency following treatment with the analyzed compounds. Although slight decreases in cell density and minor morphological alterations were observed for selected compounds, the overall changes were less pronounced than those detected in HCT116 cells. The observed differences between the two cell lines indicate that the analyzed compounds affected HCT116 and BEAS-2B cells to different extents.

#### 2.2.2. Alamar Blue Test

Based on the obtained cell viability results, IC_50_ values were determined for the tested compounds (Table 2). This parameter represents the concentration of a compound required to reduce cell viability by 50% relative to the control and is considered one of the fundamental indicators of cytotoxic activity. Lower IC_50_ values indicate higher anticancer activity of the tested compound. The obtained values are presented as mean ± standard deviation (SD) calculated from three replicates. In cases where IC_50_ was not reached within the tested concentration range up to 100 µM, the value was estimated by linear extrapolation and treated as an approximate value. IC_50_ values that could not be estimated within the tested concentration range were reported as IC_50_ > 100 µM. The 24 h exposure time was selected as an initial cytotoxicity assessment point and was used consistently as the reference condition for subsequent biological analyses. This approach allowed direct comparison between cell viability and the early cellular responses induced by the tested compounds under the same treatment conditions. Representative dose–response curves used for IC_50_ determination are provided in the Supplementary Materials (Figure S1).

The tested compounds from series B demonstrated diverse cytotoxic activity against HCT116 cells. Compound **B5** exhibited the highest anticancer activity, showing the lowest IC_50_ value (79.9 ± 0.7 µM). Relatively low IC_50_ values were observed for compounds **B1**, **B2**, **B4**, **B8** and **B10** indicating higher cytotoxic activity toward cancer cells compared to the other tested derivatives; however, for compounds considered as potentially cytotoxic agents, lower IC_50_ values would generally be expected. For most tested compounds, IC_50_ values could not be determined in BEAS-2B cells within the tested concentration range, suggesting lower cytotoxicity toward normal cells compared to HCT116 cells. Only compound **B10** exhibited measurable IC_50_ values in both HCT116 and BEAS-2B cell lines, which may indicate lower selectivity of this derivative. Overall, the obtained results indicate that compounds from series B exhibit moderate cytotoxic activity against HCT116 cells while showing limited toxicity toward normal cells under the tested experimental conditions. Although the IC_50_ values remained relatively high, the observed differences in cytotoxicity suggest a tendency toward greater sensitivity of HCT116 cells compared with BEAS-2B cells rather than confirmed cancer cell selectivity. These findings support further investigation of the most active derivatives, particularly with respect to their mechanism of action.

Since the analyzed compounds exhibited only moderate cytotoxic activity after 24 h incubation, further mechanistic experiments were performed using a concentration of 100 µM in order to obtain measurable biological responses. The use of a uniform concentration for all compounds enabled direct comparison of their effects on apoptosis, ROS generation, cell cycle progression, and gene expression under identical experimental conditions. Moreover, despite the relatively high concentration used, most compounds did not reach IC_50_ values in BEAS-2B cells, which allowed the evaluation of early cellular responses in both cell lines under identical treatment conditions rather than the assessment of confirmed cancer cell selectivity, while maintaining sufficient viable cell populations for downstream analyses. One limitation of the present study is that the stability of the tested compounds in the cell culture medium was not assessed. Therefore, it cannot be ruled out that the chemical stability of some derivatives affected their effective concentration during incubation, which may have influenced the observed biological activity.

#### 2.2.3. RT-qPCR

**Figure 5:**
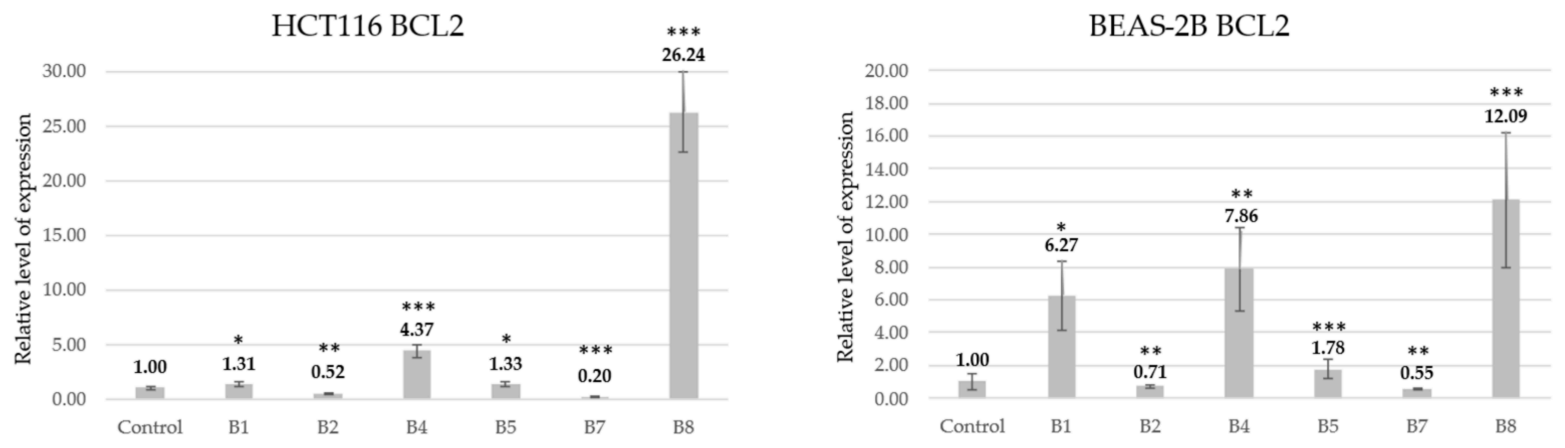

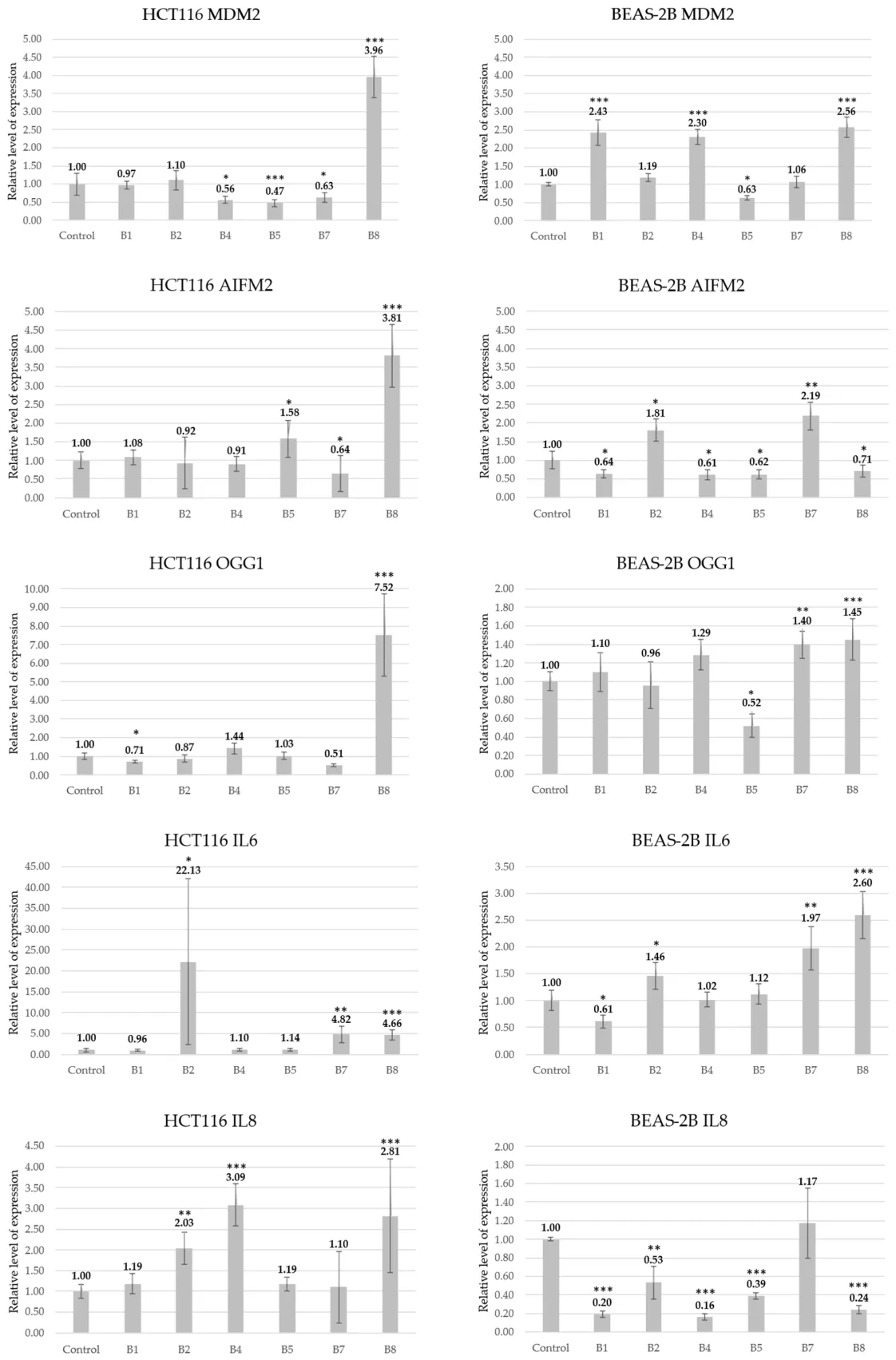
Relative expression levels of *BCL2*, *AIFM2*, *MDM2*, *OGG1*, *IL6*, and *IL8* genes in BEAS-2B and HCT116 cell lines following 24 h incubation with the tested compounds at a concentration of 100 μM. Results are presented as mean ± SD from three independent experiments and were compared with the untreated control group. Statistical significance was defined as an adjusted *p*-value (*q*) < 0.05. Significance levels are indicated as follows: * *q* < 0.05; ** *q* < 0.01; *** *q* < 0.001.

Real-time polymerase chain reaction (RT-qPCR) analysis was performed for the reference gene *RPL41* (ribosomal protein L41) and the target genes *BCL2*, *AIFM2*, *MDM2*, *OGG1*, *IL6*, and *IL8*. The selected target genes were chosen to investigate the potential effects of the analyzed compounds on various cellular pathways involved in apoptosis induction, oxidative stress regulation, inflammatory signaling, DNA damage response, and survival-related mechanisms. *RPL41* was selected as the reference gene due to its stable expression profile and the absence of significant variability under different experimental conditions and between the analyzed cell lines. The experiments were conducted using HCT116 and BEAS-2B cells. Gene expression analysis was performed for selected compounds that demonstrated the most promising biological activity in the preliminary experiments and were therefore chosen for further molecular evaluation. The expression levels of the selected genes were assessed after 24 h incubation with the tested compounds at a concentration of 100 μM (Figure 5). Expression values were calculated using the 2^−ΔΔCt^ method and are presented as mean ± SD (*n* = 3). Statistical significance was determined using ΔCt values and an unpaired two-tailed Welch’s t-test. To account for multiple hypothesis testing, p-values were adjusted using the Benjamini–Hochberg false discovery rate (FDR) procedure separately for each gene and cell line. Statistical significance was defined as an adjusted *p*-value (*q*) < 0.05. Significance levels are indicated as follows: * *q* < 0.05; ** *q* < 0.01; *** *q* < 0.001. Absence of asterisks indicates no statistically significant differences compared to the control group.

The *BCL2* gene encodes one of the most important antiapoptotic proteins, which inhibits activation of the mitochondrial apoptotic pathway and enables cancer cell survival despite the presence of DNA damage or oxidative stress. *BCL2* overexpression is frequently associated with resistance to anticancer therapy and increased survival of cancer cells [49]. For *BCL2*, the strongest increase in expression was observed following treatment with compound **B8** in both HCT116 and BEAS-2B cells, although the effect was more pronounced in the cancer cell line. Such a high level of *BCL2* expression may indicate activation of compensatory antiapoptotic mechanisms in response to severe cellular stress. A similar, although less pronounced, effect was also observed for compounds **B4** and **B1**. In contrast, compounds **B2** and **B7** reduced *BCL2* expression below the control level, particularly in HCT116 cells, suggesting a more classical proapoptotic profile. In BEAS-2B cells, the observed changes were generally less pronounced. Compound **B5** induced a slight increase in *BCL2* expression in both cell lines; however, this response was more evident in BEAS-2B cells than in HCT116 cells, which may indicate partial selectivity toward cancer cells.

MDM2 is the main negative regulator of the p53 protein and is responsible for its ubiquitination and proteasomal degradation. Overexpression of *MDM2* leads to suppression of p53 activity, thereby promoting cancer cell survival and tumor progression [50]. For the *MDM2* gene, the most favorable profile was observed for compounds **B5** and **B7**, which markedly reduced gene expression in HCT116 cells. These findings may indicate activation of the p53 axis and inhibition of mechanisms promoting cancer cell survival. Compound **B5** appeared particularly interesting, as it did not simultaneously induce strong activation of *MDM2* in normal cells. In contrast, **B8** exhibited a different profile, causing a pronounced increase in *MDM2* expression in both HCT116 and BEAS-2B cells. This may suggest activation of secondary compensatory mechanisms associated with severe cellular stress. Compounds **B1** and **B4** also increased *MDM2* expression in BEAS-2B cells, which may indicate a greater impact of these derivatives on normal cells compared with **B5**. In turn, compound **B2** did not significantly alter MDM2 expression in either HCT116 or BEAS-2B cells.

AIFM2/FSP1 is a protein associated with oxidative stress regulation and one of the key inhibitors of ferroptosis. By limiting lipid peroxidation, AIFM2 may protect cells from oxidative stress-dependent cell death; therefore, its overexpression may promote cancer cell survival [51]. Analysis of *AIFM2* expression revealed markedly different response profiles between the tested compounds and cell lines. The strongest increase in expression in HCT116 cells was observed following treatment with compound **B8**, which may indicate activation of an intense protective response associated with oxidative stress. A weaker effect was observed for compound **B5**, which increased *AIFM2* expression in HCT116 cells while simultaneously decreasing its expression in BEAS-2B cells. In contrast, compounds **B1**, **B2**, and **B4** did not exert significant effects in cancer cells, although they showed variable effects in BEAS-2B cells. The most interesting profile was observed for compound **B7**. In HCT116 cells, **B7** reduced *AIFM2* expression, whereas in BEAS-2B cells an increase in gene expression was observed. This result may suggest differential modulation of AIFM2/FSP1 expression between cancer and normal cells, which could potentially influence cellular responses to oxidative stress.

*OGG1* encodes a DNA glycosylase involved in the repair of oxidative damage to nitrogenous bases, particularly 8-oxoguanine, thereby protecting cells against mutation accumulation and genomic instability. However, in cancer cells, increased *OGG1* expression may also promote cell survival through more efficient repair of stress-induced DNA damage [52]. In the expression analysis, the least favorable profile was observed for compound B8, which caused a very strong increase in *OGG1* expression in HCT116 cells. An increase in *OGG1* expression was also observed in BEAS-2B cells, although the effect was considerably weaker. A much more favorable profile was observed for compounds **B1**, **B2**, and particularly **B7**, which reduced *OGG1* expression in HCT116 cells while maintaining or moderately increasing its expression in BEAS-2B cells. Such a profile may suggest increased susceptibility of cancer cells to the accumulation of oxidative DNA damage while preserving protective mechanisms in normal cells. This effect was most pronounced for **B7**, which may indicate beneficial selective modulation of oxidative stress response mechanisms; however, the observed changes did not reach statistical significance.

*IL6* and *IL8* are among the key pro-inflammatory cytokines involved in tumor progression, angiogenesis, cancer cell migration, and activation of survival-related signaling pathways such as JAK/STAT3. Elevated production of these cytokines is frequently associated with a more aggressive cancer phenotype, increased therapy resistance, and poorer prognosis [53,54]. Analysis of *IL6* expression revealed highly diverse response profiles among the tested compounds. The strongest induction of *IL6* was observed following treatment with compound **B2** in HCT116 cells, which may indicate activation of an intense pro-inflammatory response potentially promoting tumor progression. Moderate increases in expression were also observed for **B7** and **B8**, although these effects were clearly less pronounced. In contrast, compounds **B1**, **B4**, and **B5** did not significantly affect *IL6* expression levels in HCT116 cells. In BEAS-2B cells, a favorable profile was observed for **B1**, which reduced *IL6* expression, as well as for **B4** and **B5**, which exerted almost no effect on gene expression levels. A partially different response profile was observed for *IL8* expression. The strongest increase in *IL8* expression in HCT116 cells was induced by **B4**, followed by **B8** and **B2**, which may suggest activation of mechanisms associated with cancer cell migration and remodeling of the tumor microenvironment. The remaining compounds, namely **B1**, **B5**, and **B7**, did not significantly affect *IL8* expression levels. At the same time, in BEAS-2B cells, most compounds led to reduced *IL8* expression. The only exception was **B7**, which caused a moderate but statistically insignificant increase in *IL8* expression in BEAS-2B cells. Overall, the obtained results suggest that compounds **B2** and, to some extent, **B8** exhibited the least favorable profile regarding modulation of pro-inflammatory cytokines, particularly in HCT116 cancer cells. In contrast, **B5** demonstrated the most balanced biological profile, without strong *IL6* induction and with simultaneous reduction in *IL8* expression in BEAS-2B cells.

#### 2.2.4. Cell Cycle

**Figure 6:**
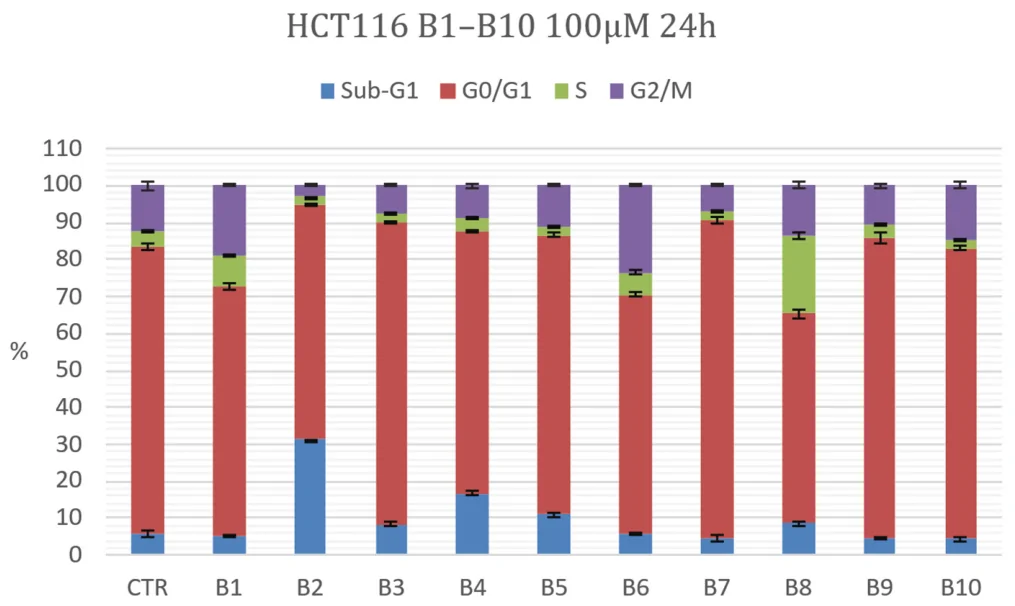
Effect of the tested compounds on cell cycle distribution in HCT116 cells after 24 h treatment. The percentages of cells in the sub-G1, G0/G1, S, and G2/M phases were determined by flow cytometry. Data are presented as mean ± SD from three experiments.

**Figure 7:**
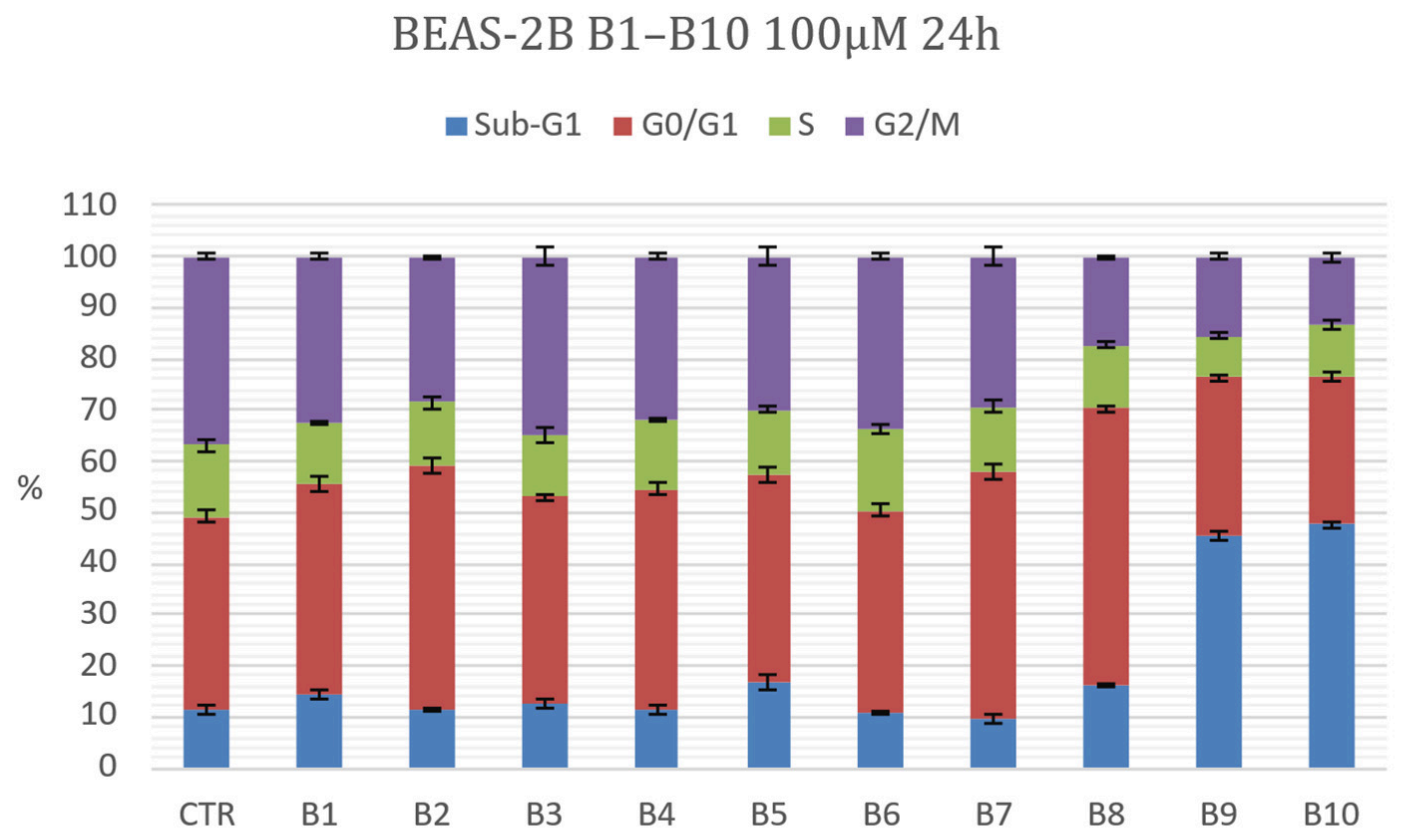
Effect of the tested compounds on cell cycle distribution in BEAS-2B cells after 24 h treatment. The percentages of cells in the sub-G1, G0/G1, S, and G2/M phases were determined by flow cytometry. Data are presented as mean ± SD from three experiments.

To assess the effect of the synthesized compounds on cell cycle progression and cell death, flow cytometric analysis was performed in HCT116 and BEAS-2B cells following 24 h treatment. The distribution of cells in the sub-G1, G0/G1, S, and G2/M fractions was evaluated to identify changes associated with DNA fragmentation, cell cycle arrest, or phase-specific cytostatic effects. Particular attention was paid to differences between cancerous and non-cancerous cells in order to identify compounds with differential biological response. The obtained results are presented in Figure 6 and Figure 7. Representative cell cycle histograms are provided in the Supplementary Materials (Figure S2).

Cell cycle analysis demonstrated that compounds from the **B** series induced diverse biological effects in HCT116 and BEAS-2B cells, including apoptosis induction, cell cycle arrest at different phases, and potential replication stress. At the same time, some compounds exhibited greater selectivity toward cancer cells, whereas others showed stronger effects in BEAS-2B cells.

In HCT116 cells, compound **B1** caused a moderate shift in cells toward the G2/M phase accompanied by a slight increase in the S-phase population, which may suggest activation of the G2/M checkpoint in response to DNA damage or mitotic disturbances. This effect was not observed in BEAS-2B cells, indicating partial selectivity toward cancer cells. **B2** exhibited the strongest proapoptotic effect in HCT116 cells, increasing the sub-G1 population to over 31% while markedly reducing the proportion of cells in the S and G2/M phases. This profile suggests intensive induction of cell death without dominant cell cycle arrest. In BEAS-2B cells, no analogous increase in sub-G1 was observed, indicating favorable anticancer selectivity. **B3** caused a slight increase in the G0/G1 population together with a minor reduction in the S and G2/M fractions in HCT116 cells. No significant changes in phase distribution were observed in BEAS-2B cells, suggesting limited influence of this compound on the cell cycle. **B4** induced a moderate increase in the sub-G1 fraction in HCT116 cells accompanied by a reduction in the G2/M population. This may indicate apoptosis induction without pronounced arrest at a specific cell cycle checkpoint. In normal cells, the effect was limited. **B5** exhibited moderate proapoptotic activity in HCT116 cells, increasing the sub-G1 population. At the same time, no clear accumulation of cells in the S or G2/M phases was observed, which may suggest more direct activation of cell death mechanisms rather than classical cell cycle arrest. In BEAS-2B cells, the observed changes remained moderate. **B6** caused pronounced accumulation of HCT116 cells in the G2/M phase together with an increased S-phase population. This profile may indicate induction of DNA damage, replication stress, or mitotic disturbances activating the G2/M checkpoint. In BEAS-2B cells, the effect was considerably weaker, suggesting partial selectivity toward cancer cells. **B7** induced a distinct shift in cell cycle distribution in HCT116 cells, characterized by an increased G0/G1 population and a reduced proportion of cells in the S and G2/M phases. Such a profile suggests effective inhibition of the G1/S transition and strong suppression of cellular proliferation. In BEAS-2B cells, only moderate changes were observed. **B8** induced a very pronounced increase in the S-phase population in HCT116 cells, suggesting arrest during DNA replication and potential replication stress. At the same time, no strong increase in the sub-G1 fraction was observed, which may indicate that the cytotoxic effect develops secondary to earlier disturbances in DNA synthesis. In BEAS-2B cells, the compound mainly increased the G0/G1 population while reducing the G2/M fraction. **B9** did not exhibit a significant proapoptotic effect in HCT116 cells; however, in BEAS-2B cells it caused a very strong increase in the sub-G1 population. Simultaneously, the proportion of cells in the G2/M phase markedly decreased. This profile indicates high toxicity toward normal cells and low anticancer selectivity. A similar profile was observed for **B10**. In HCT116 cells, cell cycle alterations were minor, whereas in BEAS-2B cells the compound induced a very strong increase in the sub-G1 population, indicating intensive cell death. These findings suggest an unfavorable safety profile and lack of selectivity toward cancer cells.

#### 2.2.5. Level of Apoptosis

**Figure 8:**
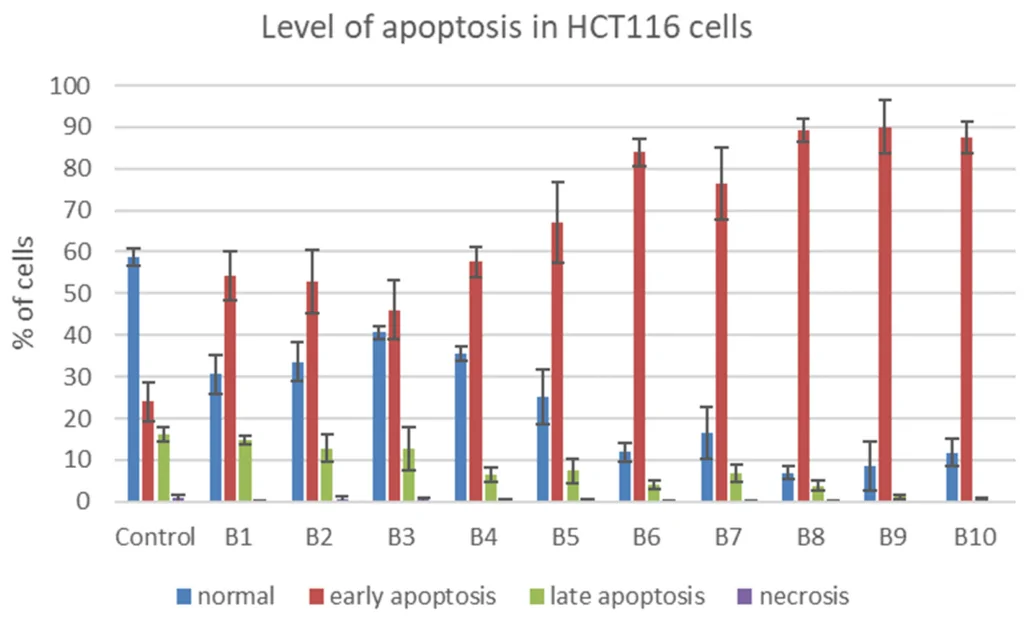
Apoptosis analysis of HCT116 colorectal cancer cells after 24 h incubation with compounds **B1**–**B10** at a concentration of 100 µM. The graph presents the percentages of viable cells, early apoptotic cells, late apoptotic cells, and necrotic cells determined by Annexin V/PI staining and flow cytometry following 24 h treatment.

**Figure 9:**
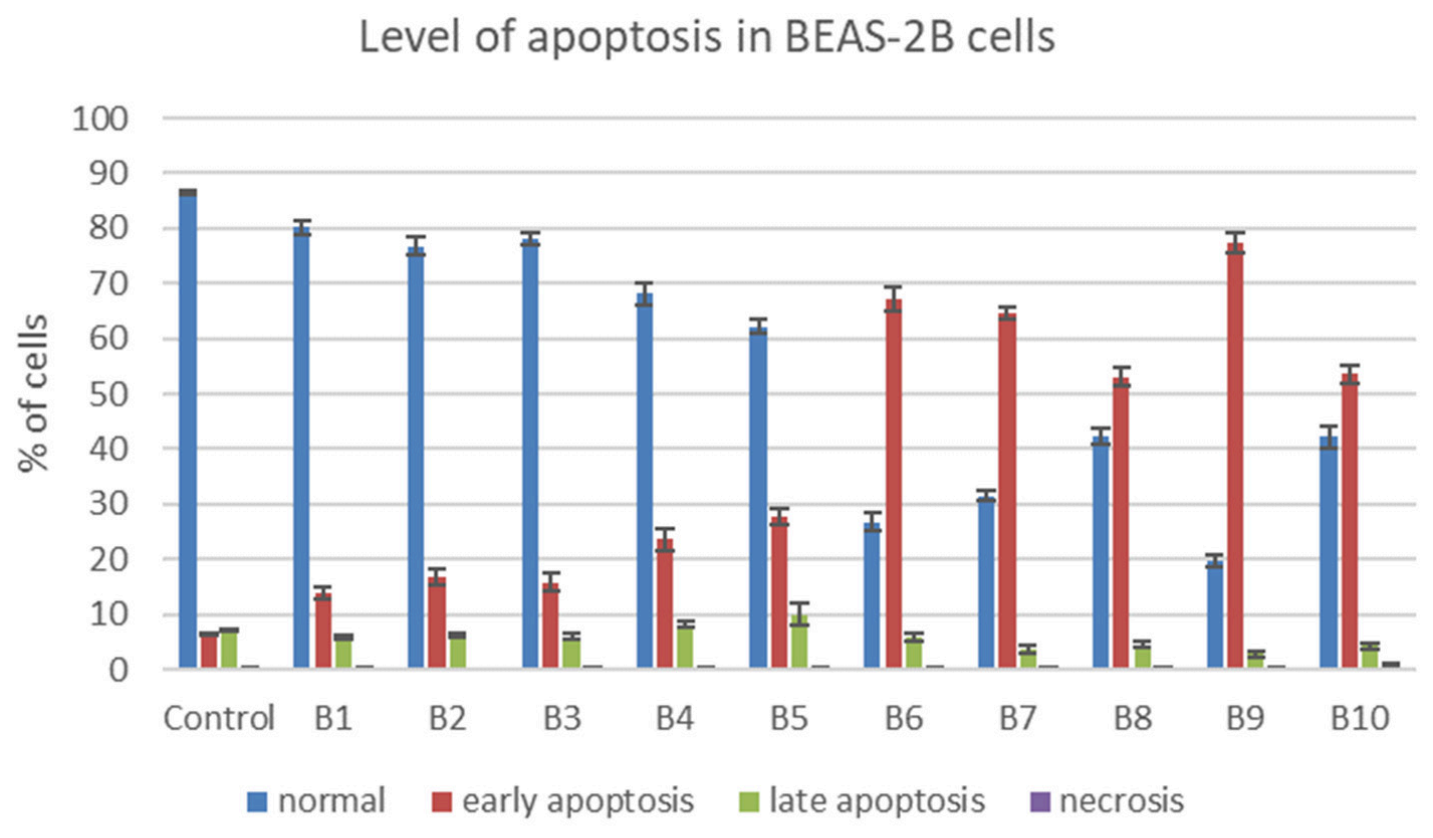
Apoptosis analysis of BEAS-2B cells after 24 h incubation with compounds **B1**–**B10** at a concentration of 100 µM. The graph presents the percentages of viable cells, early apoptotic cells, late apoptotic cells, and necrotic cells determined by Annexin V/PI staining and flow cytometry following 24 h treatment.

Apoptosis analysis was performed to determine whether the cytotoxic effects of the tested compounds were associated with induction of programmed cell death and to evaluate the selectivity of this effect between cancer and normal cells. Since activation of apoptosis is one of the key mechanisms underlying the activity of many anticancer agents, assessment of early apoptosis, late apoptosis, and necrosis-enabled characterization of the predominant type of cell death induced by the analyzed compounds in HCT116 and BEAS-2B cells is necessary. The obtained results are presented in Figure 8 and Figure 9. Representative Annexin V-FITC/PI dot plots are provided in the Supplementary Materials (Figure S3).

The overall apoptosis analysis demonstrated that compounds from the **B** series induced markedly different biological responses in HCT116 and BEAS-2B cells. Most of the analyzed compounds primarily induced early apoptosis while maintaining very low levels of necrosis, suggesting activation of regulated cell death mechanisms rather than nonspecific cytotoxicity. It should be noted, however, that the representative Annexin V-FITC/PI dot plots shown in Figure S3 revealed the presence of a fraction of Annexin V/PI double-positive cells in the untreated controls, which likely reflects the basal level of spontaneous cell death in cultured cells. The lower proportion of these cells observed after treatment with the most active compounds may be associated with extensive fragmentation occurring during the late stages of apoptosis. Consequently, apoptotic bodies and cellular debris may be partially lost during sample preparation and flow cytometric acquisition, affecting the distribution of events between individual quadrants. Apoptosis analysis revealed that all tested compounds from the **B** series increased the proportion of early apoptotic cells in HCT116 colorectal cancer cells compared with the control group. The strongest proapoptotic effect was observed for compounds **B5**–**B10**, which caused a pronounced increase in the early apoptotic population accompanied by a substantial reduction in the percentage of viable cells. At the same time, the levels of late apoptosis and necrosis remained relatively low, indicating that the analyzed compounds predominantly activated programmed cell death rather than nonspecific necrotic toxicity. In BEAS-2B cells, a different response profile was observed. Compounds **B6**–**B10** also increased the proportion of early apoptotic cells, which may limit their potential therapeutic applicability. In contrast, for compounds **B1**–**B5**, viable cells still represented the dominant population, indicating a weaker cytotoxic effect toward normal cells. These findings suggest that several compounds induced stronger apoptotic responses in HCT116 cells than in BEAS-2B cells.

Among all analyzed derivatives, compound **B5** exhibited the most favorable biological profile. In HCT116 cells, **B5** induced the strongest increase in early apoptosis among compounds **B1**–**B5**, whereas in BEAS-2B cells it did not cause a comparably high increase in the apoptotic population, with viable cells still remaining predominant. These findings indicate that **B5** elicited a stronger apoptotic response in HCT116 cells than in BEAS-2B cells under the experimental conditions used.

#### 2.2.6. Reactive Oxygen Species Level

**Figure 10:**
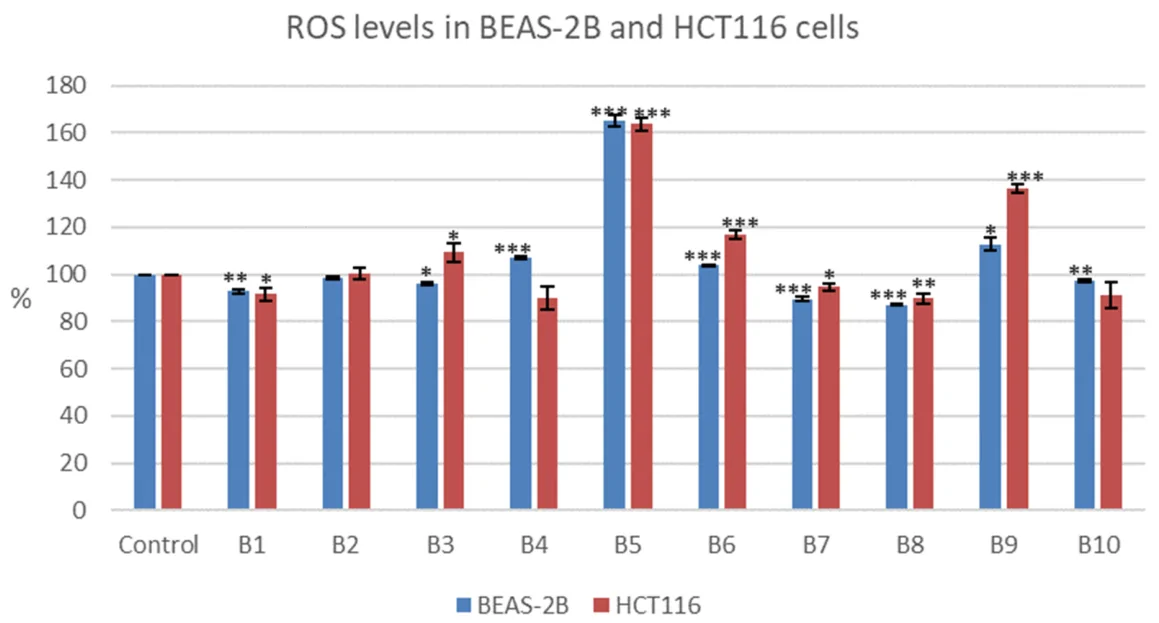
ROS levels in HCT116 and BEAS-2B cells after 24 h treatment with compounds **B1**–**B10** at a concentration of 100 µM. Results are presented as percentages relative to the control group. Statistical significance vs. control: * *p* < 0.05, ** *p* < 0.01, *** *p* < 0.001.

Analysis of reactive oxygen species (ROS) levels was performed to evaluate whether the tested compounds induced oxidative stress in cancer and normal cells. Excessive ROS production may lead to DNA damage, mitochondrial dysfunction, and activation of pathways associated with apoptosis and cell death. At the same time, moderate modulation of ROS levels may play an important role in the mechanism of action of potential anticancer agents. Comparison of ROS levels between HCT116 and BEAS-2B cells additionally enabled assessment of differences in oxidative stress responses between HCT116 and BEAS-2B cells (Figure 10). Representative ROS histograms are provided in the Supplementary Materials (Figure S4).

Analysis of reactive oxygen species (ROS) levels demonstrated that compounds from the **B** series induced diverse oxidative responses in HCT116 and BEAS-2B cells. Some compounds caused a pronounced increase in ROS levels, which may suggest induction of oxidative stress as one of the mechanisms underlying their anticancer activity, whereas others reduced ROS levels or did not induce significant changes compared with the control group.

The strongest induction of oxidative stress was observed for compound **B5**; however, this effect occurred in both cancer and normal cells, which may indicate limited selectivity. Compounds **B3**, **B6**, and **B9** exhibited a greater increase in ROS levels in HCT116 cells, suggesting the involvement of oxidative stress in their anticancer activity. In contrast, compounds **B1**, **B2**, **B4**, **B7**, **B8**, and **B10** exerted only minor effects on ROS levels or led to moderate decreases/increases relative to the control group, suggesting a limited contribution of oxidative stress to their mechanism of action. A limitation of the present ROS analysis is the lack of a positive ROS-generating control. Therefore, the observed changes should be interpreted relative to the untreated control and not as a direct comparison with established ROS-inducing agents.

## 3. Discussion

Overall, the obtained results indicate that the newly synthesized 1,2,3-triazole–diquinothiazine hybrids represent promising lead structures for further optimization and biological evaluation in the search for novel anticancer agents. The analyzed derivatives exhibited diverse biological effects, including modulation of apoptosis, oxidative stress, cell cycle progression, and the expression of genes associated with cancer cell survival. The observed differences between individual compounds suggest that even subtle structural modifications may significantly influence both the mechanism of action and the differential biological responses observed between HCT116 and BEAS-2B cells. The collected data suggest that triazole–diquinothiazine hybrids may affect several cellular processes involved in cancer cell survival.

Among the analyzed compounds from series B, particular attention was drawn to **B2**, **B5**, and **B7**, as they exhibited the most interesting biological profiles in HCT116 cells. Each of these compounds affected cancer cells in a different manner, suggesting the involvement of distinct anticancer mechanisms.

Compound **B2** displayed a moderate proapoptotic response under the tested conditions. In cell cycle analysis, it caused the strongest increase in the sub-G1 population in HCT116 cells, while apoptosis analysis demonstrated a clear increase in the proportion of cells undergoing early apoptosis. In addition, decreased *BCL2* expression was observed in both analyzed cell lines, which may indicate weakening of antiapoptotic defense mechanisms. Importantly, **B2** did not induce a significant increase in ROS levels, suggesting that its activity is likely not primarily associated with oxidative stress. Despite these biological effects, the IC_50_ value in HCT116 cells remained relatively high, indicating limited cytotoxic potency in the concentration–response viability assay. Moreover, the strong upregulation of *IL6* and *IL8* observed in HCT116 cells may indicate activation of a proinflammatory response, reducing the attractiveness of **B2** as the leading candidate for further investigation.

Compound **B7** also demonstrated several distinct biological effects, including both cell cycle arrest and apoptosis induction. In HCT116 cells, **B7** caused a marked increase in the G0/G1 population accompanied by a reduction in S and G2/M phase cells, consistent with inhibition of G1/S progression and reduced proliferative activity. In addition, **B7** decreased the expression of *BCL2*, *MDM2*, *AIFM2*, and *OGG1* in HCT116 cells, which may suggest impairment of cellular survival and protective mechanisms. However, **B7** also markedly increased the proportion of early apoptotic HCT116 cells, indicating that its activity cannot be regarded as predominantly cytostatic. A similarly pronounced increase in early apoptosis was observed in BEAS-2B cells, suggesting limited selectivity of this response between the two cell lines. Although **B7** exhibited an interesting combined cytostatic and cytotoxic profile at 100 µM, its IC_50_ value remained high, reduced indicating relatively low potency in the concentration–response viability assay. Therefore, **B7** was not considered the most promising lead compound for further mechanistic studies.

The most favorable and internally consistent biological profile was observed for compound **B5**. Among all analyzed derivatives, **B5** showed the strongest cytotoxic activity toward HCT116 cells, reaching the lowest IC_50_ value (79.9 ± 0.7 µM), while IC_50_ was not reached in BEAS-2B cells within the tested concentration range. This result indicates a more pronounced effect in HCT116 cells profile already at the level of cytotoxicity, although the obtained IC_50_ value should still be considered relatively high. Although the compounds exhibited only moderate cytotoxicity after 24 h, several derivatives, particularly **B5**, produced pronounced mechanistic changes, including apoptosis induction and oxidative stress, suggesting that these compounds may serve as valuable lead structures for further optimization rather than highly potent cytotoxic agents in their current form. The observed downregulation of MDM2 in HCT116 cells suggests that the MDM2–p53 regulatory axis may be involved in the response to **B5**; however, direct modulation of this pathway was not investigated and requires further validation.

Apoptosis analysis revealed that **B5** induced one of the strongest increases in the early apoptotic population in HCT116 cells while maintaining relatively low levels of late apoptosis and necrosis, suggesting activation of organized programmed cell death mechanisms rather than nonspecific toxicity. Cell cycle analysis showed only a moderate increase in the sub-G1 population without clear arrest in a specific phase of the cell cycle, indicating that **B5** acts mainly through direct induction of cell death rather than through a classical cytostatic mechanism. Notably, the pronounced proapoptotic effect observed after 24 h treatment was not fully reflected by the viability assay. This may be related to the fact that cells in the early stages of apoptosis can still retain metabolic activity and therefore contribute to the Alamar Blue signal. Consequently, activation of apoptotic pathways may occur before a substantial decrease in overall cell viability becomes apparent.

One of the most characteristic features of **B5** was a very strong increase in ROS levels in both HCT116 and BEAS-2B cells, indicating a mechanism associated with oxidative stress. In HCT116 cells, this effect was accompanied by pronounced apoptosis induction and the lowest IC_50_ value among the analyzed derivatives, suggesting that HCT116 cells were more susceptible to the prooxidative effects of **B5** under the experimental conditions used. In contrast, despite elevated ROS levels in BEAS-2B cells, IC_50_ was not reached within the tested concentration range, which may reflect higher tolerance or more efficient compensatory mechanisms in normal cells exposed to oxidative stress.

Gene expression analysis further supported the biological profile of **B5**. In HCT116 cells, **B5** caused a clear decrease in *MDM2* expression, which may reduce p53 inhibition and facilitate activation of apoptosis. At the same time, only a slight increase in *BCL2* and *AIFM2*/*FSP1* expression was observed, which may represent a compensatory response to elevated oxidative stress that was nevertheless insufficient to prevent cell death. The lack of significant changes in *OGG1* expression suggests that classical DNA repair mechanisms associated with oxidative damage were not strongly activated. Importantly, **B5** did not induce marked upregulation of *IL6* and *IL8*, resulting in a less proinflammatory profile than that observed for **B2**.

In BEAS-2B cells, **B5** also decreased *MDM2* expression; however, this effect was accompanied by increased *BCL2* expression, which may represent a protective mechanism limiting apoptosis in normal cells. Furthermore, despite oxidative stress-related changes, viable cells still constituted the dominant population, distinguishing **B5** from compounds such as **B9** and **B10**, which produced more comparable responses in both cell lines.

Considering the obtained results, it is possible that the strong ROS increase induced by **B5** may contribute to activation of stress-response pathways potentially involving p53. If p53 signaling is involved, p53 activation could subsequently enhance the expression of pro-oxidative and pro-apoptotic factors, further increasing oxidative stress and promoting apoptosis through a positive feedback mechanism. Such a positive feedback mechanism between ROS and p53 may explain the high efficacy of **B5** in inducing HCT116 cell death. The observed decrease in MDM2 expression could potentially reduce p53 inhibition and contribute to the apoptotic response, although p53 activation was not directly evaluated. Taken together, these findings suggest that the biological response to **B5** may involve oxidative stress and changes in the MDM2/p53 regulatory axis. However, the direct involvement of p53 and other survival signaling pathways requires further experimental validation.

In summary, **B2**, **B5**, and **B7** may be considered the three most promising compounds from series **B**; however, only **B5** combined favorable outcomes across all major analyses, including cytotoxicity, apoptosis, ROS generation, and gene expression profiling. The cytotoxic potency of the synthesized compounds was generally moderate, which should be considered a limitation of the present study. Nevertheless, the observed proapoptotic activity and modulation of oxidative stress and gene expression suggest that these compounds may serve as lead structures for further structural optimization. **B2** exhibited clear proapoptotic activity, but its profile was weakened by increased expression of proinflammatory cytokines and only moderate cytotoxic efficacy. **B7** appeared interesting mainly as a cytostatic compound, although it did not sufficiently reduce HCT116 viability and produced similar biological responses in HCT116 and BEAS-2B cells. Therefore, **B5** was identified as the most interesting lead structure for further mechanistic studies.

## 4. Materials and Methods

### 4.1. Chemistry

^1^H, and ^13^C NMR, spectra were recorded on a Bruker Ascend 600 MHz spectrometer (Bruker, Rheinstetten, Germany) using CDCl_3_ as the solvent. High-resolution mass spectra (HRMS) were acquired by electrospray ionization (ESI) on a Bruker Impact II mass spectrometer (Bruker, Billerica, MA, USA). Melting points of solid compounds were determined in open capillaries using a Boetius melting point apparatus (Stuart Equipment, Stone, UK) and are reported without correction.

All reagents and solvents were obtained from commercial suppliers and used as received without further purification unless otherwise stated. Phenyl azides, propargyl bromide, potassium tert-butoxide, and copper(I) iodide were purchased from Sigma-Aldrich (Burlington, MA, USA). Toluene, N,N-dimethylformamide (DMF), and chloroform were supplied by POCh (Gliwice, Poland). 6*H*-Diquino[3,2-b;2′,3′-e][1,4]thiazine **DQT1**, 7*H*-diquino[3,2-b;3′,4′-e][1,4]thiazine **DQT2**, and their propynyl derivatives **PrDQT1** and –**PrDQT2** were obtained according to previously described methods [32,35,48,51].

*General procedure for synthesis of 1,2,3-triazole and diquinothiazine hybrids* **B1** *–* **B10**

Copper(I) iodide (0.006 g) was added to a solution of 6-propynyl diquinothiazine **PrDQT1**–**2** (0.5 mmol) in anhydrous toluene (10 mL), followed by the corresponding organic azide (0.510 mmol). The reaction was carried out at 70 °C for 72 h with continuous stirring. After completion of the process, the mixture was cooled to room temperature and the solvent was removed under reduced pressure. The resulting residue was dissolved in dichloromethane and purified by column chromatography on silica gel using CH_2_Cl_2_ as the eluent. As a result of this procedure, pure triazole derivatives **B1**–**B10** were isolated:

6-((1-phenyl-1*H*-1,2,3-triazol-4-yl)methyl)-diquino[3,2-b;2′,3′-e][1,4]thiazine (**B1**):

Yield: 78%. M.p.: 251–252 °C. ^1^H NMR (600 MHz, CDCl3) δ: 6.15 (s, 2H, CH_2_), 7.32–7.34 (m, 3H, H_Ph_), 7.44 (t, 2H, H-2, H-10, *J* = 16 Hz), 7.54–7.57 (m, 4H, H-3, H-9, 2H_Ph_), 7.65 (d, 2H, H-1, H-11, *J* = 7.7 Hz), 7.74 (s, 2H, H-12, H-14), 7.84 (d, 2H, H-4, H-8, *J* = 8.3 Hz), 7.99 (s, 1H, CH). ^13^C NMR (150 MHz, CDCl_3_) δ: 39.23, 116.24, 120.50, 121.34, 125.07, 125.81, 126.10, 127.88, 128.32, 129.49, 129.53, 131.77, 137.25, 145.48, 146.67, 149.50. HR MS (ESI) calcd for: C_27_H_19_N_6_S [M + H]^+^: 459.1392, found: 459.1390.

6-((1-(4-(trifluoromethyl)phenyl)-1*H*-1,2,3-triazol-4-yl)methyl)-diquino[3,2-b;2′,3′-e][1,4]thiazine (**B2**):

Yield: 80%. M.p.: 282–283 °C. ^1^H NMR (600 MHz, CDCl_3_) δ: 6.07 (s, 2H, CH_2_), 7.27 (t, 2H, H-2, H-10, *J* = 7.3 Hz), 7.45-7.50 (m, 4H, H-3, H-9, 2H_Ph_), 6.30 (d, 2H, H-1, H-11, *J* = 8.5 Hz), 7.68 (s, 2H, H-12, H-14), 7.72–7.76 (m, H-4, H-8, 2H_Ph_), 7.97 (s, 1H, CH). ^13^C NMR (150 MHz, CDCl_3_) δ: 39.37, 116.24, 120.36, 122.67, 124.48, 125.19, 125.83, 126.16, 126.90, 126.92, 127.85, 129.59, 130.24 (q, *J* = 33.0 Hz), 131.89, 139.45, 145.44, 149.46. ^19^F NMR (565 MHz, CDCl_3_) δ: −60.97. HR MS (ESI) calcd for: C_28_H_18_F_3_N_6_S [M + H]^+^: 527.1266, found: 527.1269.

6-((1-(4-fluorophenyl)-1*H*-1,2,3-triazol-4-yl)methyl)-diquino[3,2-b;2′,3′-e][1,4]thiazine (**B3**):

Yield: 69%. M.p.: 219–220 °C. ^1^H NMR (600 MHz, CDCl_3_) δ: 6.19 (s, 2H, CH_2_), 7.12–7.15 (m, 2H, 2H_Ph_), 7.36 (t, 2H, H-2, H-10, *J* = 7.3 Hz), 7.55–7.57 (m, 4H, H-1, H-11, H-3, H-9), 7.61–7.64 (m, 2H, 2H_Ph_), 7.75 (s, 2H, H-12,H-14), 7.87 (d, 2H, H-4, H-8, *J* = 8.4 Hz), 7.99 (s, 1H, CH). ^13^C NMR (150 MHz, CDCl_3_) δ: 39.38, 116.48 (d, *J* = 22.5 Hz), 121.61, 122.41, 122.46, 125.26, 125.79, 126.14, 127.69, 128.34 (d, *J* = 6 Hz), 129.66, 132.02, 133.59 (d, *J* = 3 Hz), 145.18, 146.53, 149.38, 162.18 (d, *J* = 247.5 Hz).

6-((1-(4-bromophenyl)-1*H*-1,2,3-triazol-4-yl)methyl)-diquino[3,2-b;2′,3′-e][1,4]thiazine (**B4**):

Yield: 60%. M.p.: 258–259 °C. ^1^H NMR (600 MHz, CDCl_3_) δ: 6.13 (s, 2H, CH_2_), 7.34 (t, 2H, H-2, H-10, *J* = 7.8 Hz), 7.54–7.58 (m, 8H, H-1, H-11, H-3, H-9, 4H_Ph_), 7.75 (s, 2H, H-12, H-14), 7.83 (d, 2H, H-4, H-8, *J* = 8.4 Hz), 7.96 (s, 1H, CH). ^13^C NMR (150 MHz, CDCl_3_) δ: 39.29, 116.22, 121.10, 121.85, 122.10, 125.13, 125.82, 126.12, 127.85, 129.53, 131.81, 132.66, 136.21, 145.46, 147.02, 149.47. HR MS (ESI) calcd for: C_27_H_18_BrN_6_S [M + H]^+^: 537.0497, found: 537.0498.

6-((1-(4-methoxyphenyl)-1*H*-1,2,3-triazol-4-yl)methyl)-diquino[3,2-b;2′,3′-e][1,4]thiazine (**B5**):

Yield: 58%. M.p.: 218–219 °C. ^1^H NMR (600 MHz, CDCl_3_) δ: 3.67 (s, 3H, CH_3_), 6.08 (s, 2H, CH_2_), 7.34 (t, 2H, H-2, H-10, *J* = 7.8 Hz), 7.54–7.57 (m, 2H, H-3, H-9), 7.61-7.63 (m, 4H, H-1, H-11, 2H_Ph_), 7.74 (m, 2H, 2H_Ph_), 7.84 (s, 2H, H-12, H-14), 7.92 (d, 2H, H-4, H-8, *J* = 9.0 Hz), 8.84 (s, 1H, CH). ^13^C NMR (150 MHz, CDCl_3_) δ: 27.48, 31.95, 115.70, 117.00, 120.50, 121.34, 125.69, 125.92, 125.99, 126.28, 128.07, 129.83, 131.92, 132.61, 145.16, 146.67, 148.53. HR MS (ESI) calcd for: C_28_H_21_N_6_OS [M + H]^+^: 489.1498, found: 489.1493.

7-((1-phenyl-1*H*-1,2,3-triazol-4-yl)methyl)-diquino[3,2-b;3′,4′-e][1,4]thiazine (**B6**):

Yield: 63%. M.p.: 236–239 °C. ^1^H NMR (600 MHz, CDCl_3_) δ: 5.66 (s, 2H, CH_2_), 7.26 (t, 1H, H-11, *J* = 7.1 Hz), 7.31 (t, 1H, H-10, *J* = 7.4Hz), 7.37–7.40 (m, 2H, H-2, H-12), 7.47–7.53 (m, 4H, 3-H, 3H_Ph_), 7.58 (d, 2H, 2H_Ph_), 7.67–7.68 (m, 2H, H-9, H-13), 7.81 (d, 1H, H-1, *J* = 7.26 Hz), 7.90 (d, 1H, H-4, *J* = 8.4 Hz), 8.05 (s, 1H, CH), 8.95 (s, 1H, H-6). ^13^C NMR (150 MHz, CDCl_3_) δ: 42.40, 116.22, 120.66, 122.05, 122.58, 124.46, 124.57, 124.91, 126.36, 126.49, 127.26, 127.42, 128.01, 128.71, 129.67, 129.77, 129.92, 132.87, 134.26, 137.06, 139.73, 144.28, 145.36, 145.71, 151.42. HR MS (ESI) calcd for: C_27_H_19_N_6_S [M + H]^+^: 459.1392, found: 459.1392.

7-((1-(4-(trifluoromethyl)phenyl)-1*H*-1,2,3-triazol-4-yl)methyl)-diquino[3,2-b;3′,4′-e][1,4]thiazine (**B7**):

Yield: 81%. M.p.: 269–270 °C. ^1^H NMR (600 MHz, CDCl_3_) δ: 5.67 (s, 2H, CH_2_), 7.28 (t, 1H, H-11, *J* = 7.2 Hz), 7.49-7.53 (m, 4H, H-2, H-3, H-10, H-12), 7.64–7.70 (m, 7H, H-1, H-9, H-13, 4H_Ph_), 7.94 (m, 2H, H-4, CH), 8.20 (s, 1H, H-6). ^13^C NMR (150 MHz, CDCl_3_) δ: 29.72, 42.07, 115.28, 120.32, 120.78, 122.26, 122.62, 124.42, 125.28, 125.74, 126.22, 126.39, 126.60, 127.05, 127.07, 127.30, 127.75, 128.30, 128.88, 129.06, 130.22, 130.70 (q, *J* = 32.9 Hz), 133.20, 139.39, 145.70, 150.95. ^19^F NMR (MHz, CDCl_3_) δ: −62.63. HR MS (ESI) calcd for: C_28_H_18_F_3_N_6_S [M + H]^+^: 527.1266, found: 527.1263.

7-((1-(4-fluorophenyl)-1*H*-1,2,3-triazol-4-yl)methyl)-diquino[3,2-b;3′,4′-e][1,4]thiazine (**B8**):

Yield: 67%. M.p.: 229–230 °C. ^1^H NMR (600 MHz, CDCl_3_) δ: 5.75 (s, 2H, CH_2_), 7.17–7.20 (m, 2H, H-10, H-11), 7.37 (t, 1H, H-2, *J* = 7.2 Hz), 7.58-7.61 (m, 3H, H-12, 2H_Ph_), 7.62–7.68 (m, 3H, 3H, 2H_Ph_), 7.76–7.79 (m, 2H, H-9, H-13), 7.93 (d, 1H, H-1, *J* = 8.4 Hz), 8.12 (s, 2H, H-4, CH), 9.02 (s, 1H, H-6). ^13^C NMR (150 MHz, CDCl_3_) δ: 40.69, 115.65 (d, *J* = 23 Hz), 115.68, 120.59, 121.27, 121.62, 121.67, 123.55, 124.47, 124.67, 125.33, 125.62, 126.41, 127.81, 128.43, 129.27, 129.43, 132.19, 132.40, 132.47, 133.93, 143.03, 144.72, 149.10, 161.41 (d, *J* = 248 Hz). HR MS (ESI) calcd for: C_27_H_18_FN_6_S [M + H]^+^: 477.1298, found: 477.1288.

7-((1-(4-bromophenyl)-1*H*-1,2,3-triazol-4-yl)methyl)-diquino[3,2-b;3′,4′-e][1,4]thiazine (**B9**):

Yield: 53%. M.p.: 234–235 °C. ^1^H NMR (600 MHz, CDCl_3_) δ: 5.66 (s, 2H, CH_2_), 7.27 (t, 1H, H-11, *J* = 7.1 Hz), 7.47–7.54 (m, 8H, H-2,H-3, H-10, H-12,4H_Ph_), 7.66–7.67 (d, 1H, H-9, *J* = 8.3 Hz), 7.68 (s, 1H, H-13), 7.80–7.82 (d, 1H, H-1, *J* = 8.2 Hz), 7.89–7.91 (d, 1H, H-4, *J* = 8.1 Hz), 8.03 (s, 1H, CH), 8.92 (s, 1H, H-6). ^13^C NMR (150 MHz, CDCl_3_) δ: 42.34, 116.22, 121.87, 122.01, 122.35, 122.59, 124.49, 124.97, 126.37, 126.52, 126.65, 127.21, 127.47, 127.67, 128.07, 129.78, 129.97, 132.82, 132.91, 134.18, 135.99, 139.63, 144.31, 145.68, 151.38. HR MS (ESI) calcd for: C_27_H_18_BrN_6_S [M + H]^+^: 537.0497, found: 537.0491.

7-((1-(4-methoxyphenyl)-1*H*-1,2,3-triazol-4-yl)methyl)-diquino[3,2-b;3′,4′-e][1,4]thiazine (**B10**):

Yield: 44%. M.p.: 241–242 °C. ^1^H NMR (600 MHz, CDCl_3_) δ: 3.85 (s, 3H, CH_3_), 5.72 (s, 2H, CH_2_), 6.97–7.00 (m, 2H, 2H_Ph_), 7.36–7.38 (m, 1H, H-11), 7.58–7.61 (m, 5H, H-2, H-9, H-12, 2H_Ph_), 7.65 (t, 1H, H-3, *J* = 7.9 Hz), 7.70 (t, 1H, H-10, *J* = 6.9 Hz), 7.77 (d, 1H, H-1, *J* = 8.4 Hz), 7.78 (s, 1H, H-13), 7.94–7.95 (d, 1H, H-4, *J* = 8.8 Hz), 8.11 (s, 1H, CH), 9.05 (s, 1H, H-6). ^13^C NMR (150 MHz, CDCl_3_) δ: 29.72, 48.84, 115.23, 122.63, 124.70, 125.02, 125.51, 126.39, 126.75, 127.38, 127.66, 127.93, 128.18, 128.30, 128.47, 128.98, 129.11, 129.29, 129.36, 129.95, 133.10, 133.16, 134.51, 136.35, 145.94, 151.34. HR MS (ESI) calcd for: C_28_H_21_N_6_OS [M + H]^+^: 489.1498, found: 489.1494.

### 4.2. Microscopic Assessment of Cell Morphology

The human colorectal cancer cell line HCT116 (ATCC CCL-247) and the normal human bronchial epithelial cell line BEAS-2B (ATCC CRL-9609) were obtained from the American Type Culture Collection (ATCC, Manassas, VA, USA).

The HCT116 cell line was selected as a colorectal cancer model due to its preserved wild-type p53 status together with activating KRAS and PIK3CA mutations, resulting in constitutive activation of MAPK and PI3K/AKT signaling pathways. This molecular background makes HCT116 a suitable model for investigating cellular responses associated with apoptosis, oxidative stress, and survival signaling in colorectal cancer cells retaining functional p53. Since one of the aims of this study was to evaluate whether the analyzed compounds may interfere with cancer cell survival mechanisms and promote p53-dependent anticancer responses, the use of HCT116 cells enabled assessment of these effects in the presence of strong oncogenic signaling pathways.

In contrast, the BEAS-2B cell line, representing non-cancerous human bronchial epithelial cells, was used as a reference non-malignant cell model to compare the biological responses observed in HCT116 and BEAS-2B cells. Comparison of the biological effects observed in HCT116 and BEAS-2B cells allowed assessment of differences in cellular responses between the two cell lines.

BEAS-2B and HCT116 cells were maintained in RPMI and DMEM media, respectively, supplemented with 10% fetal bovine serum (FBS) and 1% penicillin-streptomycin mixture. For microscopic evaluation, cells were plated in 96-well culture plates at a density of 1 × 10^4^ cells per well and allowed to adhere for 24 h under standard incubation conditions (37 °C, 5% CO_2_, humidified atmosphere). Subsequently, the analyzed compounds from the **B1**–**B10** series were introduced into the culture medium at a final concentration of 100 µM. Untreated cells cultured in the corresponding media served as the control group. After 24 h exposure to the tested compounds, morphological alterations were examined and documented using a JuLI™ FL Live fluorescence microscope (NanoEntek, Martinsried, Germany). The analysis included qualitative assessment of cell morphology, confluency, cellular organization, and adhesion to the culture surface.

### 4.3. Alamar Blue Test

The cytotoxicity of the tested compounds was evaluated using the Alamar Blue assay (Invitrogen, Thermo Fisher Scientific, Waltham, MA, USA). HCT116 and BEAS-2B cells were seeded into 96-well plates at a density of 1 × 10^4^ cells per well and cultured for 24 h under standard conditions (37 °C, 5% CO_2_, and appropriate humidity). The cells were then exposed to the tested compounds at concentrations ranging from 6.25 to 100 µM for 24 h. Untreated cells served as the control and were considered 100% viable. After treatment, 10 µL of Alamar Blue reagent was added to each well, and the plates were incubated for an additional 2–4 h under the same conditions. Fluorescence was measured using a microplate reader at excitation and emission wavelengths of 570 and 585 nm, respectively. Each concentration was tested in triplicate, and cell viability was expressed as a percentage relative to the untreated control.

IC_50_ values were determined based on cell viability curves as a function of compound concentration. For each technical replicate, a separate IC_50_ value was calculated using linear interpolation between two adjacent concentrations at which cell viability was above and below 50%. Subsequently, the mean IC_50_ value and standard deviation were calculated from the three obtained IC_50_ values. For comparative purposes, in selected cases, approximate linear extrapolation beyond the tested concentration range was performed; however, such values were treated solely as estimated values.

### 4.4. RT-qPCR

BEAS-2B and HCT116 cells were cultured in 6-well plates under standard culture conditions (37 °C, 5% CO_2_, appropriate humidity) in RPMI-1640 medium for BEAS-2B cells and DMEM for HCT116 cells, supplemented with 10% FBS and 1% penicillin/streptomycin. Cells were seeded at a density of 2 × 10^5^ cells per well. After 24 h, the tested compounds were added at a concentration of 100 µM and incubated for an additional 24 h. Following incubation, the cells were washed with PBS, detached using trypsin, and centrifuged. The obtained cell pellet was suspended in Fenozol and stored at −20 °C until RNA isolation.

Total RNA was isolated using the Total RNA Mini kit (A&A Biotechnology Gdynia, Gdansk, Poland) according to the manufacturer’s instructions. RNA concentration and purity were assessed spectrophotometrically using a NanoDrop 2000 instrument (Thermo Fisher Scientific, Waltham, MA, USA). Reverse transcription was subsequently performed using the EURx NG dART RT kit (EURx, Gdańsk, Poland) to obtain cDNA.

Gene expression analysis was carried out using the RT-qPCR method with Brilliant III Ultra Fast SYBR^®^ Green QPCR Master Mix (Agilent Technologies, Santa Clara, CA, USA) and specific primers for *BCL2*, *AIFM2*, *MDM2*, *OGG1*, *IL6*, and *IL8* genes (Table 3). *RPL41* was used as the reference gene. Reactions were performed using the Bio-Rad CFX96 Real-Time PCR Detection System (Bio-Rad, Hercules, CA, USA), while data analysis was conducted using CFX Maestro software (version 2.3) and Microsoft Excel.

Relative gene expression levels were calculated using the 2^−ΔΔCt^ method relative to the control group [55]. Results are presented as mean ± standard deviation (SD) from three independent biological experiments. Statistical analysis was performed using ΔCt values obtained from RT-qPCR experiments (*n* = 3). Differences between treated samples and the control group were evaluated using an unpaired two-tailed Welch’s t-test, which does not assume equal variances between groups. To account for multiple hypothesis testing, the resulting p-values were adjusted using the Benjamini–Hochberg false discovery rate (FDR) procedure separately for each gene and cell line. Statistical significance was defined based on the adjusted *p*-values as follows: * *q* < 0.05, ** *q* < 0.01, and *** *q* < 0.001, while the absence of asterisks indicated no statistically significant differences (*q* ≥ 0.05).

### 4.5. Cell Cycle Analysis

Cells were seeded into 6-well plates at a density of 3 × 10^5^ cells per well and incubated for 24 h under standard culture conditions (37 °C, 5% CO_2_, appropriate humidity) in RPMI medium for the BEAS-2B cell line and DMEM medium for HCT116 cells. Subsequently, the cells were treated with the tested compounds at a concentration of 100 µM and incubated for an additional 24 h. After incubation, the cells were collected, centrifuged (3000 rpm, 2 min), and washed with PBS. The obtained cell pellets were fixed in 70% ice-cold ethanol and stored at −20 °C until further analysis.

Prior to analysis, the samples were centrifuged again (3000 rpm, 2 min), the supernatant was removed, and the cells were washed twice with 500 µL of PBS. After the second wash, 50 µL of PBS was left above the cell pellet, followed by the addition of 100 µL of cold RNase solution (100 µg/mL, Merck KGaA, Darmstadt, Germany). Samples were incubated for 20 min at room temperature in the dark. Subsequently, 200 µL of propidium iodide solution (100 µg/mL; Merck KGaA, Darmstadt, Germany) was added. The samples were kept on ice in the dark, and cell cycle analysis was performed using a FACSAria™ III flow cytometer (Becton Dickinson Franklin Lakes, NJ, USA; 488 nm laser, LP 550, BP 575/25).

The cell cycle analysis method using propidium iodide (PI) is based on the determination of cellular DNA content by flow cytometry. Propidium iodide is a fluorescent dye that binds to double-stranded DNA, and the intensity of the emitted fluorescence is proportional to the amount of genetic material within the cell. This enables discrimination between cells in different phases of the cell cycle, including G0/G1, S, and G2/M. Cells in the G0/G1 phase contain the basal amount of DNA (2N), whereas cells in the S phase actively synthesize DNA and therefore exhibit intermediate fluorescence intensity. Cells in the G2/M phase contain doubled DNA content (4N). Additionally, the sub-G1 population corresponds to cells with reduced DNA content, characteristic of DNA fragmentation occurring during apoptosis. RNase treatment is applied to remove RNA that could interfere with the accurate measurement of DNA-associated fluorescence [56].

### 4.6. Analysis of Apoptotic Cell Death

Cells were seeded into 12-well plates at a density of 1 × 10^5^ cells per well and incubated for 24 h under standard culture conditions (37 °C, 5% CO_2_, appropriate humidity) in RPMI medium for the BEAS-2B cell line and DMEM medium for HCT116 cells. Subsequently, the cells were treated with the tested compounds at a concentration of 100 µM and incubated for an additional 24 h. After incubation, the cells were collected, centrifuged (3000 rpm, 2 min), and washed with PBS.

The cell pellet was resuspended in Annexin V Staining Buffer from the Annexin V-FITC Apoptosis Detection Kit (Invitrogen, Waltham, MA, USA), followed by the addition of Annexin V-FITC and propidium iodide (PI). Samples were incubated for 20 min at room temperature in the dark. Apoptotic cell death was subsequently analyzed using a FACSAria™ III flow cytometer (Becton Dickinson).

The method is based on double staining with Annexin V-FITC and propidium iodide (PI), enabling discrimination between viable, apoptotic, and necrotic cells by flow cytometry. Under physiological conditions, phosphatidylserine is located on the inner leaflet of the cell membrane; however, during early apoptosis, it translocates to the outer membrane surface, where it binds Annexin V-FITC. Propidium iodide does not penetrate viable or early apoptotic cells but enters cells with compromised membrane integrity, characteristic of late apoptosis and necrosis [57].

This method enables the distinction of viable cells (Annexin V−/PI−), early apoptotic cells (Annexin V+/PI−), late apoptotic cells (Annexin V+/PI+), and necrotic cells (Annexin V−/PI+).

### 4.7. Reactive Oxygen Species Analysis

Cells were seeded into 12-well plates at a density of 1 × 10^5^ cells per well and incubated for 24 h under standard culture conditions (37 °C, 5% CO_2_, appropriate humidity) in RPMI medium for the BEAS-2B cell line and DMEM medium for HCT116 cells. Subsequently, the cells were treated with the tested compounds at a concentration of 100 µM and incubated for an additional 24 h. After incubation, the cells were collected and stained with CellROX™ Green Reagent at a final concentration of 5 µM (Thermo Fisher Scientific, Waltham, MA, USA), according to the manufacturer’s instructions. Following incubation, the samples were analyzed using an FACSAria™ III flow cytometer (Becton Dickinson).

CellROX™ Green Reagent is a fluorogenic probe used for the detection of reactive oxygen species (ROS) in living cells. In its reduced form, the dye exhibits weak fluorescence; however, upon oxidation by ROS, it emits an intense green fluorescent signal while binding to cellular DNA. The fluorescence intensity is proportional to the level of oxidative stress within the cells, enabling the assessment of ROS generation by flow cytometry [57,58].

## 5. Conclusions

Although additional studies are required, the presented results indicate that this group of compounds has significant potential as a source of lead structures for anticancer drug development. Further studies are required to fully elucidate their molecular mechanisms of action, evaluate in vivo activity and toxicity, and optimize their pharmacokinetic properties. Nevertheless, the obtained data support the value of the 1,2,3-triazole–diquinothiazine scaffold for further structural optimization and biological evaluation. The value of these compounds stems not only from their effect on cell viability but also from the observed modulation of processes related to oxidative stress, apoptosis, and the expression of genes involved in the regulation of cell survival. Although the cytotoxic potency of the investigated compounds remains moderate, the broad range of biological effects observed for the most active derivatives, particularly B5, indicates that these compounds may represent valuable starting points for further structural optimization rather than immediate drug candidates.

## Figures and Tables

**Table 1:** Structural differences among compounds **B1**–**B10**.

Compound	Diquinothiazine Core	Aromatic Substituent Attached to the 1,2,3-triazole Ring (R)
**B1**	Linear	–C_6_H_5_
**B2**	Linear	–C_6_H_4_-*p*-CF_3_
**B3**	Linear	–C_6_H_4_-*p*-F
**B4**	Linear	–C_6_H_4_-*p*-Br
**B5**	Linear	–C_6_H_4_-*p*-OCH_3_
**B6**	Angular	–C_6_H_5_
**B7**	Angular	–C_6_H_4_-*p*-CF_3_
**B8**	Angular	–C_6_H_4_-*p*-F
**B9**	Angular	–C_6_H_4_-*p*-Br
**B10**	Angular	–C_6_H_4_-*p*-OCH_3_

**Table 2:** IC_50_ values of the tested compounds in HCT116 and BEAS-2B cell lines determined using the Alamar Blue assay after 24 h of incubation.

Compound	HCT116 24 h	BEAS-2B 24 h
**B1**	127.9 ± 22.7 µM	IC_50_ > 100 µM
**B2**	138.8 ± 9.7 µM	IC_50_ > 100 µM
**B3**	216.0 ± 18.8 µM	IC_50_ > 100 µM
**B4**	136.8 ± 1.0 µM	IC_50_ > 100 µM
**B5**	79.9 ± 0.7 µM	IC_50_ > 100 µM
**B6**	177.0 ± 5.1 µM	IC_50_ > 100 µM
**B7**	256.4 ± 63.8 µM	IC_50_ > 100 µM
**B8**	132.7 ± 43.2 µM	IC_50_ > 100 µM
**B9**	204.1 ± 28.5 µM	IC_50_ > 100 µM
**B10**	103.9 ± 1.0 µM	134.7 ± 9.3 µM

**Table 3:** Primer sequences used for RT-qPCR analysis.

Gene	Primer Forward	Primer Reverse
*BCL2*	CTTCAGGGACGGGGTGAAC	GGATCCAGGTGTGCAGGTG
*MDM2*	GCCCTTCGTGAGAATTGGCT	CCTCAACACATGACTCTCTGG
*AIFM2*	CTGCCCTTCTCTCATCTTATCCT	CTGCCTCACCATGTCCTCATAG
*IL6*	GTTCTGCCAAACCAGCCTTG	AGATCACCTAGTCCACCCCC
*IL8*	GGTGCAGTTTTGCCAAGGAG	ACCAAGGCACAGTGGAACAA
*RPL41*	TCCTGCGTTGGGATTCCGTG	ACGGTGCAACAAGCTAGCGG
*OGG1*	CTGCATCCTGCCTGGAGTGG	CCTGACAGCACCGCTTGG

## Data Availability

Data are contained within the article and Supplementary Materials.

## Supplementary Materials

The following supporting information can be downloaded at https://www.mdpi.com/article/10.3390/ph19081133/s1: ^1^H NMR and ^13^C NMR spectra and HR MS of compounds B1 –B10; Figure S1. Dose–response curves of compounds B1–B10 in HCT116 and BEAS-2B cells used for IC_50_ determination; Figure S2. Representative cell cycle histograms obtained by flow cytometry; Figure S3. Representative Annexin V-FITC/PI dot plots obtained by flow cytometry; Figure S4. Representative ROS histograms obtained by flow cytometry.

## Abbreviations

The following abbreviations are used in this manuscript:

AIFM2	Apoptosis-Inducing Factor Mitochondria-Associated 2
AKT	Protein Kinase B
BAX	BCL2-Associated X Protein
BCL2	B-Cell Lymphoma 2
CA IX	Carbonic Anhydrase IX
CuAAC	Copper-Catalyzed Azide–Alkyne Cycloaddition
DMEM	Dulbecco’s Modified Eagle Medium
DNA	Deoxyribonucleic Acid
DR5	Death Receptor 5
EGFR	Epidermal Growth Factor Receptor
EMT	Epithelial–Mesenchymal Transition
FBS	Fetal Bovine Serum
FSP1	Ferroptosis Suppressor Protein 1
GI50	Growth Inhibition 50
GM-CSF	Granulocyte–Macrophage Colony-Stimulating Factor
HDAC	Histone Deacetylase
HMGCS2	3-Hydroxy-3-Methylglutaryl-CoA Synthase 2
HRMS	High-Resolution Mass Spectrometry
IC_50_	Half Maximal Inhibitory Concentration
IDO1	Indoleamine 2,3-Dioxygenase 1
IFNβ	Interferon Beta
IL6	Interleukin 6
IL8	Interleukin 8
IL-12p40	Interleukin 12 p40 Subunit
JAK	Janus Kinase
KIT	KIT Proto-Oncogene Receptor Tyrosine Kinase
LPS	Lipopolysaccharide
MAPK	Mitogen-Activated Protein Kinase
MDM2	Mouse Double Minute 2 Homolog
miRNA	MicroRNA
NCI-60	National Cancer Institute 60 Human Tumor Cell Line Screen
NMR	Nuclear Magnetic Resonance
OGG1	8-Oxoguanine DNA Glycosylase
PARP	Poly(ADP-Ribose) Polymerase
PBS	Phosphate-Buffered Saline
PI	Propidium Iodide
PI3K	Phosphoinositide 3-Kinase
PIK3CA	Phosphatidylinositol-4,5-Bisphosphate 3-Kinase Catalytic Subunit Alpha
qPCR	Quantitative Polymerase Chain Reaction
RNA	Ribonucleic Acid
RNase	Ribonuclease
ROS	Reactive Oxygen Species
RPMI	Roswell Park Memorial Institute Medium
RT-qPCR	Real-Time Quantitative Polymerase Chain Reaction
SAR	Structure-Activity Relationship
TNF-α	Tumor Necrosis Factor Alpha
Topo	Topoisomerase
