# Supplementary material for: Novel Triazole–Diquinothiazine Hybrids Modulating Apoptosis and Stress Pathways in Colorectal Cancer Cells
Source: Pharmaceuticals (Basel). 2026 Jul 23;19(8):1133. doi: 10.3390/ph19081133 (PMC13516452; doi:10.3390/ph19081133)
Supplement: Supplementary file 1 [file pharmaceuticals-19-01133-s001.zip › pharmaceuticals-4417388-supplementary.pdf]

Novel Triazole-Diquinothiazine Hybrids Modulating Apoptosis and Stress Pathways in Colorectal Cancer Cells

Klaudia Giercuszkiewicz-Haśnik [1,2,*], Magdalena Skonieczna [1,2], Beata Morak-Młodawska [3] and Małgorzata Jeleń [3,*]

[1] Department of Systems Biology and Engineering, The Silesian University of Technology, Akademicka Street 16, 44-100 Gliwice, Poland; klaudia.giercuszkiewicz@polsl.pl (K.G.-H.); magdalena.skonieczna@polsl.pl (M.S.)

[2] Centre of Biotechnology, Silesian University of Technology, Krzywoustego Street 8, 44-100 Gliwice, Poland

[3] Department of Organic Chemistry, Faculty of Pharmaceutical Sciences in Sosnowiec, Medical University of Silesia in Katowice, Jagiellońska Street 4, 41-200 Sosnowiec, Poland; manowak@sum.edu.pl (M.J.), bmlodawska@sum.edu.pl (B.M.-M.)

* Correspondence: klaudia.giercuszkiewicz@polsl.pl (K.G.-H.), manowak@sum.edu.pl (M.J.)

Content:

^{1}H NMR and ^{13}C NMR spectra and HR MS of compounds **B1-B10**.

Figure S1. Dose–response curves of compounds B1–B10 in HCT116 and BEAS-2B cells used for IC_{50} determination.

Figure S2. Representative cell cycle histograms obtained by flow cytometry.

Figure S3. Representative Annexin V-FITC/PI dot plots obtained by flow cytometry.

Figure S4. Representative ROS histograms obtained by flow cytometry.

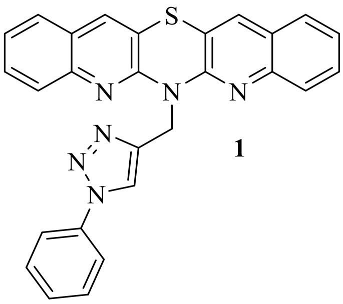

1

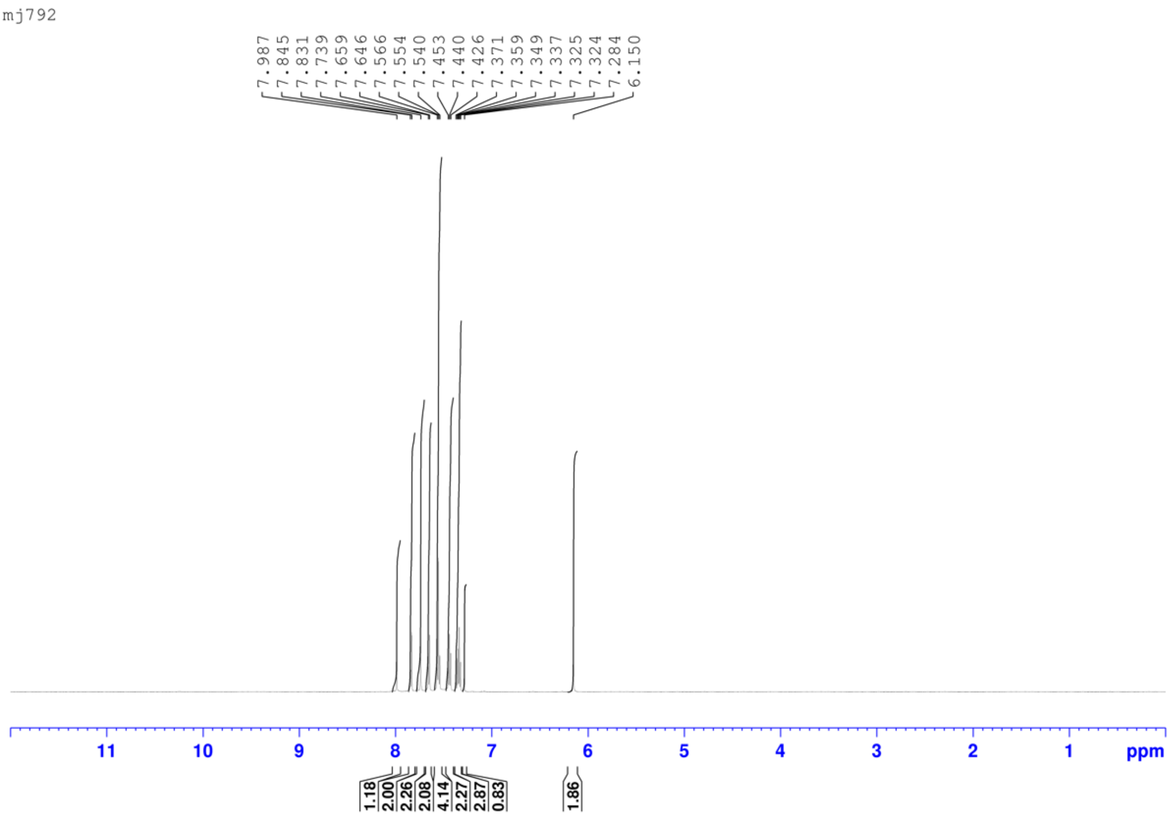
mj792
7.987
7.845
7.831
7.739
7.659
7.646
7.566
7.554
7.540
7.453
7.440
7.426
7.371
7.359
7.349
7.337
7.325
7.324
7.284
6.150
11 10 9 8 7 6 5 4 3 2 1 ppm
1.18
2.00
2.26
2.08
4.14
2.27
2.87
0.83
1.86

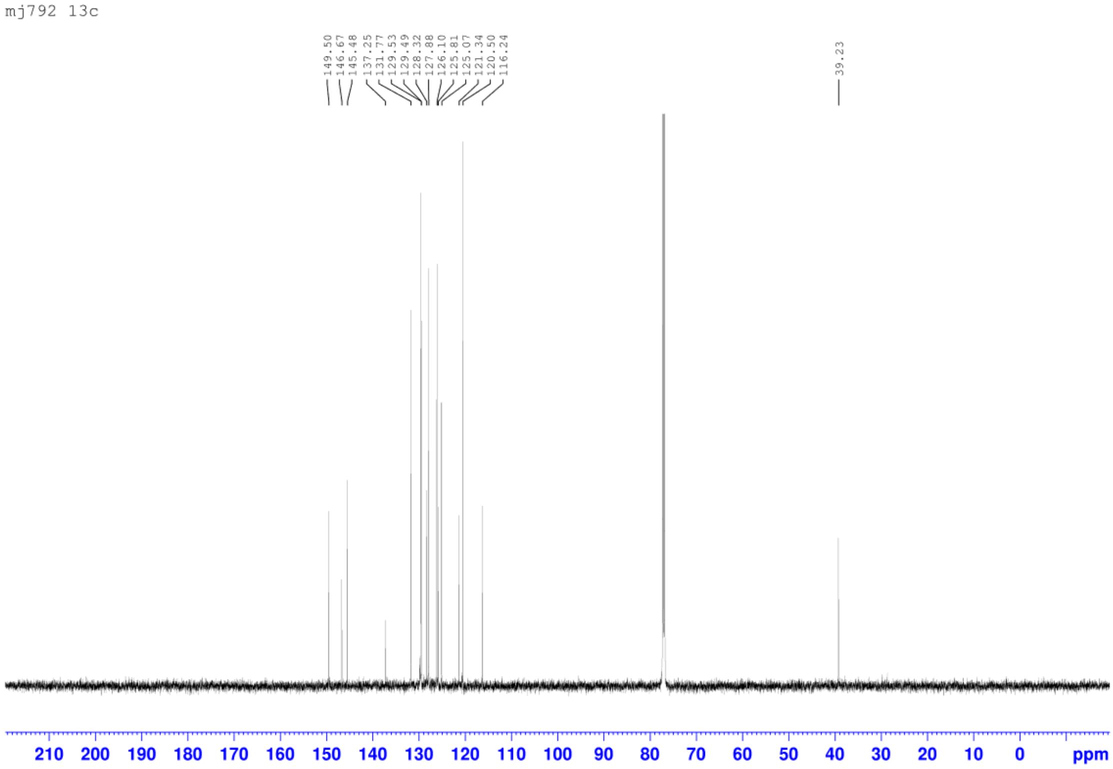

Source Type	ESI	Ion Polarity	Positive	Set Nebulizer	0.3 Bar
Focus	Not active	Set Capillary	4000 V	Set Dry Heater	200 °C
Scan Begin	100 m/z	Set End Plate Offset	-500 V	Set Dry Gas	3.0 l/min
Scan End	1000 m/z	Set Charging Voltage	2000 V	Set Divert Valve	Source
		Set Corona	0 nA	Set APCI Heater	0 °C

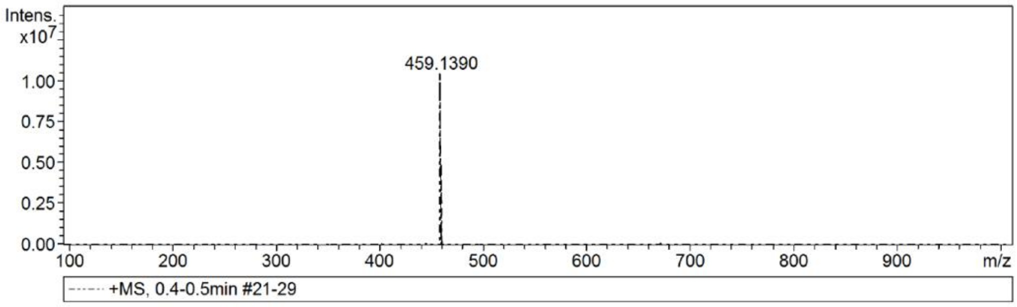

#	m/z	Res.	S/N	I	I %	FWHM
1	459.1390	11947	85831.1	10188798	100.0	0.0285

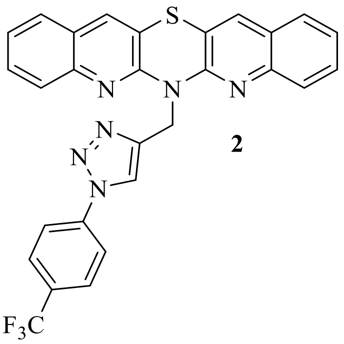
S
N
N
N
N
N
N
F3C

2

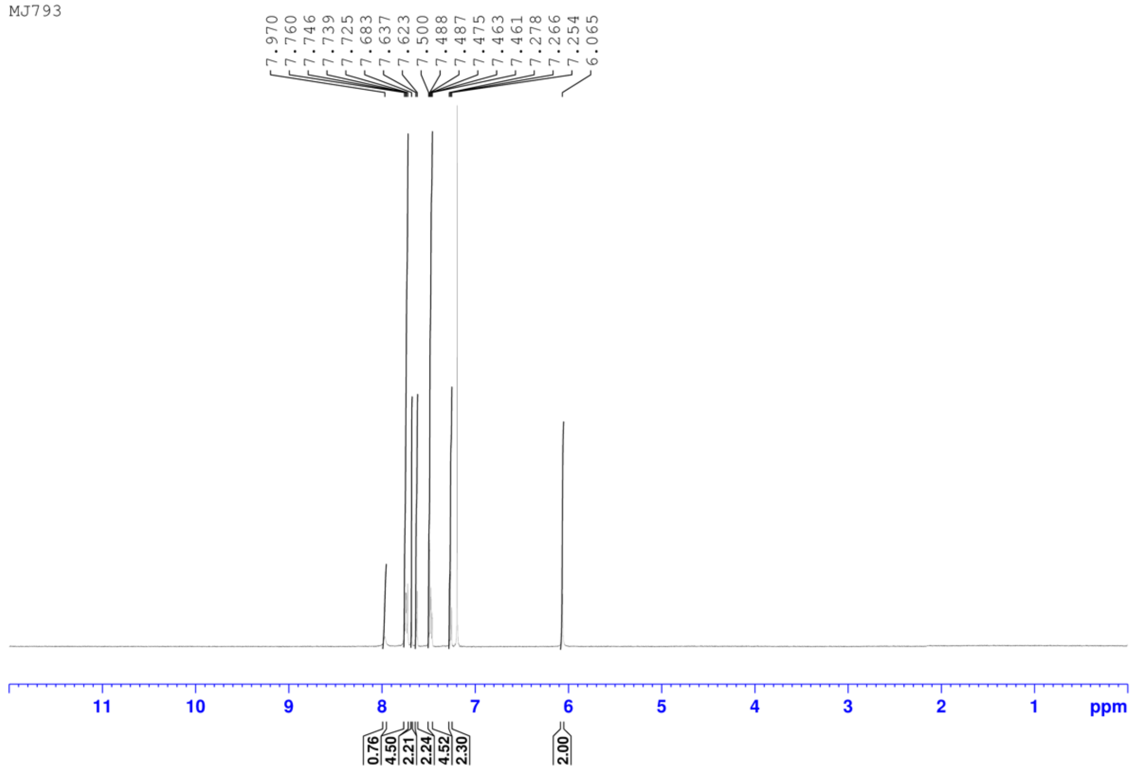
MJ793
7.970
7.760
7.746
7.739
7.725
7.683
7.637
7.623
7.500
7.488
7.487
7.475
7.463
7.461
7.278
7.266
7.254
6.065
11
10
9
8
7
6
5
4
3
2
1
ppm
0.76
4.50
2.21
2.24
4.52
2.30
2.00

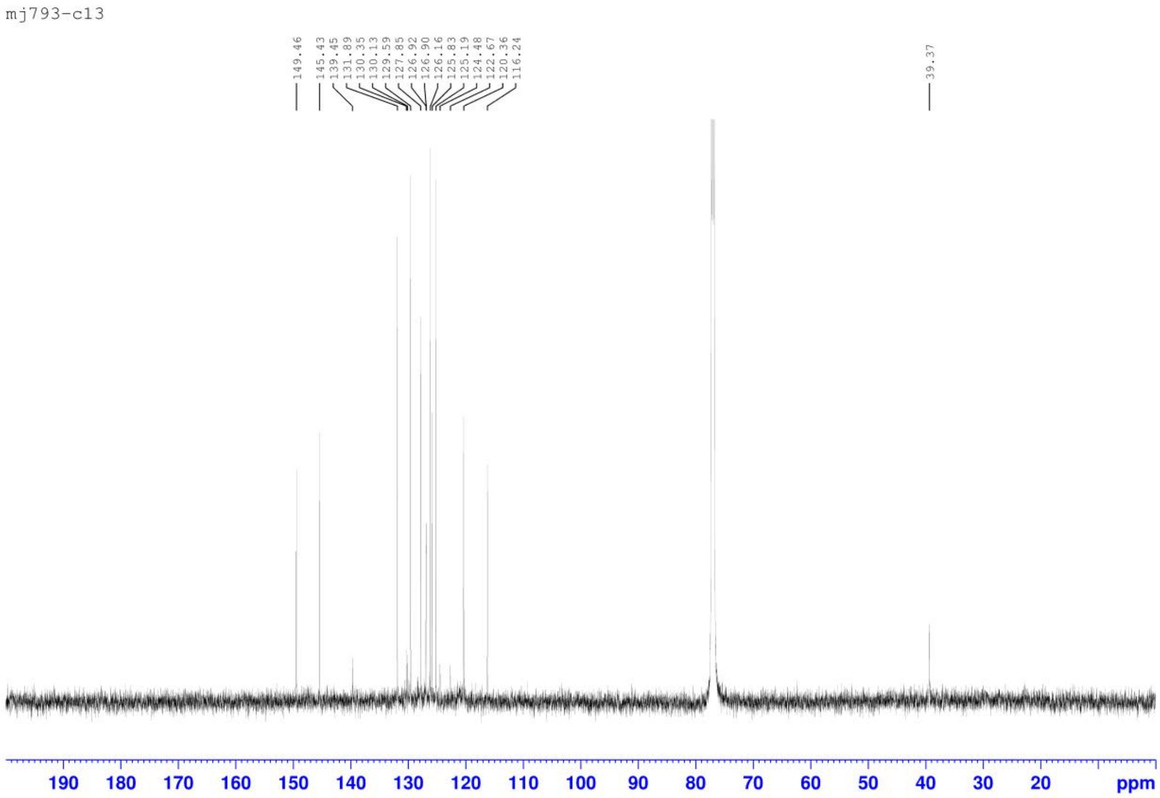

Acquisition Parameter

Source Type	ESI	Ion Polarity	Positive	Set Nebulizer	0.4 Bar
Focus	Active	Set Capillary	4500 V	Set Dry Heater	180 °C
Scan Begin	50 m/z	Set End Plate Offset	-500 V	Set Dry Gas	4.0 l/min
Scan End	1000 m/z	Set Charging Voltage	2000 V	Set Divert Valve	Source
		Set Corona	0 nA	Set APCI Heater	0 °C

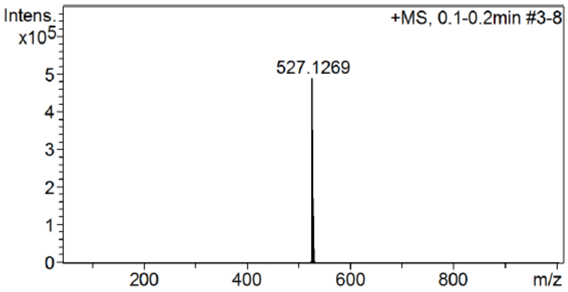

#	m/z	Res.	S/N	I	I %	FWHM
1	527.1269	22764	2632.4	488294	100.0	0.0232

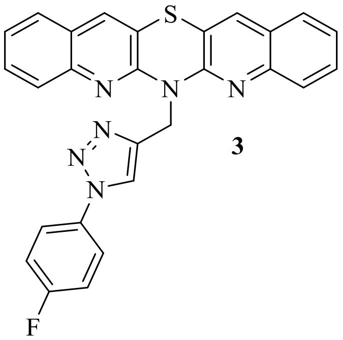

3

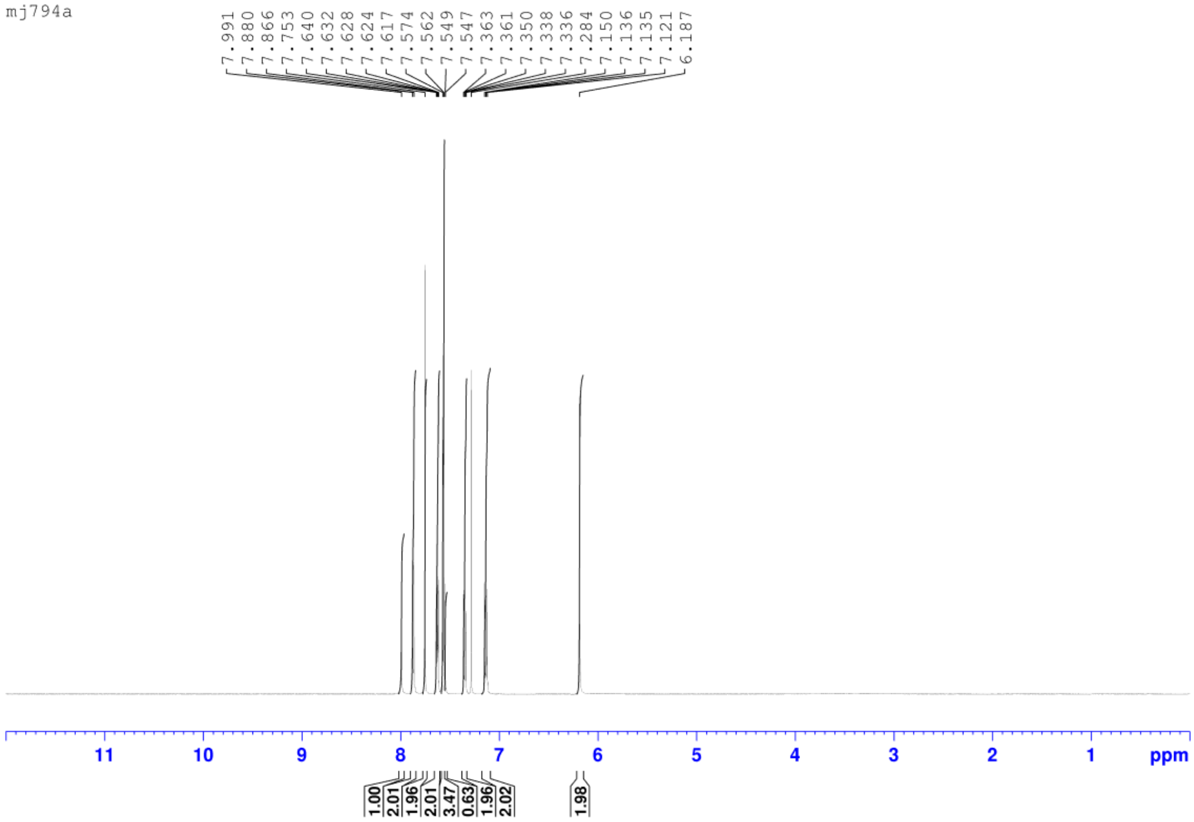

mj794a
7.991
7.880
7.866
7.753
7.640
7.632
7.628
7.624
7.617
7.574
7.562
7.549
7.547
7.363
7.361
7.350
7.338
7.336
7.284
7.150
7.136
7.135
7.121
6.187
11
10
9
8
7
6
5
4
3
2
1
ppm
1.00
2.01
1.96
2.01
3.47
0.63
1.96
2.02
1.98

mj794a 13c

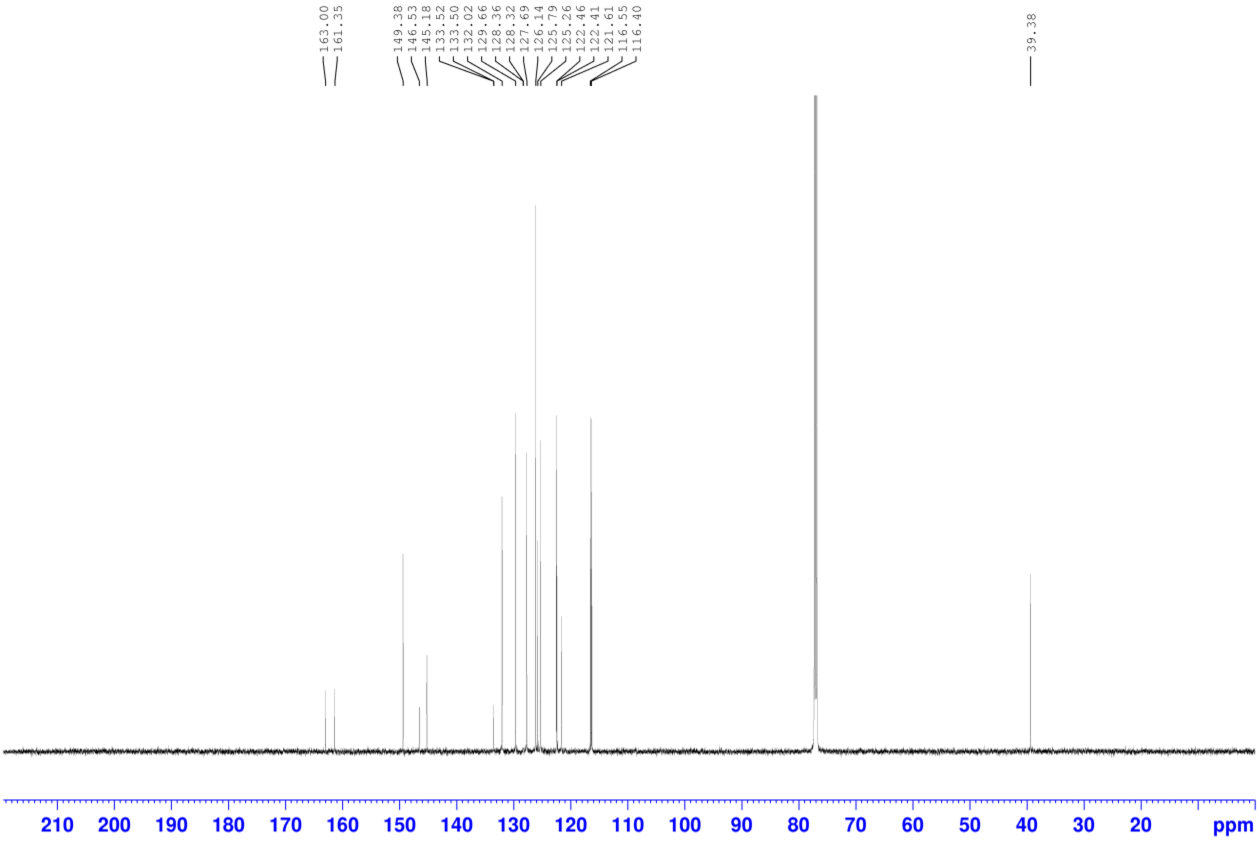

Acquisition Parameter

Source Type	ESI	Ion Polarity	Positive	Set Nebulizer	0.4 Bar
Focus	Active	Set Capillary	4500 V	Set Dry Heater	180 °C
Scan Begin	50 m/z	Set End Plate Offset	-500 V	Set Dry Gas	4.0 l/min
Scan End	1000 m/z	Set Charging Voltage	2000 V	Set Divert Valve	Source
		Set Corona	0 nA	Set APCI Heater	0 °C

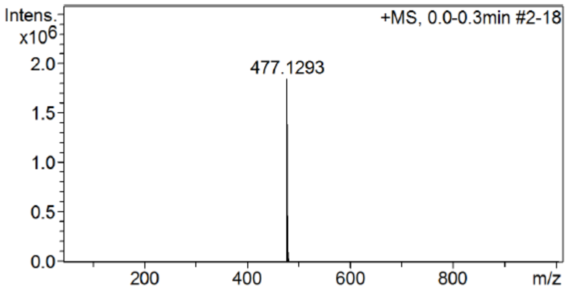

#	m/z	Res.	S/N	I	I %	FWHM
1	477.1293	32254	25399.4	1850944	100.0	0.0148

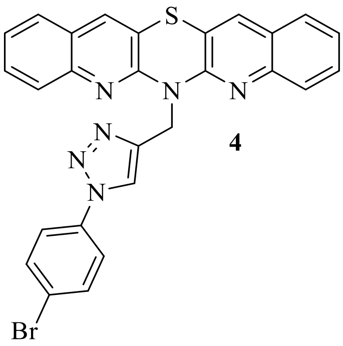

4

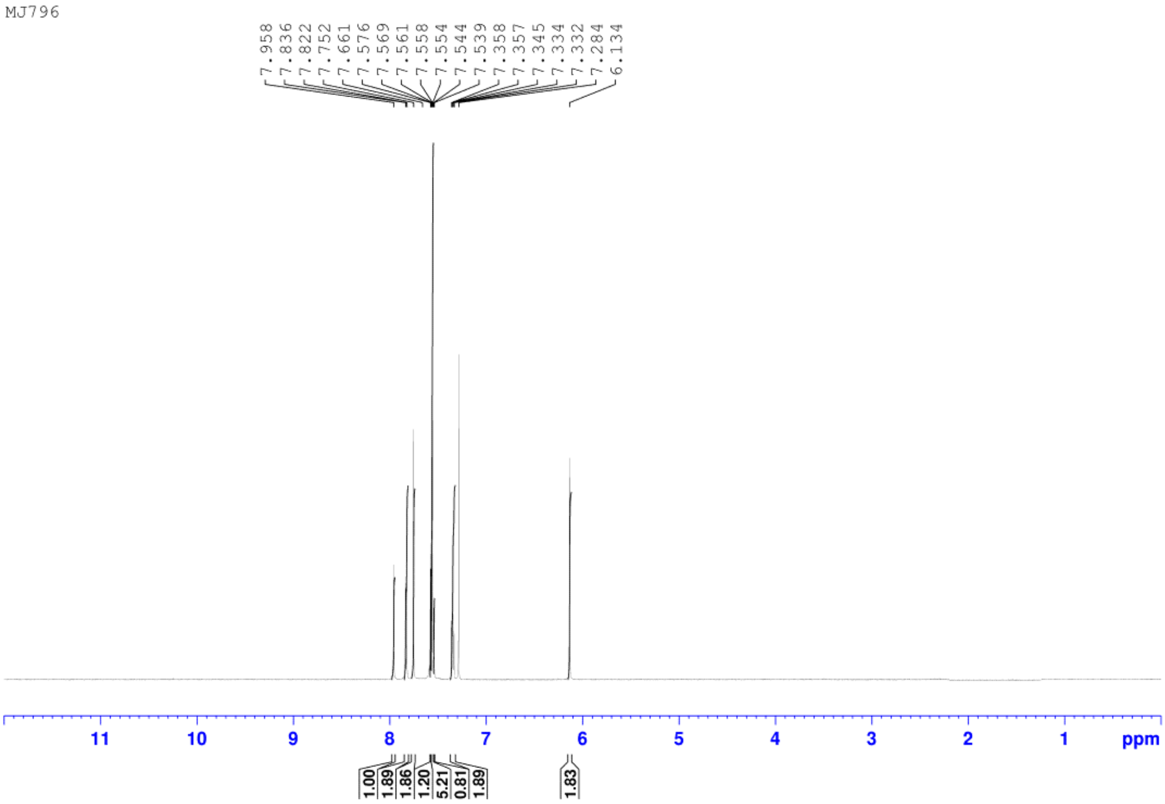

MJ796
7.958
7.836
7.822
7.752
7.661
7.576
7.569
7.561
7.558
7.554
7.544
7.539
7.358
7.357
7.345
7.334
7.332
7.284
6.134
11 10 9 8 7 6 5 4 3 2 1 ppm
1.00
1.89
1.86
1.20
5.21
0.81
1.89
1.83

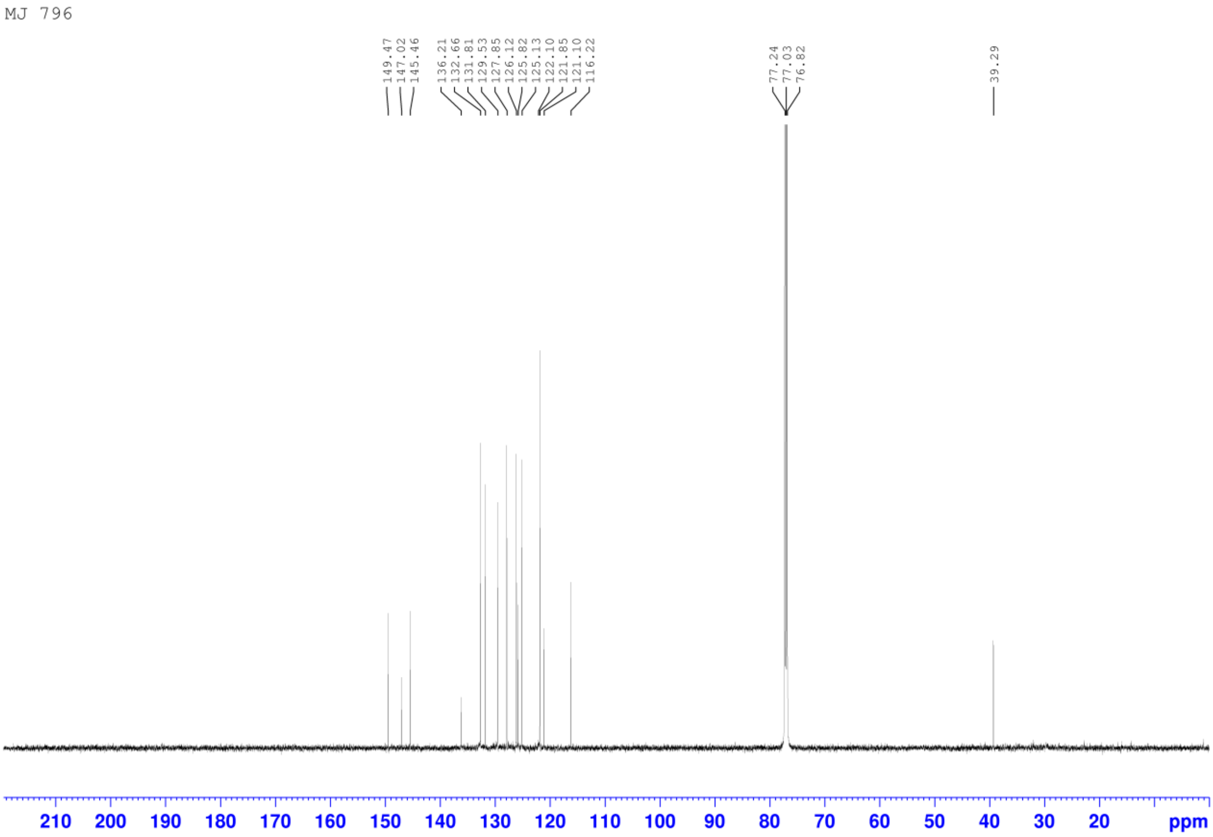

Acquisition Parameter

Source Type	ESI	Ion Polarity	Positive	Set Nebulizer	0.4 Bar
Focus	Active	Set Capillary	4500 V	Set Dry Heater	180 °C
Scan Begin	50 m/z	Set End Plate Offset	-500 V	Set Dry Gas	4.0 l/min
Scan End	1000 m/z	Set Charging Voltage	2000 V	Set Divert Valve	Source
		Set Corona	0 nA	Set APCI Heater	0 °C

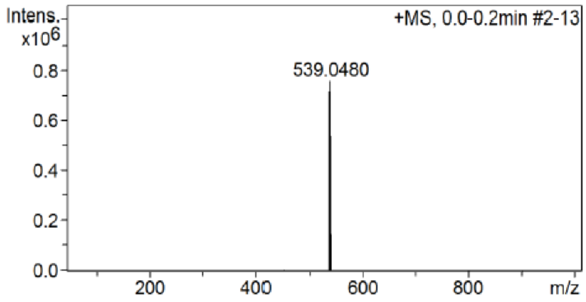

#	m/z	Res.	S/N	I	I %	FWHM
1	537.0498	25810	6572.1	716492	94.6	0.0208
2	539.0480	25750	6912.2	757625	100.0	0.0209

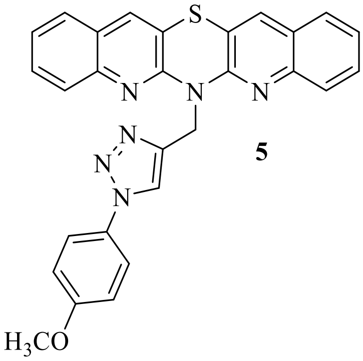

5

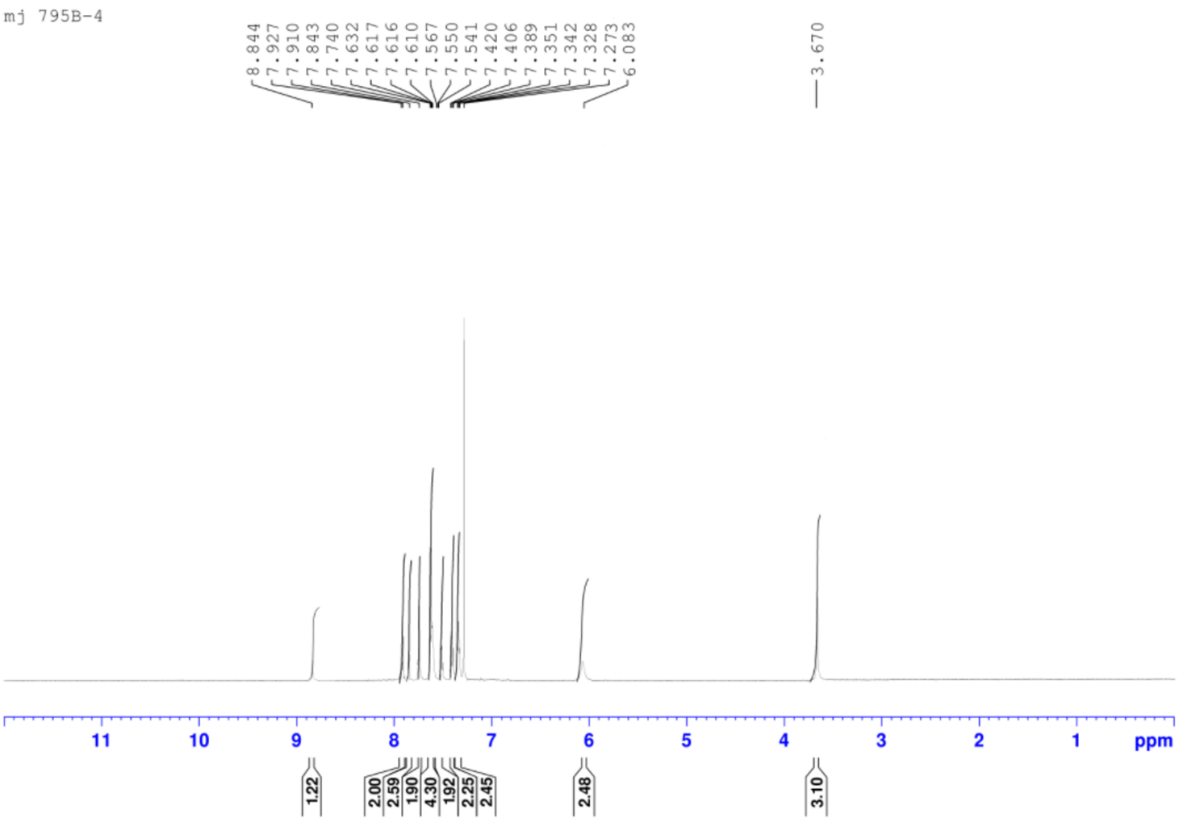

mj 795B-4
8.844
7.927
7.910
7.843
7.740
7.632
7.617
7.616
7.610
7.567
7.550
7.541
7.420
7.406
7.389
7.351
7.342
7.328
7.273
6.083
3.670
11
10
9
8
7
6
5
4
3
2
1
ppm
1.22
2.00
2.59
1.90
4.30
1.92
2.25
2.45
2.48
3.10

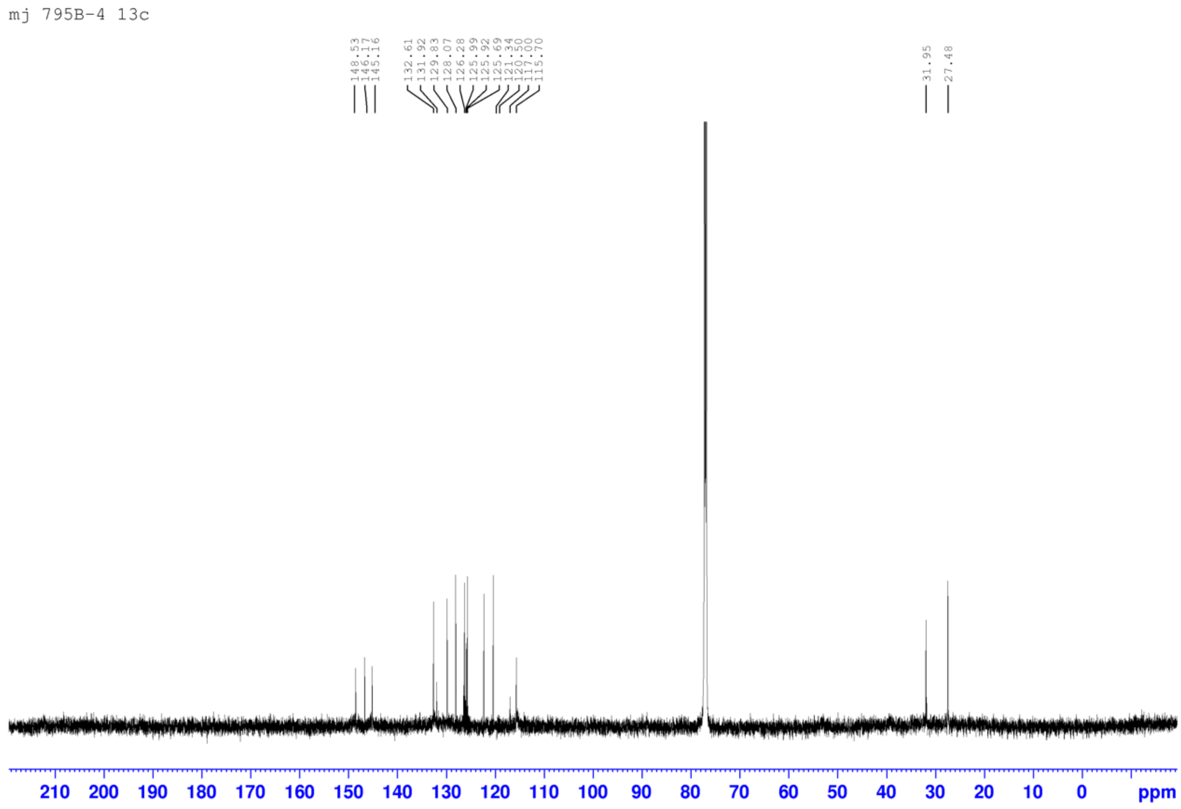

Acquisition Parameter

Source Type	ESI	Ion Polarity	Positive	Set Nebulizer	0.4 Bar
Focus	Active	Set Capillary	4500 V	Set Dry Heater	180 °C
Scan Begin	50 m/z	Set End Plate Offset	-500 V	Set Dry Gas	4.0 l/min
Scan End	1000 m/z	Set Charging Voltage	2000 V	Set Divert Valve	Source
		Set Corona	0 nA	Set APCI Heater	0 °C

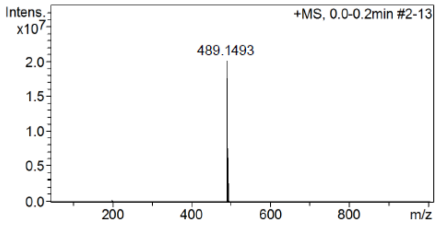

#	m/z	Res.	S/N	I	I %	FWHM
1	489.1493	19250	447747.7	20147808	100.0	0.0201

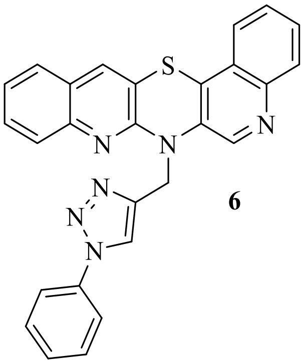

6

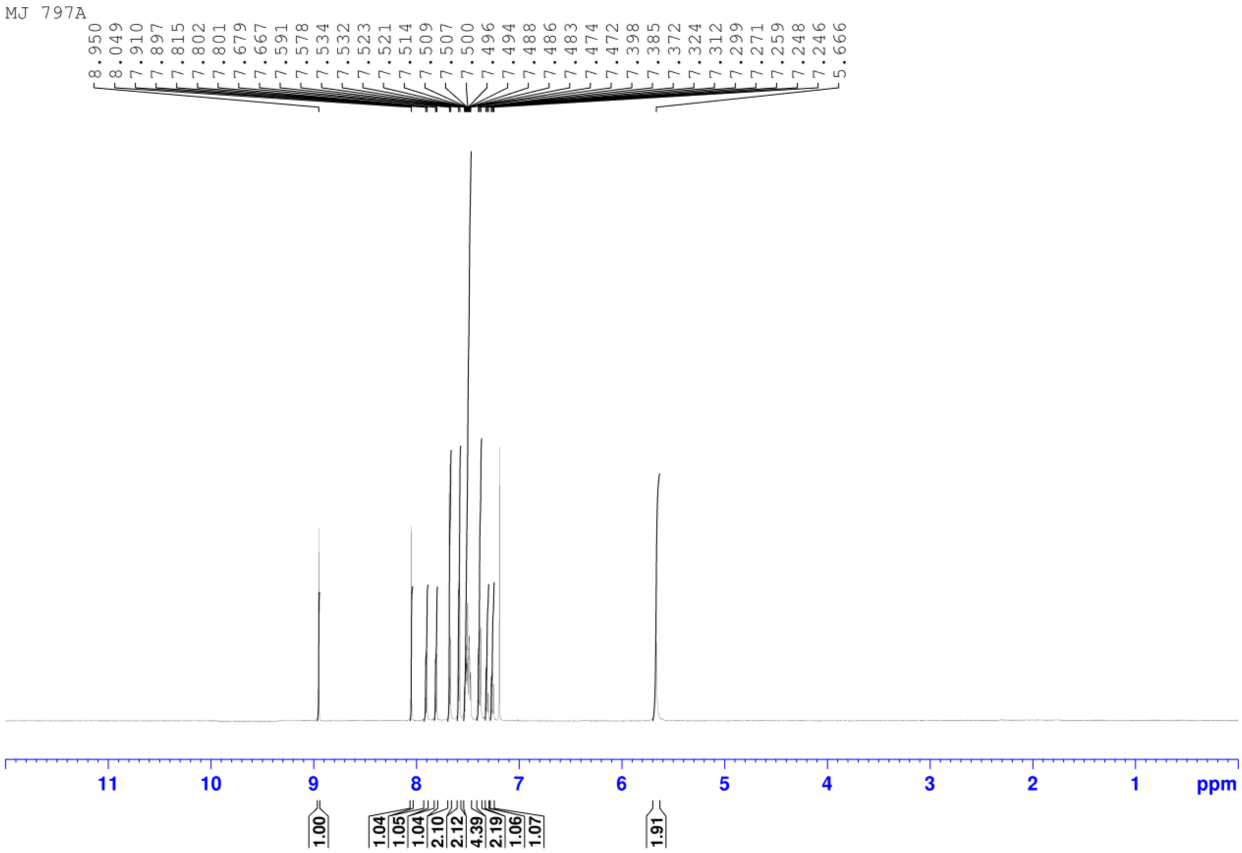
MJ 797A
8.950
8.049
7.910
7.897
7.815
7.802
7.801
7.679
7.667
7.591
7.578
7.534
7.532
7.523
7.521
7.514
7.509
7.507
7.500
7.496
7.494
7.488
7.486
7.483
7.474
7.472
7.398
7.385
7.372
7.324
7.312
7.299
7.271
7.259
7.248
7.246
5.666
11
10
9
8
7
6
5
4
3
2
1
ppm
1.00
1.04
1.05
1.04
2.10
2.12
4.39
2.19
1.06
1.07
1.91

MJ 797A

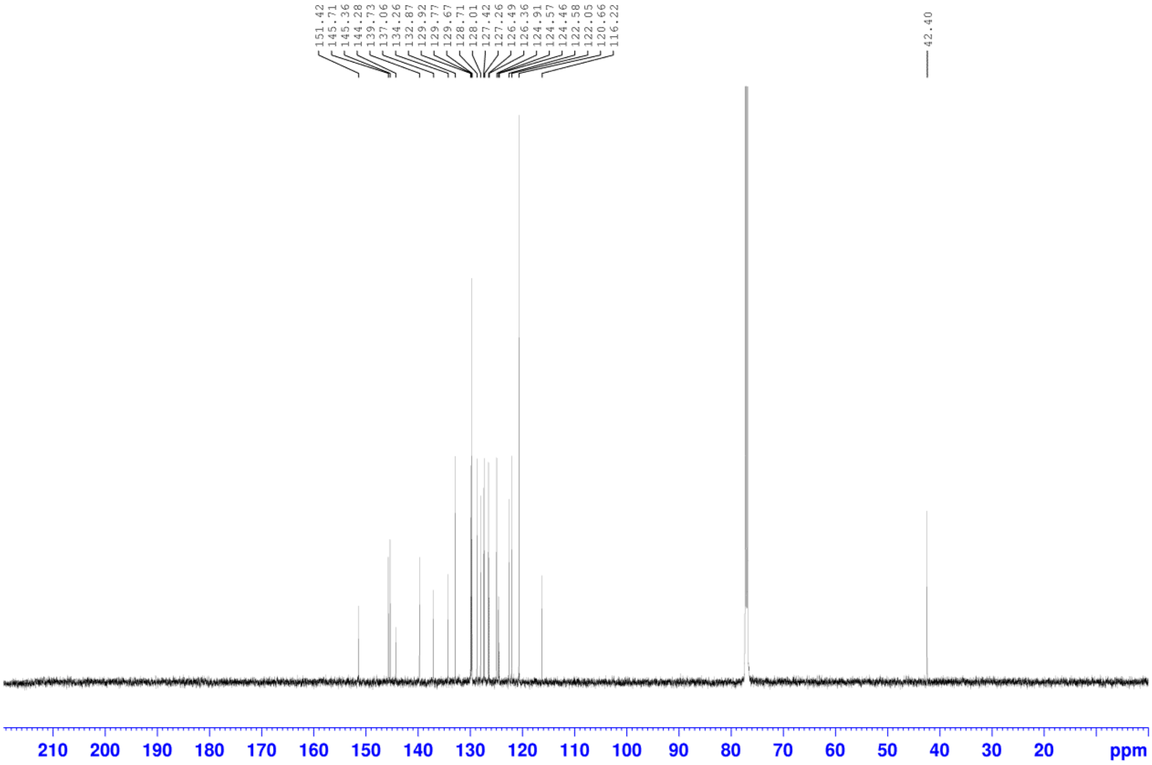

Acquisition Parameter

Source Type	ESI	Ion Polarity	Positive	Set Nebulizer	0.4 Bar
Focus	Active	Set Capillary	4500 V	Set Dry Heater	180 °C
Scan Begin	50 m/z	Set End Plate Offset	-500 V	Set Dry Gas	4.0 l/min
Scan End	1000 m/z	Set Charging Voltage	2000 V	Set Divert Valve	Source
		Set Corona	0 nA	Set APCI Heater	0 °C

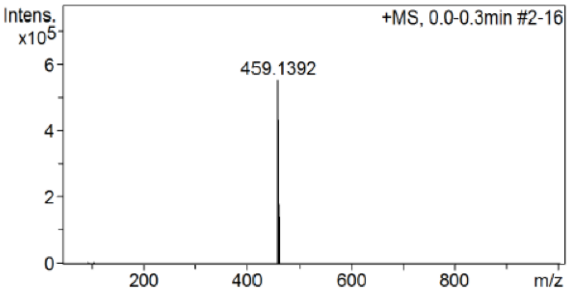

#	m/z	Res.	S/N	I	I %	FWHM
1	459.1392	24086	6074.3	555102	100.0	0.0191

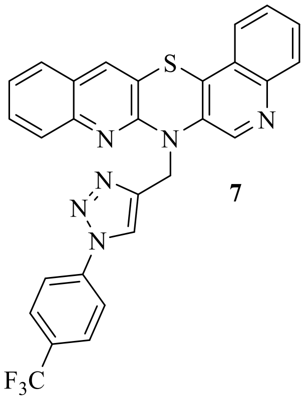
S
N
N
N
N
N
N
F3C

7

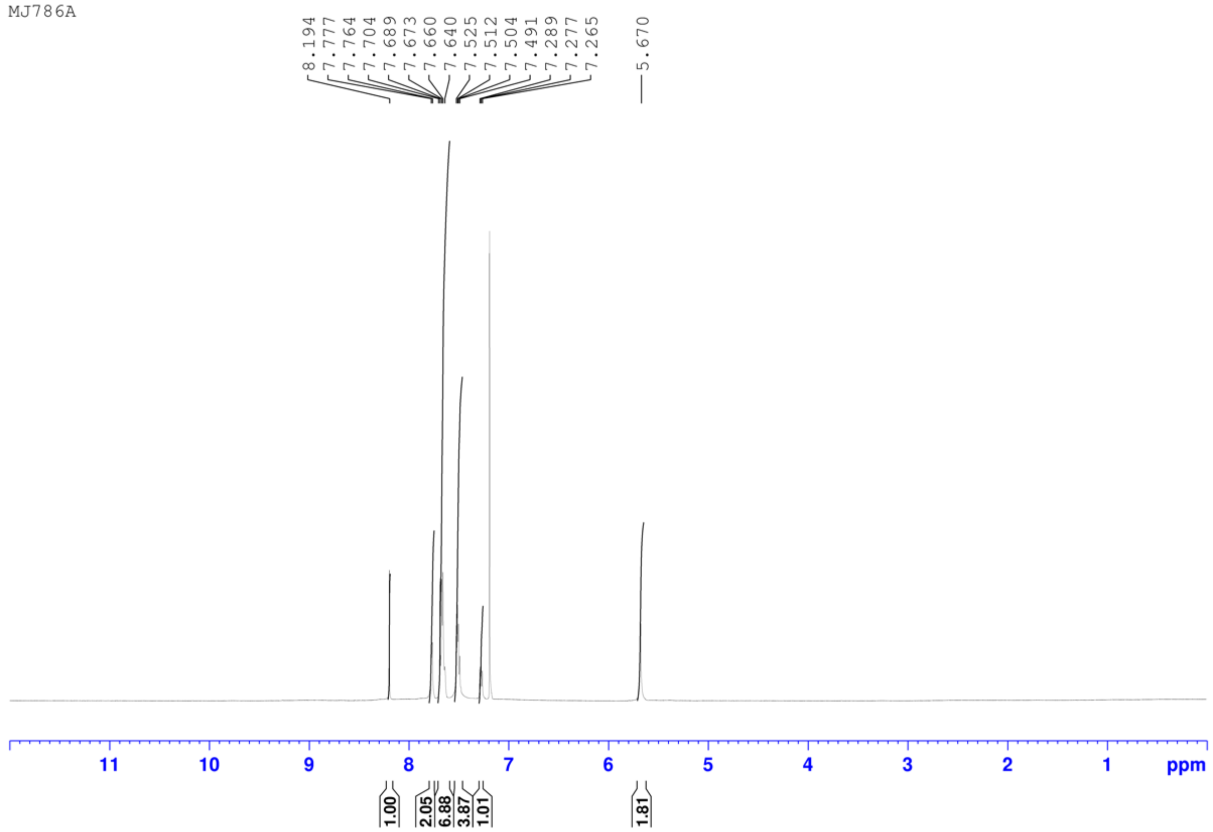
MJ786A
8.194
7.777
7.764
7.704
7.689
7.673
7.660
7.640
7.525
7.512
7.504
7.491
7.289
7.277
7.265
5.670
11
10
9
8
7
6
5
4
3
2
1
ppm
1.00
2.05
6.88
3.87
1.01
1.81

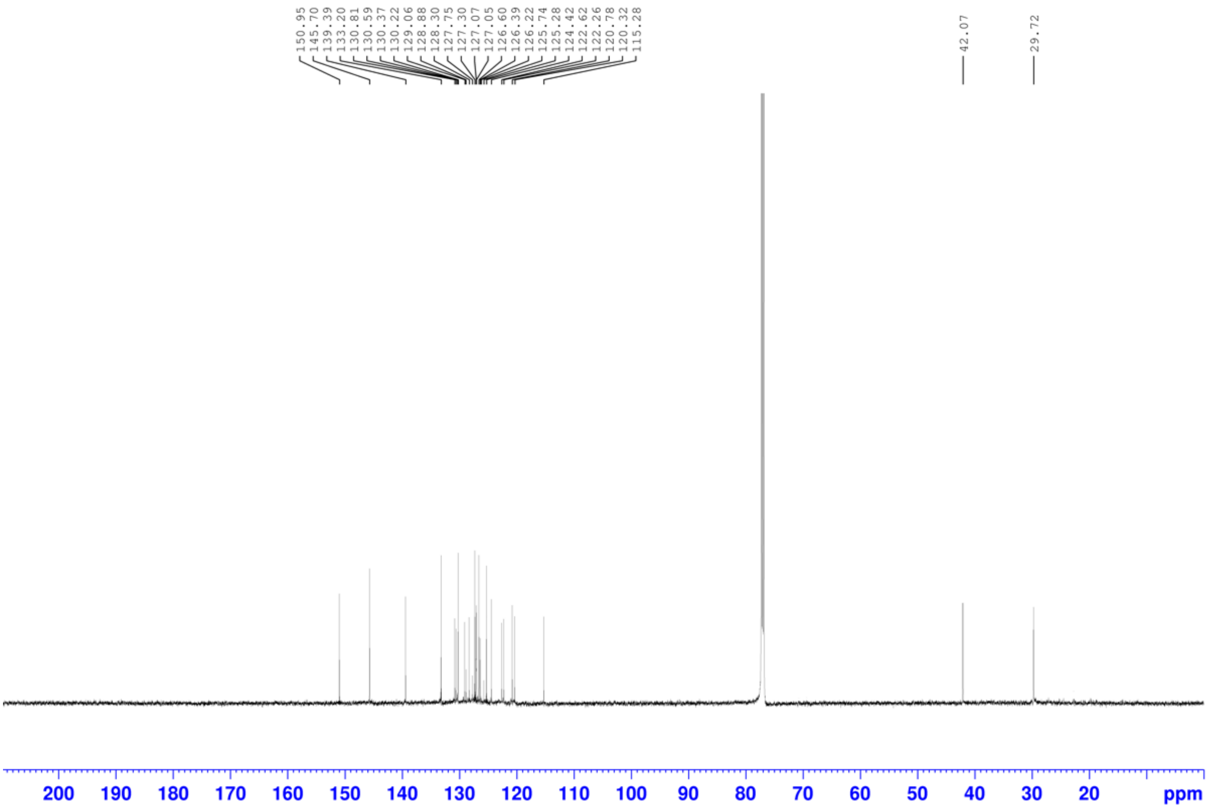

+MS, 0.1-0.4min #3-26

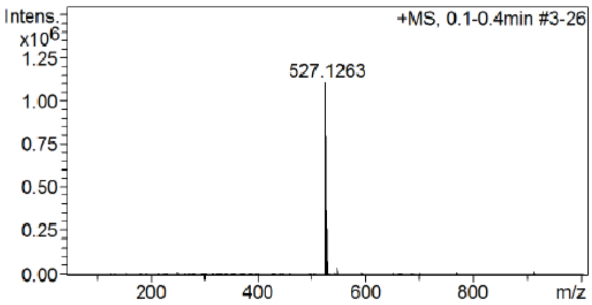

#	m/z	Res.	S/N	I	I %	FWHM
1	527.1263	27943	10388.8	1108426	100.0	0.0189

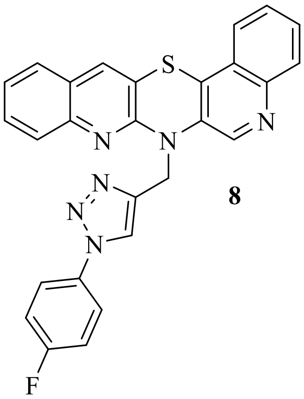

8

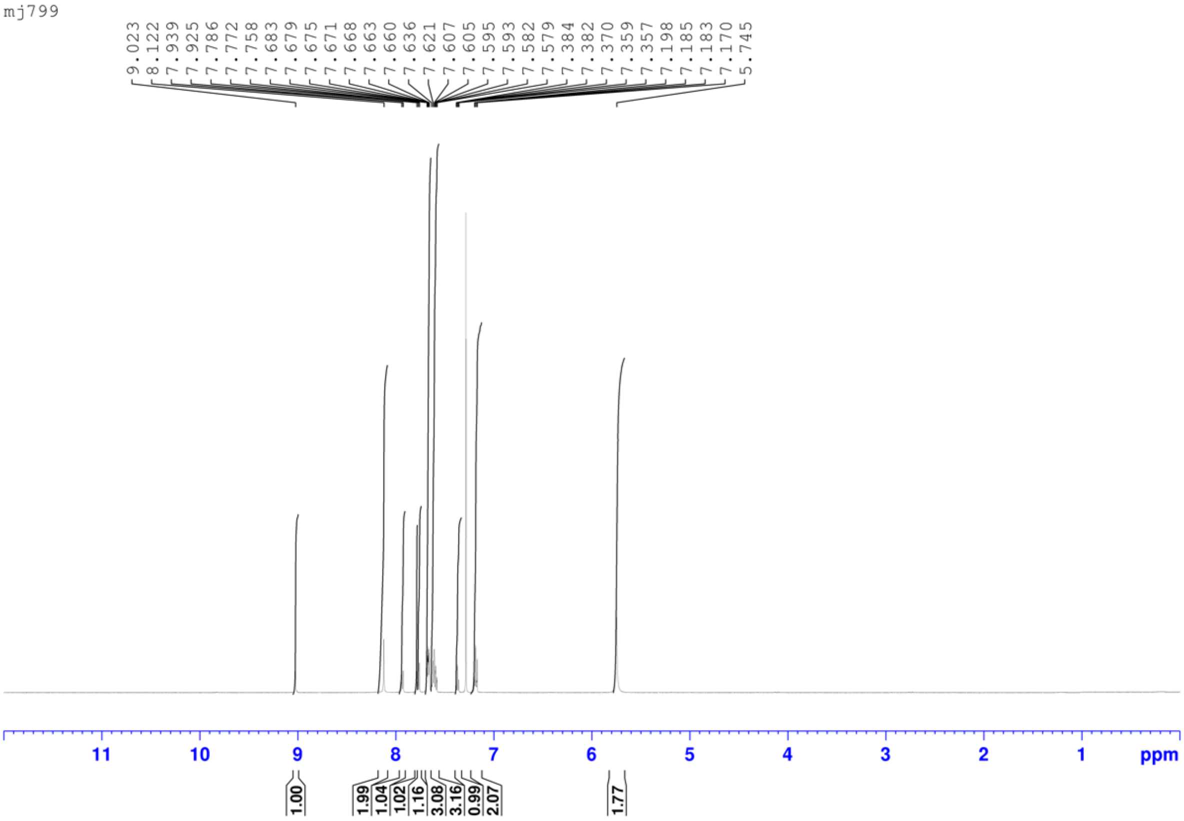
mj799
9.023
8.122
7.939
7.925
7.786
7.772
7.758
7.683
7.679
7.675
7.671
7.668
7.663
7.660
7.636
7.621
7.607
7.605
7.595
7.593
7.582
7.579
7.384
7.382
7.370
7.359
7.357
7.198
7.185
7.183
7.170
5.745
11
10
9
8
7
6
5
4
3
2
1
ppm
1.00
1.99
1.04
1.02
1.16
3.08
3.16
0.99
2.07
1.77

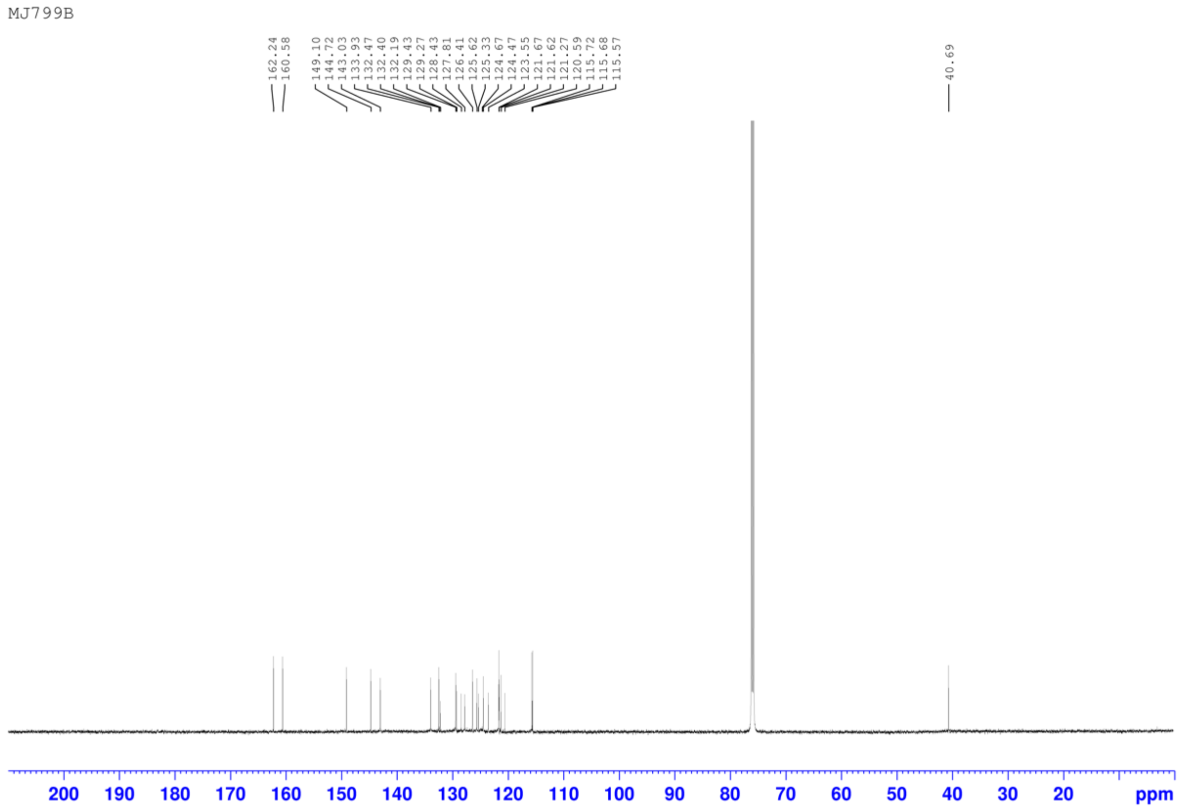

+MS, 0.0-0.2min #2-10

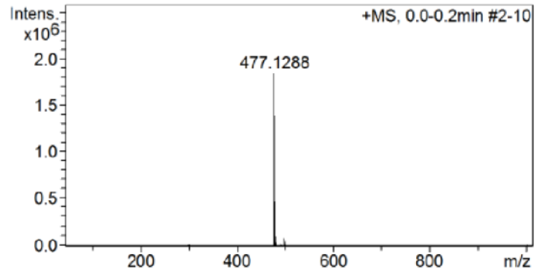

#	m/z	Res.	S/N	I	I %	FWHM
1	477.1288	32754	26669.9	1849050	100.0	0.0146

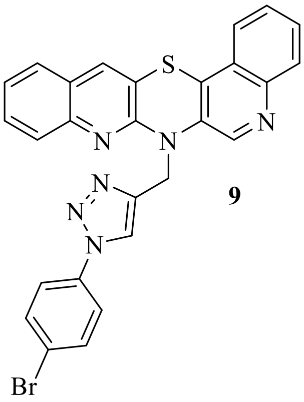

9

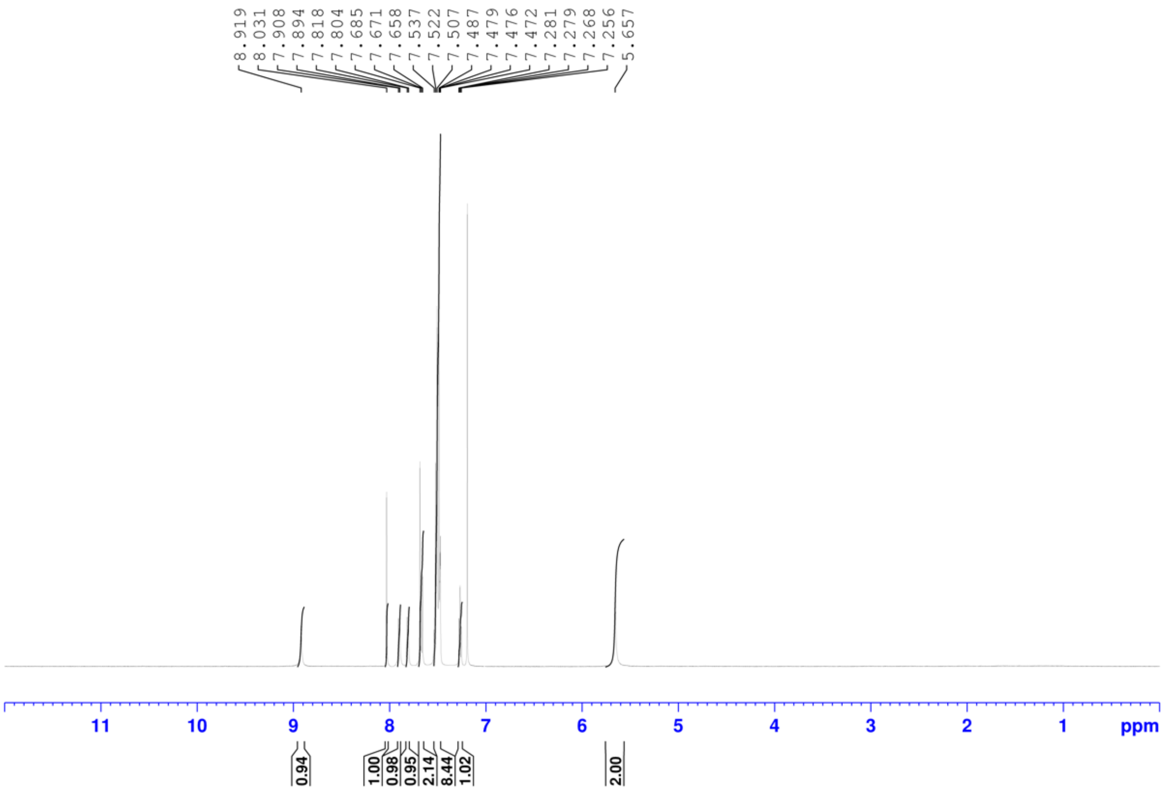
8.919
8.031
7.908
7.894
7.818
7.804
7.685
7.671
7.658
7.537
7.522
7.507
7.487
7.479
7.476
7.472
7.281
7.279
7.268
7.256
5.657
11
10
9
8
7
6
5
4
3
2
1
ppm
0.94
1.00
0.98
0.95
2.14
8.44
1.02
2.00

MJ 802

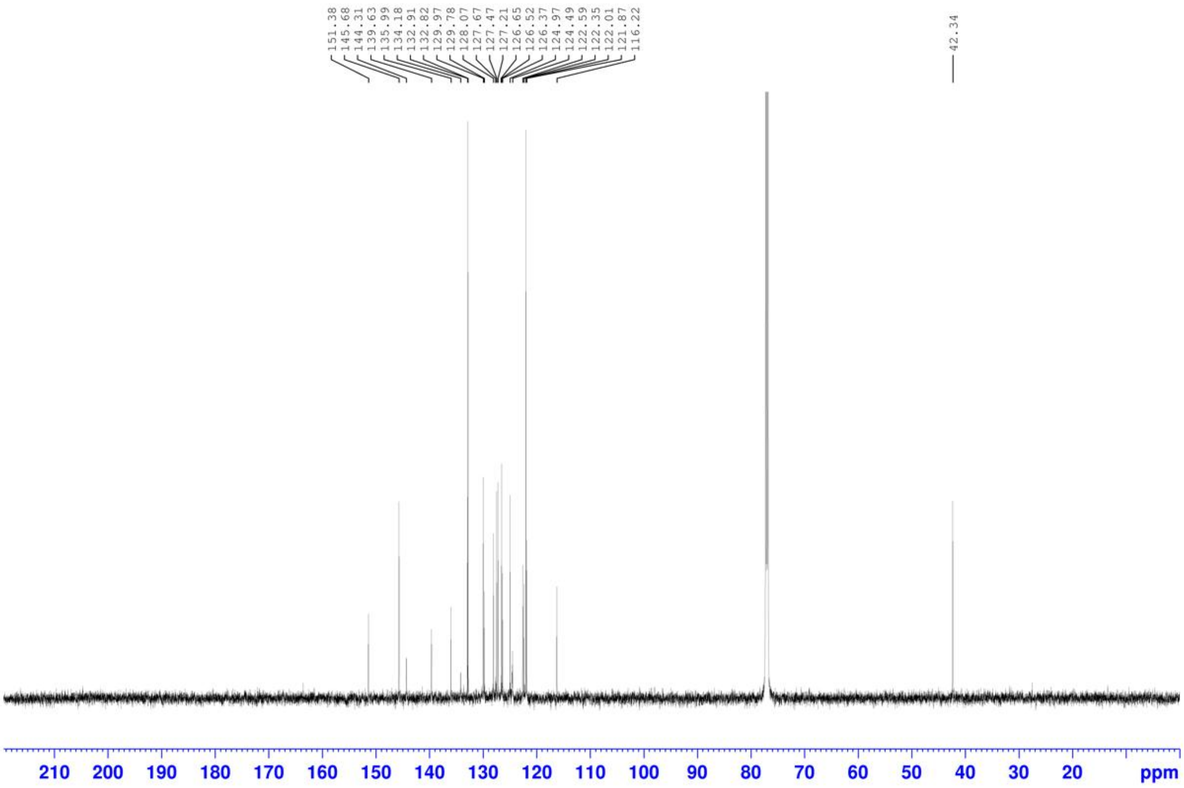

Acquisition Parameter

Source Type	ESI	Ion Polarity	Positive	Set Nebulizer	0.4 Bar
Focus	Active	Set Capillary	4500 V	Set Dry Heater	180 °C
Scan Begin	50 m/z	Set End Plate Offset	-500 V	Set Dry Gas	4.0 l/min
Scan End	1000 m/z	Set Charging Voltage	2000 V	Set Divert Valve	Source
		Set Corona	0 nA	Set APCI Heater	0 °C

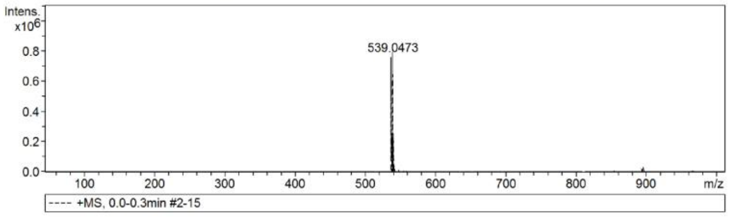

#	m/z	Res.	S/N	I	I %	FWHM
1	537.0491	25713	6341.9	737568	95.5	0.0209
2	539.0473	25637	6609.0	772069	100.0	0.0210

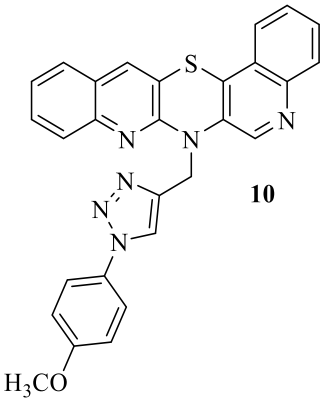

10

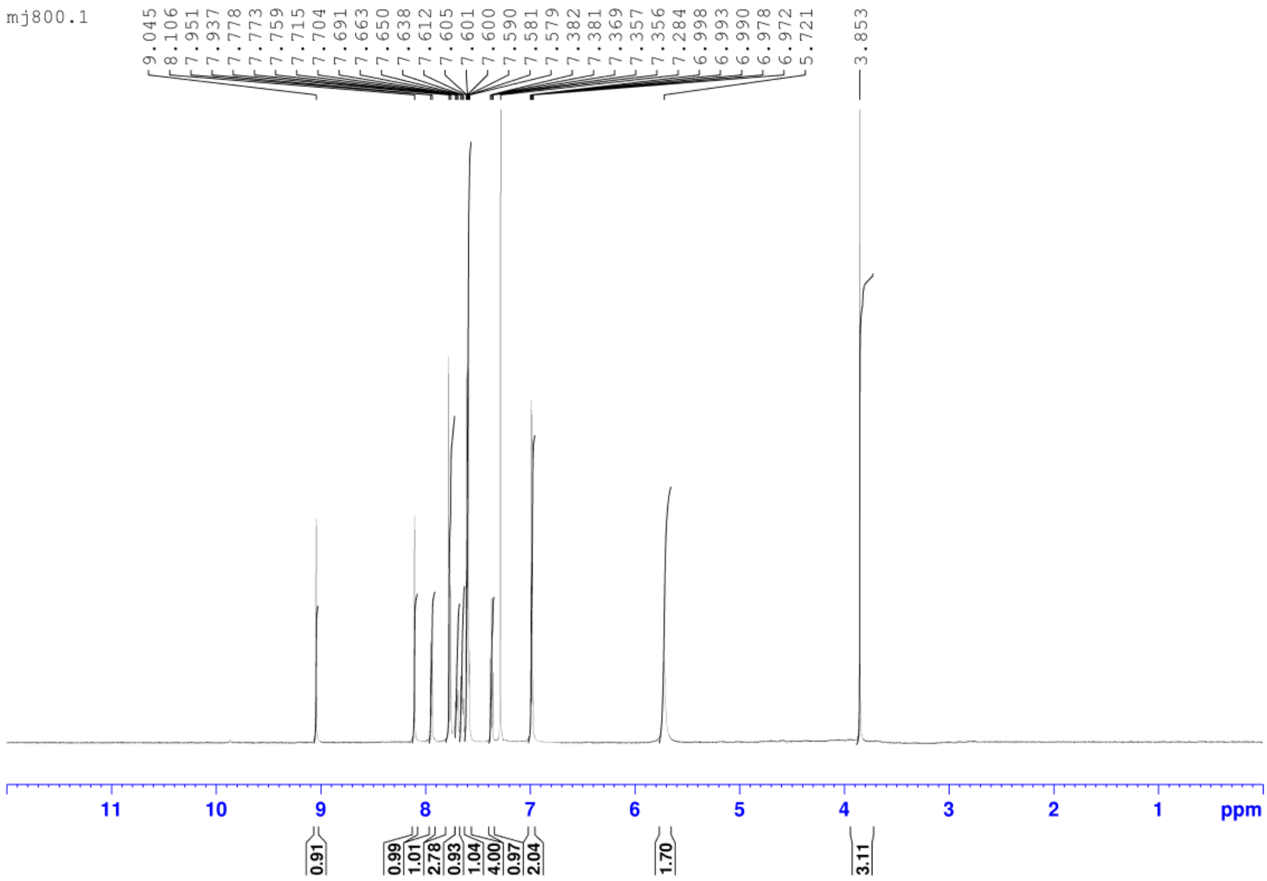
mj800.1
9.045
8.106
7.951
7.937
7.778
7.773
7.759
7.715
7.704
7.691
7.663
7.650
7.638
7.612
7.605
7.601
7.600
7.590
7.581
7.579
7.382
7.381
7.369
7.357
7.356
7.284
6.998
6.993
6.990
6.978
6.972
5.721
3.853
11
10
9
8
7
6
5
4
3
2
1
ppm
0.91
0.99
1.01
2.78
0.93
1.04
4.00
0.97
2.04
1.70
3.11

MJ800B 06.02.2026

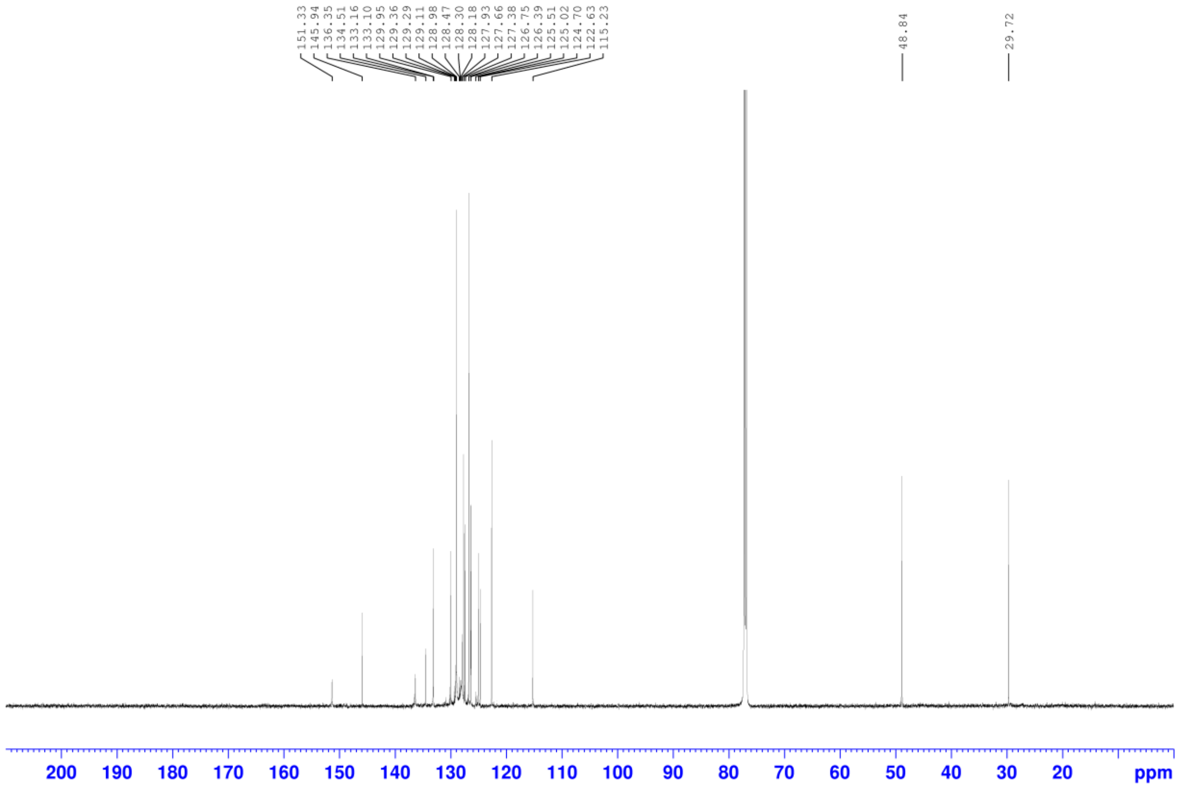

+MS, 0.0-0.2min #2-11

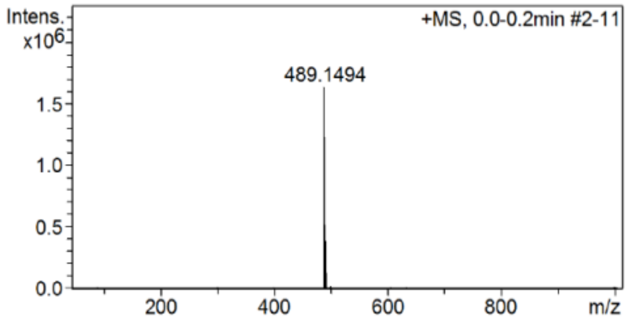

#	m/z	Res.	S/N	I	I %	FWHM
1	489.1494	31259	23608.5	1643158	100.0	0.0156

Figure S1. Dose–response curves of compounds B1–B10 in HCT116 and BEAS-2B cells used for IC_{50} determination.

	BEAS-2B 24h	HCT116 24h

B1

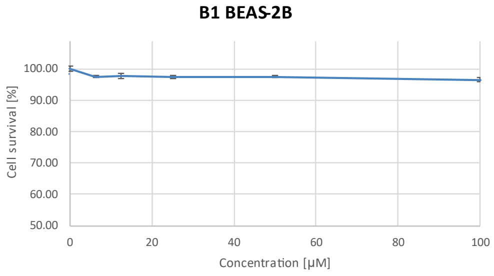

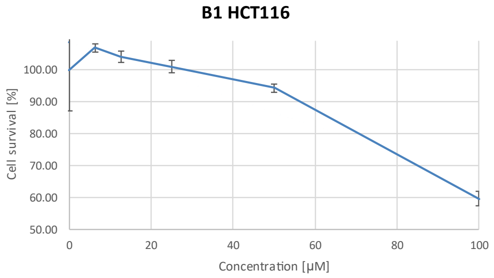

B2

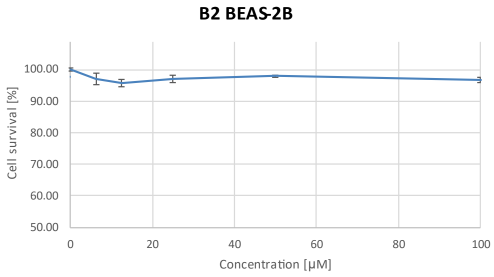

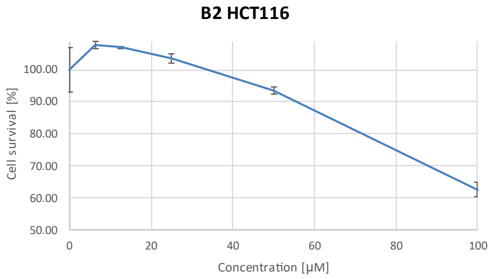

B3

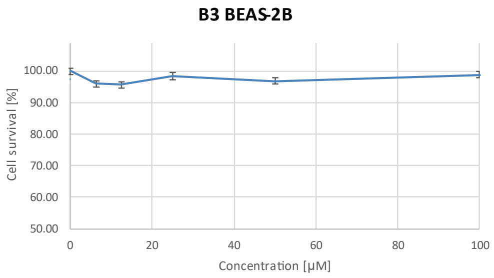

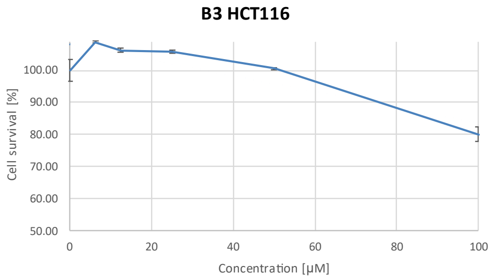

B4

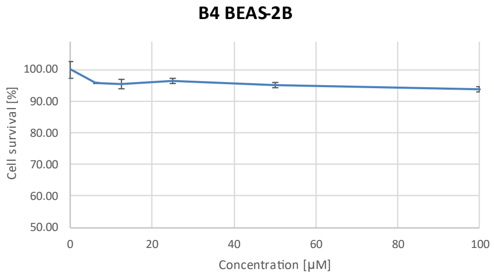

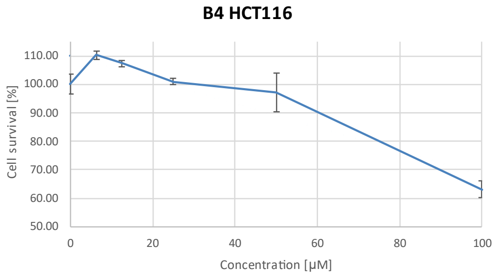

B5

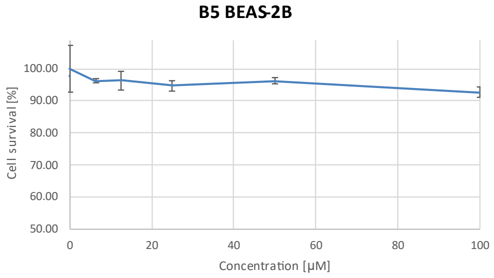

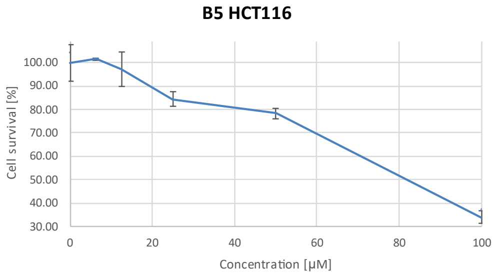

B6

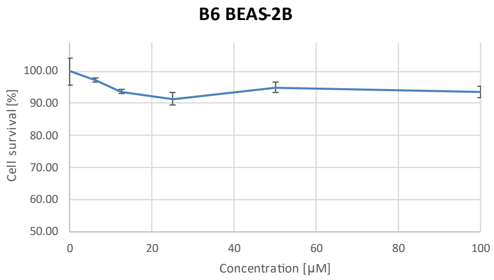

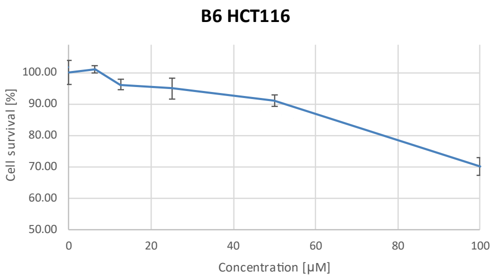

B7

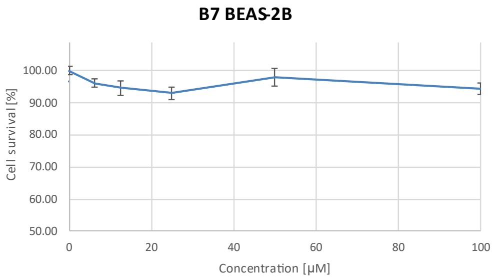

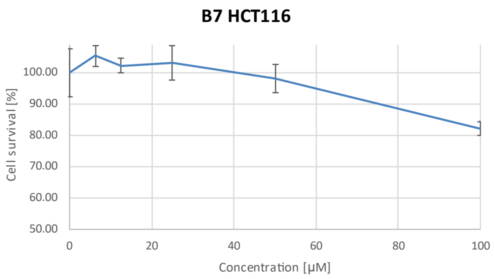

B8

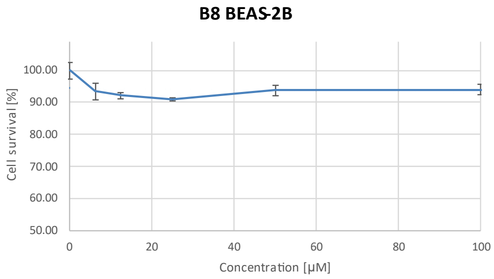

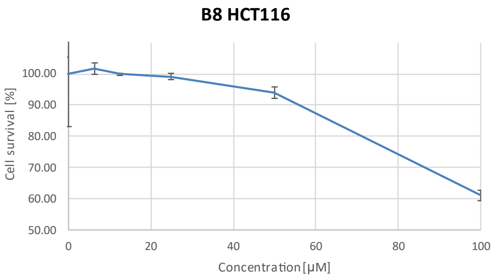

B9

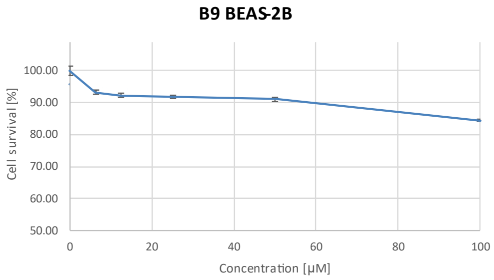

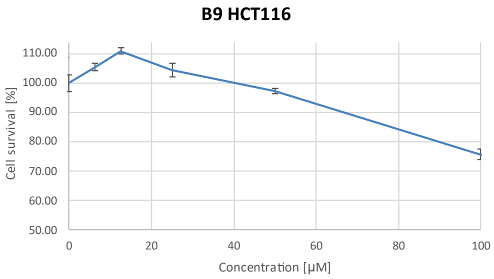

B10

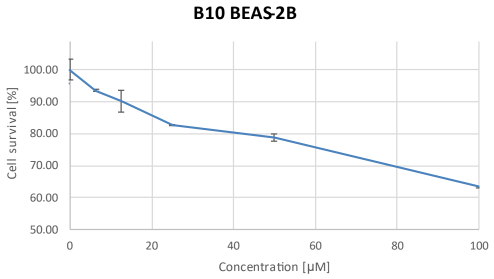

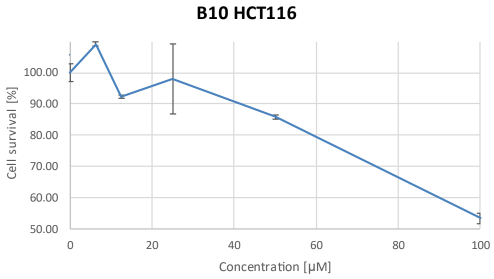

Figure S2. Representative cell cycle histograms obtained by flow cytometry.

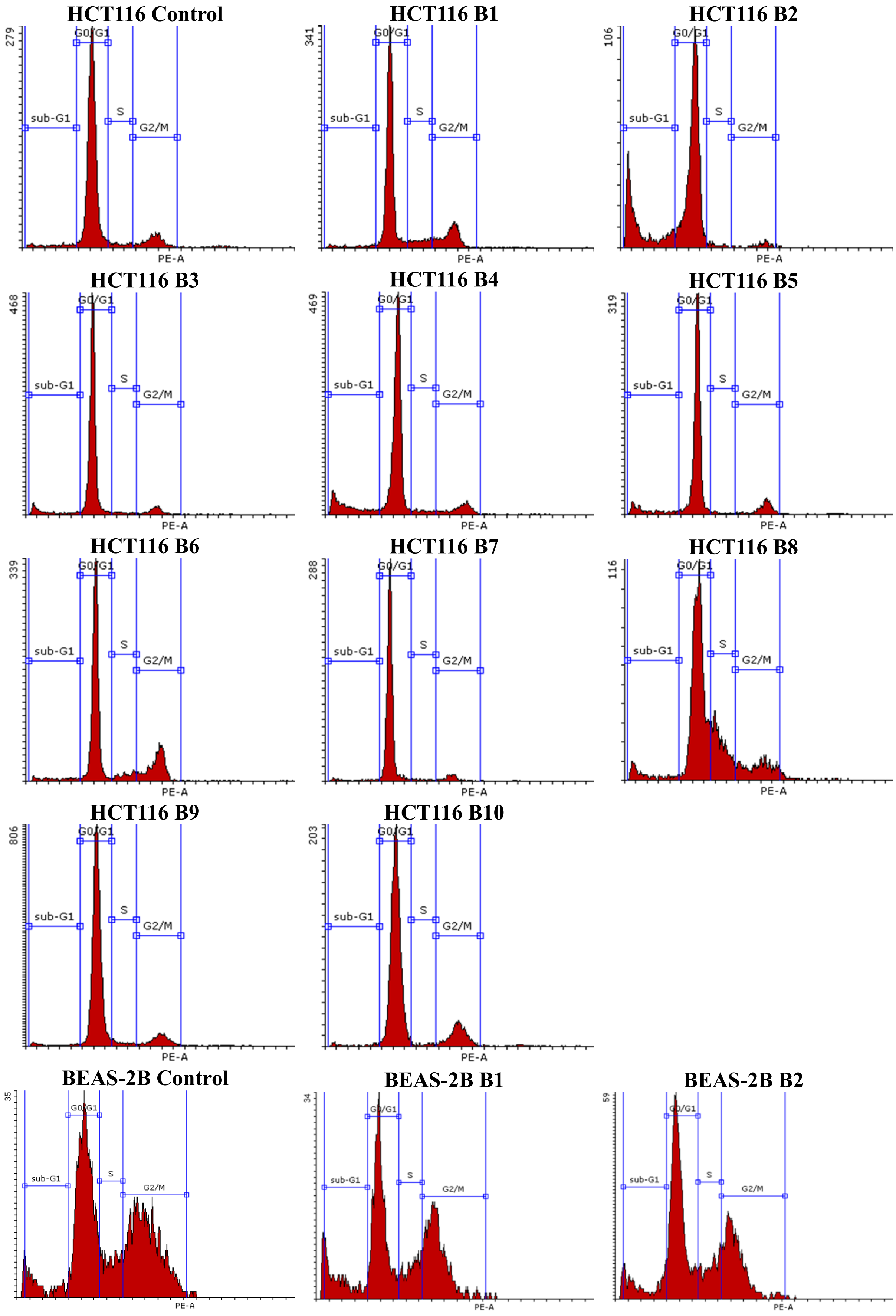

BEAS-2B B3

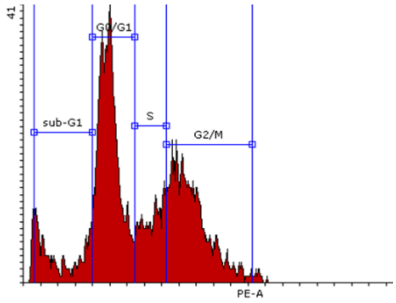

BEAS-2B B4

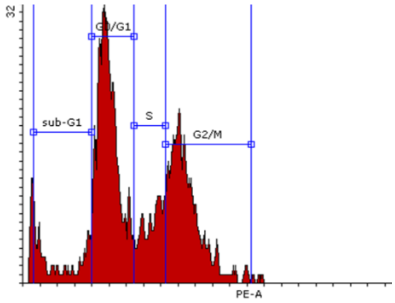

BEAS-2B B5

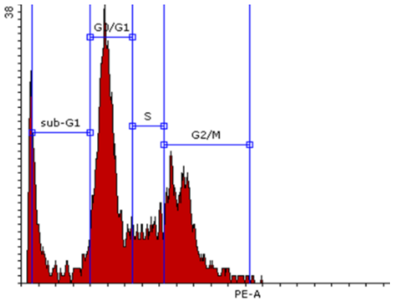

BEAS-2B B6

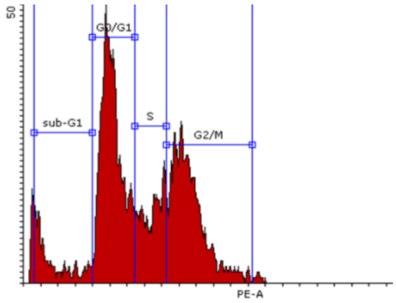

BEAS-2B B7

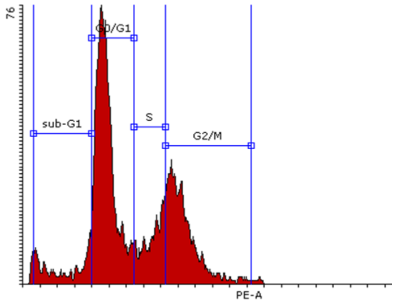

BEAS-2B B8

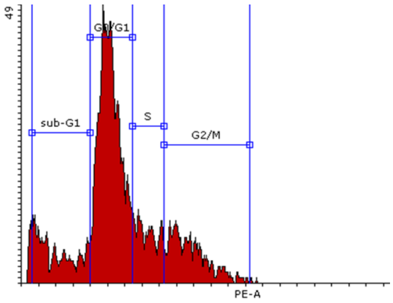

BEAS-2B B9

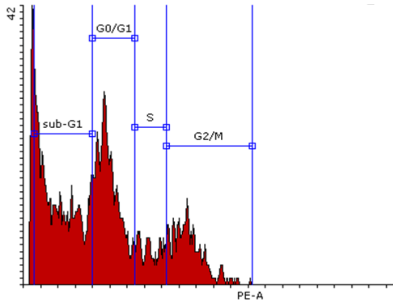

BEAS-2B B10

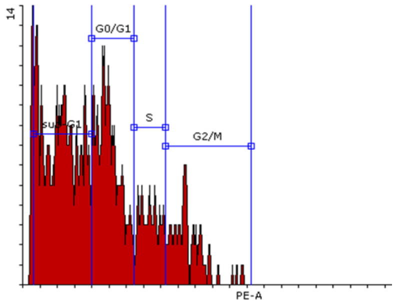

Figure S3. Representative Annexin V-FITC/PI dot plots obtained by flow cytometry.

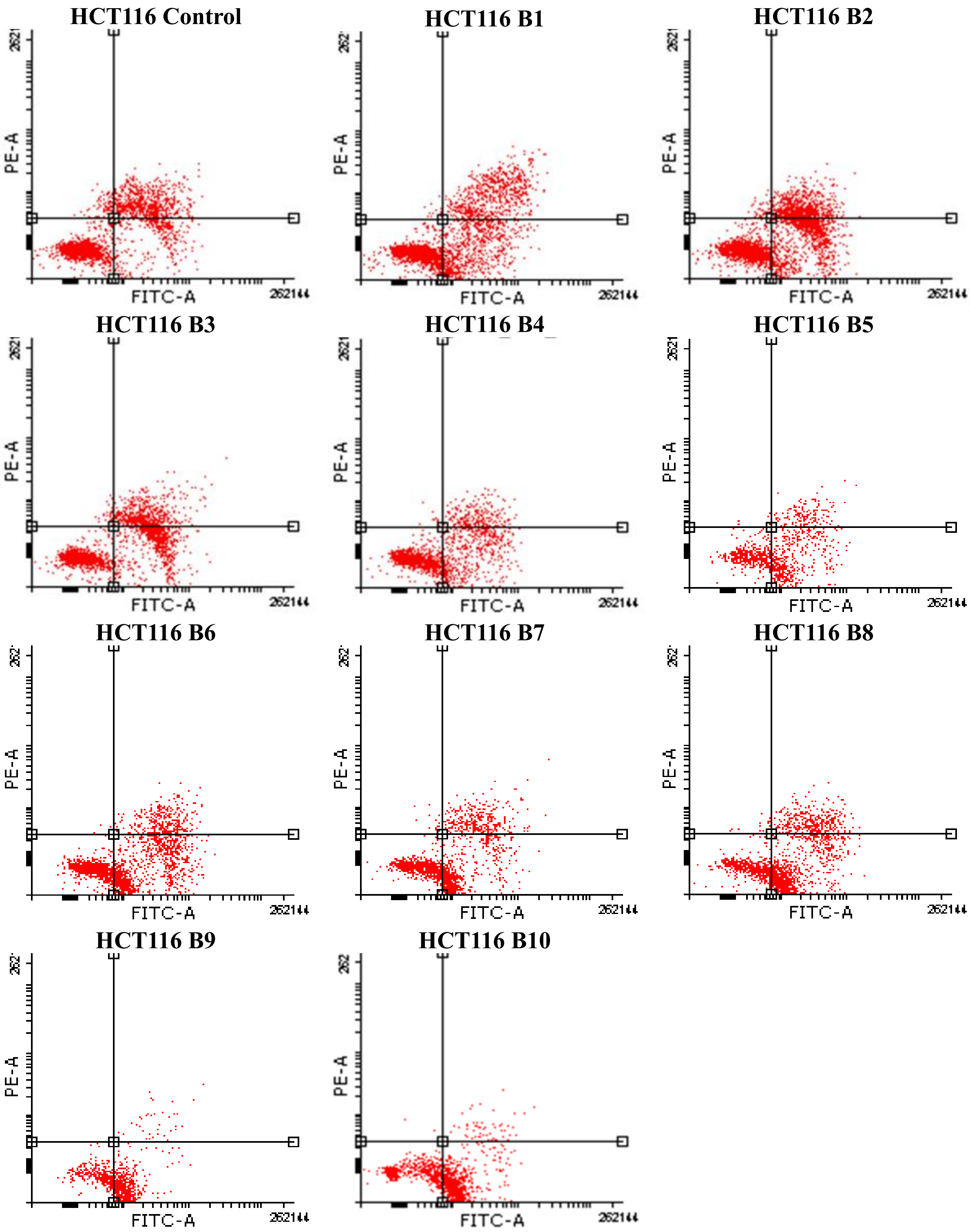

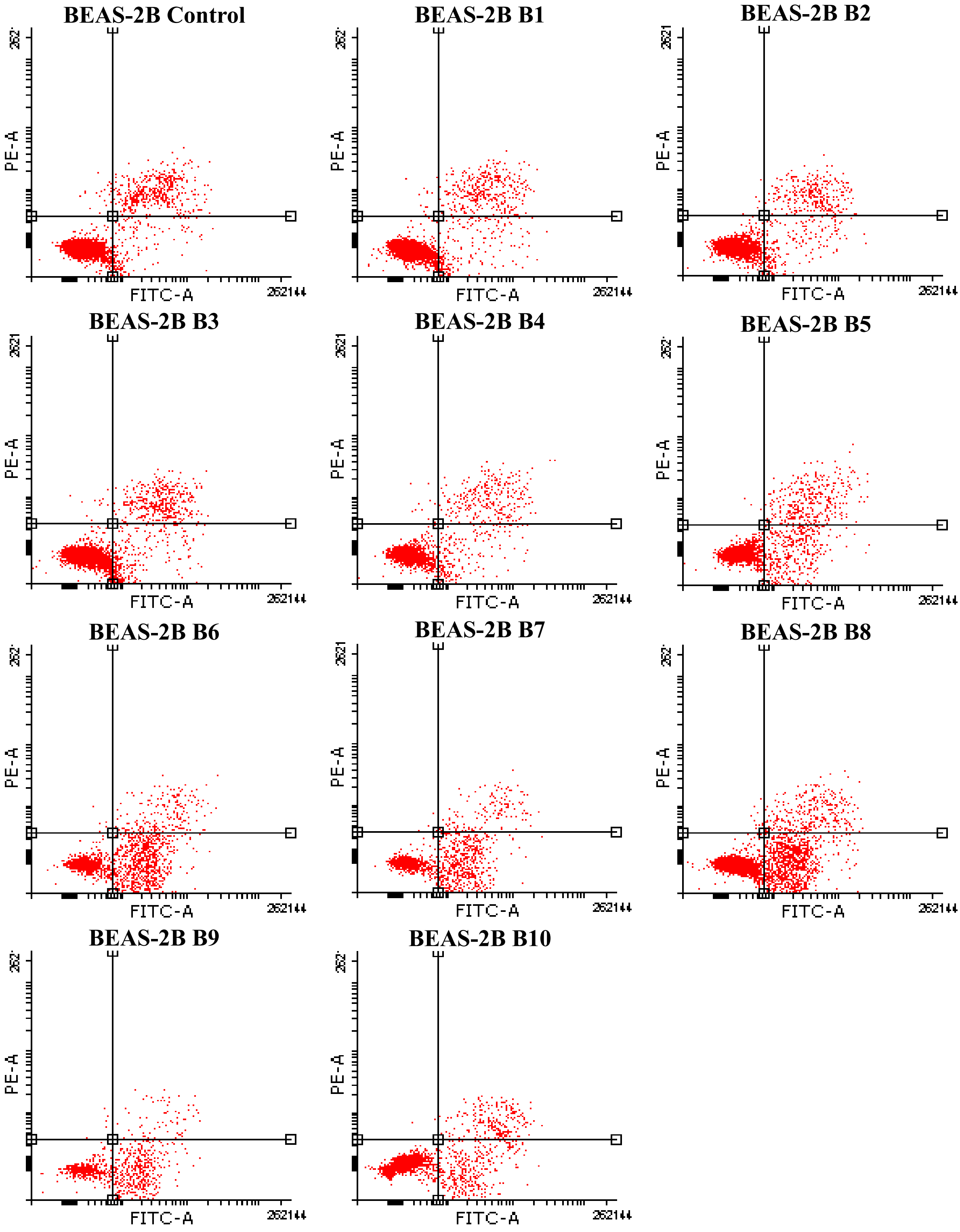
BEAS-2B Control
BEAS-2B B1
BEAS-2B B2
BEAS-2B B3
BEAS-2B B4
BEAS-2B B5
BEAS-2B B6
BEAS-2B B7
BEAS-2B B8
BEAS-2B B9
BEAS-2B B10
PE-A
FITC-A
262144

Figure S4. Representative ROS histograms obtained by flow cytometry.

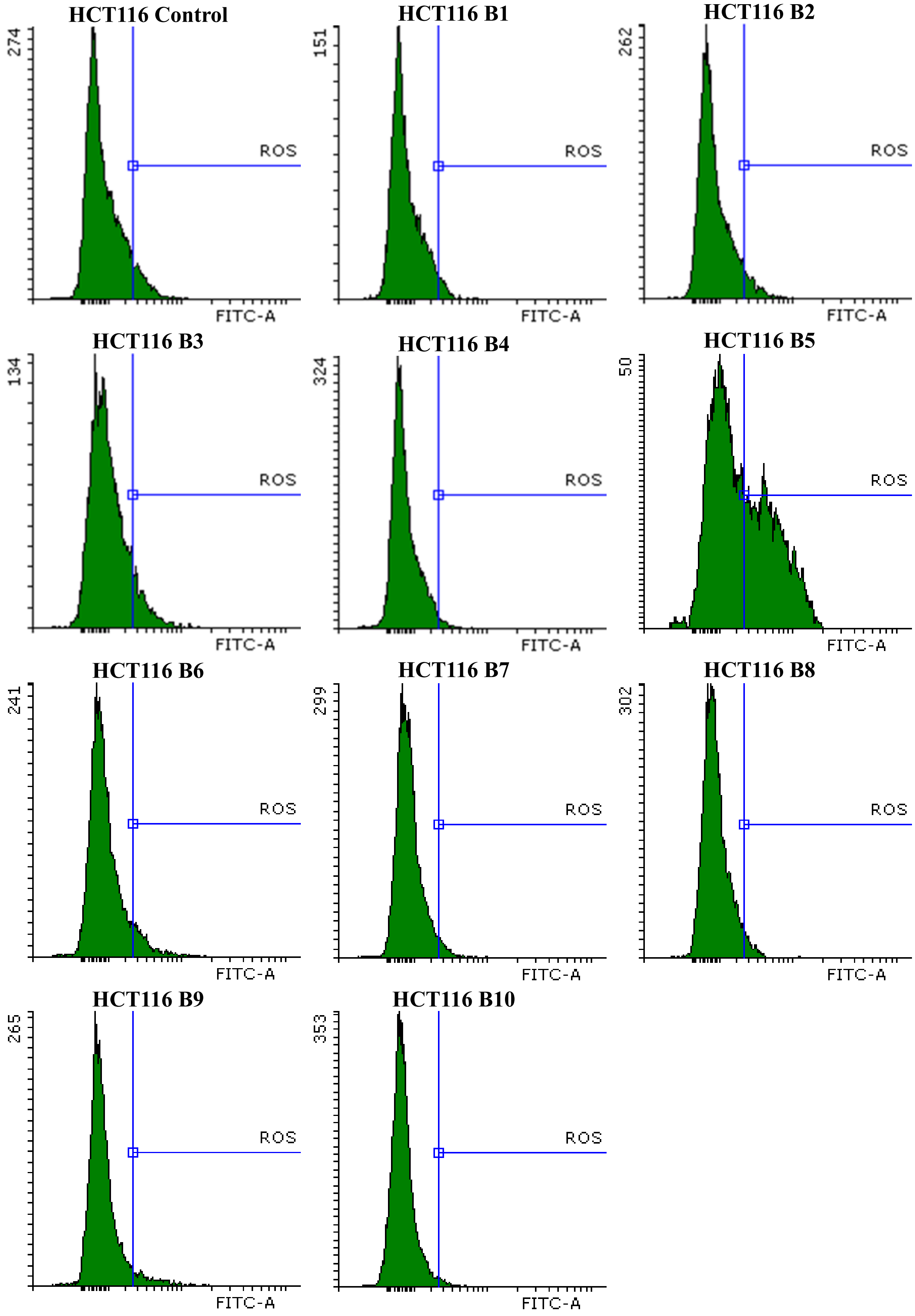

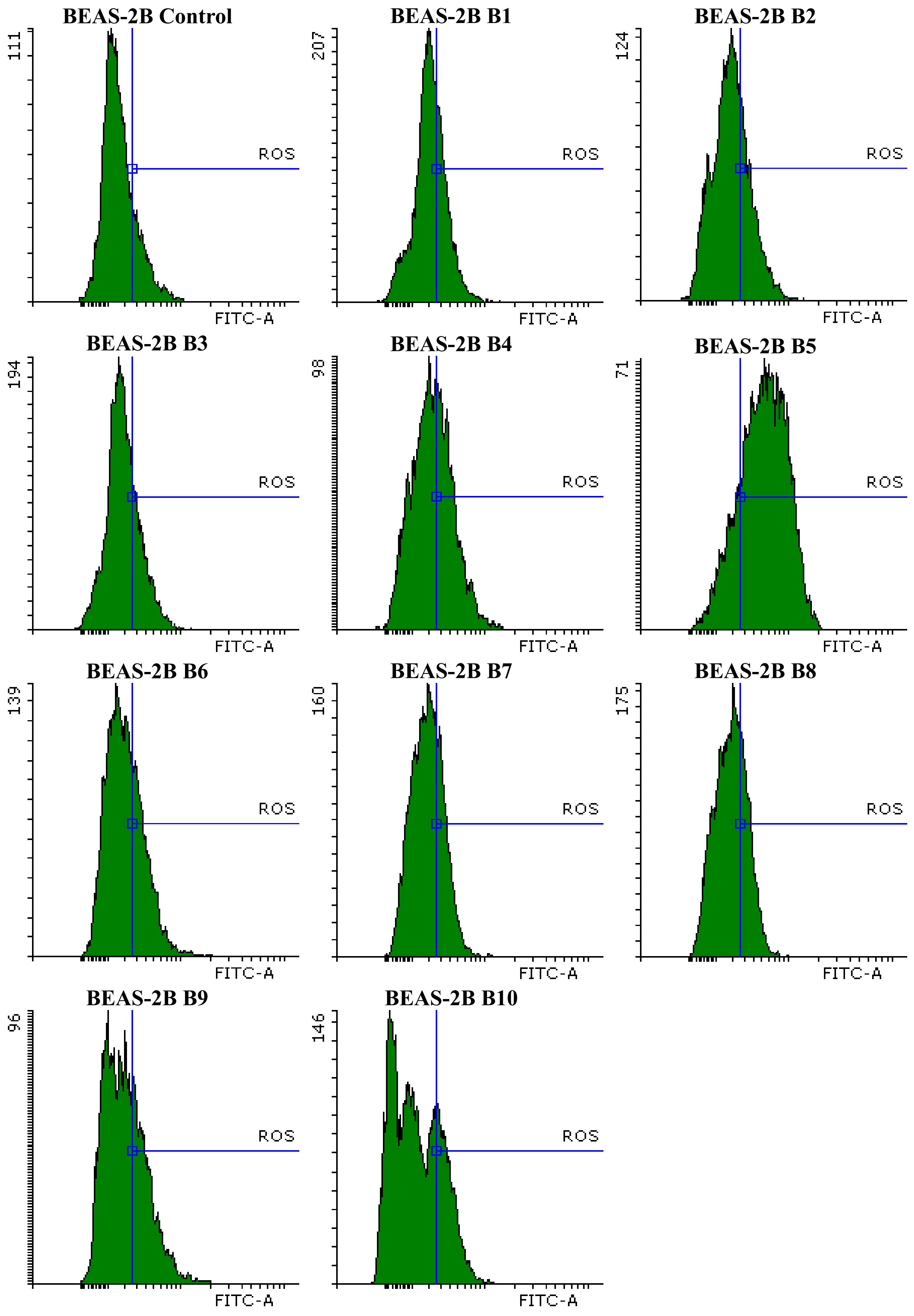
BEAS-2B Control
111
ROS
FITC-A
BEAS-2B B1
207
ROS
FITC-A
BEAS-2B B2
124
ROS
FITC-A
BEAS-2B B3
194
ROS
FITC-A
BEAS-2B B4
98
ROS
FITC-A
BEAS-2B B5
71
ROS
FITC-A
BEAS-2B B6
139
ROS
FITC-A
BEAS-2B B7
160
ROS
FITC-A
BEAS-2B B8
175
ROS
FITC-A
BEAS-2B B9
96
ROS
FITC-A
BEAS-2B B10
146
ROS
FITC-A
